# *Leptospira santarosai*: A Systematic Review on Its Serological Diversity, Geographical Distribution, Natural Sources of Infection, and Human Leptospirosis

**DOI:** 10.3390/microorganisms14061364

**Published:** 2026-06-18

**Authors:** Ronald Guillermo Peláez Sánchez, Jorge Emilio Salazar Flórez, Luz Estella Giraldo Cardona, Lina Paola Cifuentes, Daniela Sánchez Mejía, Santiago Pineda, Mariana Ossa-Yepes, Marco Torres-Castro, Alejandro Suarez-Galaz, Rodrigo Urrego, Luis Ernesto López-Rojas, Sergio Agudelo-Pérez, Fernando P. Monroy

**Affiliations:** 1 Infectious and Chronic Diseases Study Group (GEINCRO), School of Health Sciences, San Martin University, Sabaneta 055457, Colombia; jorge.salazarf@sanmartin.edu.co (J.E.S.F.); luz.giraldo@sanmartin.edu.co (L.E.G.C.); ipaocifuentes@gmail.com (L.P.C.); dalamejia@gmail.com (D.S.M.); 2 Department of Ecological Science, Vrije Universiteit Amsterdam, 1081 HV Amsterdam, The Netherlands; s.pinedagomez@student.vu.nl; 3 Research Group in Biology CES, Directorate of Science, Technology, and Innovation, Universidad CES, Antioquia 050021, Colombia; ossay.mariana@uces.edu.co; 4 Dr. Hideyo Noguchi Regional Research Center, Laboratory of Zoonoses and Other Vector-Borne Diseases, Autonomous University of Yucatan, Yucatan 97000, Mexico; antonio.torres@correo.uady.mx (M.T.-C.); alexrsg97@gmail.com (A.S.-G.); 5 INCA-CES Group, Faculty of Agricultural and Natural Sciences, Universidad CES, Medellín 050021, Colombia; rurrego@ces.edu.co; 6 Colombian Institute of Tropical Medicine (ICMT), Sabaneta 055450, Colombia; lelopez@ces.edu.co; 7 School of Medicine, Universidad de La Sabana, Chía 025001, Colombia; sergioagpe@unisabana.edu.co; 8 Department of Biological Sciences, Northern Arizona University, Flagstaff, AZ 86011, USA; fernando.monroy@nau.edu

**Keywords:** *Leptospira santarosai*, serological diversity, geographical distribution, natural sources of infection, and human leptospirosis

## Abstract

Leptospirosis is a globally distributed zoonotic disease caused by pathogenic bacteria of the *Leptospira* genus. Currently, 77 genomic species have been described. *Leptospira interrogans* is the most extensively studied species due to its high prevalence worldwide and the severity of the disease it causes in humans and animals. However, *Leptospira santarosai* is an important pathogenic species in the Americas, the Caribbean islands, and Taiwan. This species has a high serological diversity: it can infect domestic, wild, and agricultural production animals, causing reproductive problems and substantial economic losses. Additionally, *Leptospira santarosai* has been detected in water sources and wet soils. In humans, infection with this species can lead to a wide range of clinical manifestations and severe complications. Therefore, this study aimed to synthesize available information on the serological diversity, geographical distribution, natural sources of infection, and human leptospirosis caused by *Leptospira santarosai* to better understand their role in the leptospirosis transmission cycle. **Methods:** A systematic review of the literature was conducted, following the criteria established by the PRISMA-2020 guide, the search for scientific articles was conducted in five specialized and multidisciplinary databases (PubMed, Scopus, Web of Science, SciELO, and LILACS), and a search engine (Google Scholar). Two different search strategies (*Leptospira santarosai* OR *L. santarosai*) were used. **Result**: Once the search was carried out in the databases, 2989 scientific articles were identified. These articles underwent a process of identification, screening, eligibility, and inclusion, resulting in 84 articles that met all established inclusion criteria. These articles were included in the qualitative synthesis and elaboration of the systematic review. **Conclusions:** *Leptospira santarosai* shows a high serological diversity, with 14 serogroups and 59 serovars. The species has a wide geographic distribution, having been reported on five continents and in 26 countries, and has been described as an infectious agent in at least 24 host animals. It has also been detected in environmental sources such as water and wet soils; 24 serovars have been identified as the causative agents of human leptospirosis, causing clinical manifestations that range from mild to severe forms of the disease and clinical complications such as myocarditis, uveitis, and neuroleptospirosis. Although *L. santarosai* is considered native to the Americas, it shows an expansion pattern to other continents and countries. Therefore, this pathogenic species of the *Leptospira* genus represents an important public health problem worldwide.

## 1. Introduction

Leptospirosis is a zoonotic disease distributed worldwide, which is caused by pathogenic bacteria belonging to the *Leptospira* genus [1]. Previous epidemiological studies have estimated that 1.03 million cases and 58,900 deaths occur annually worldwide due to human leptospirosis [2]. Leptospirosis is considered a neglected disease, and its highest incidence can be found in tropical regions of developing countries [3]. In addition, leptospirosis is recognized as an emerging infectious disease due to large outbreaks reported in different regions of the world, often associated with environmental disasters and aquatic sporting events [4]. Severe clinical presentations, such as Weil’s syndrome and severe pulmonary hemorrhage syndrome (SPHS), have also been increasingly reported and are major causes of death in regions where the disease is endemic [5,6]. Currently, 77 genomic species of the *Leptospira* genus have been described, and their genomes are available in the NCBI-Genome database (https://www.ncbi.nlm.nih.gov/genome, accessed on 14 January 2026). These species have been classified into four main clades, pathogenic (P1), intermediate/pathogenic (P2), saprophytic (S1), and newly described saprophytic (S2), based on the phylogenetic analysis of 1371 orthologous genes representing the core genome shared by all species in the genus [7]. *Leptospira santarosai* belongs to clade P1 (*L. santarosai*, *L. adleri*, *L. ainazelensis*, *L. ainlahdjerensis*, *L. alexanderi*, *L. alstonii*, *L. barantonii*, *L. borgpetersenii*, *L. ellisii*, *L. gomenensis*, *L. gorisiae*, *L. interrogans*, *L. kirschneri*, *L. kmetyi*, *L. mayottensis*, *L. noguchii*, *L. sanjuanensis*, *L. stimsonii*, *L. tipperaryensis*, *L. weilii*, *and L. yasudae*). This clade includes the most virulent and pathogenic species, which are responsible for severe cases of leptospirosis in humans and animals. Historically, *Leptospira santarosai* was first isolated from the kidney of a spiny rat captured in the Panama Canal zone and was proposed as a new species in 1987 by Yasuda et al. [8]. This species was named in honor of the Brazilian scientist Carlos Santarosa, a veterinary microbiologist and pioneer in leptospirosis research in Brazil [8]. In parallel, a serological classification system for the *Leptospira* genus is also used and has been maintained due to the selectivity of some serovars to infect specific hosts in some geographic regions. The identification of Leptospira isolates at the serovar and serogroup levels is performed using the Cross-Agglutination Absorption Test (CAAT) and the Microscopic Agglutination Test (MAT). However, these techniques have several limitations. The MAT has low sensitivity during the acute phase of the disease, its interpretation is complex in endemic areas, it exhibits cross-reactivity between serogroups, vaccination can affect the results, the technique is complex to perform, and it requires at least two paired samples. Meanwhile, the CAAT requires a lengthy procedure, is technically complex and expensive, and its use is limited to the identification of isolates at the serovar level in a few specialized laboratories worldwide. Using this methodology, more than 268 serovars have been described (approximately 230 of which are recognized as pathogenic), which are grouped into at least 30 antigenically related serogroups [9]. *Leptospira santarosai* has a high serological diversity with 59 serovars, which are grouped into 14 serogroups. Knowing which *Leptospira* species and serovars circulate in a country is essential for understanding the epidemiology of the disease, designing more specific and sensitive diagnostic and typing tools, developing vaccines with improved protective efficacy, and linking serovars to their respective animal hosts. This information is also important for identifying infection sources and establishing more effective prevention and control measures. *Leptospira interrogans* is the most studied species of the *Leptospira* genus due to its high prevalence worldwide and the severity of the disease it causes in humans and animals [10]. However, other pathogenic species, such as *Leptospira santarosai*, have been described as important causative agents of leptospirosis worldwide [11]. This species is considered native to the American continent; however, cases have also been reported in Asia, Oceania, Europe, and Africa, suggesting a pattern of global dispersal [12,13,14,15]. *Leptospira santarosai* exhibits high serological diversity [14] and infects both domestic [16] and wild animals [17]. In farm animals such as cattle and horses, it can cause reproductive disorders that lead to significant economic losses [18,19]. Additionally, the presence of this species has been reported in environmental water sources and wet soils [20]. *Leptospira santarosai* can cause a wide range of clinical presentations including benign anicteric leptospirosis [21], Weil’s syndrome [22], and severe pulmonary hemorrhagic syndrome (SPHS) [23]. Furthermore, serious clinical complications such as myocarditis [24], uveitis [25], and neuroleptospirosis [26] have been reported. Although significant advances have been made in the study of *Leptospira santarosai*, the available information remains scarce and scattered. Therefore, it is important to synthesize the existing evidence through a systematic review to consolidate current knowledge of the species and identify gaps that require further investigation. Accordingly, the objective of this systematic review was to synthesize information on the serological diversity, geographical distribution, natural sources of infection, and human leptospirosis caused by *Leptospira santarosai*.

## 2. Materials and Methods

### 2.1. Study Type

This study consisted of a systematic literature review aimed at synthesizing the available information on the serological diversity, geographic distribution, environmental sources, and human leptospirosis caused by *Leptospira santarosai*. The search and selection strategy for scientific articles followed the phases of the PRISMA 2020 guidelines (Preferred reporting items for systematic reviews and metanalyses), which include identification, screening, eligibility, and inclusion [27].

#### 2.1.1. Identification

Two researchers independently performed the literature search, article selection, and data extraction. Discrepancies between the searches were resolved by a third researcher. Additionally, the remaining authors reviewed the consolidated results and participated in the writing of the systematic review. To identify relevant publications, the *Leptospira santarosai* term was used to find synonymous words using the controlled vocabulary systems DeCS (Descriptors in Health Sciences) and MeSH (Medical Subject Headings), which are used in health sciences to organize, index, and retrieve scientific information. The search for the terms “*Leptospira santarosai*” identified “*L. santarosai*” as the only synonymous term. Therefore, the algorithm “*Leptospira santarosai*” OR “*L. santarosai*” were used in the search strategy to ensure specificity. Scientific articles were searched in five specialized and multidisciplinary databases (PubMed, Scopus, Web of Science, SciELO, and LILACS) and in the search engine Google Scholar (See, Supplementary Table S1).

#### 2.1.2. Screening

Articles obtained through a search using the algorithm “*Leptospira santarosai*” OR “*L. santarosai*” (using the Boolean operator “OR”) were imported into a database created in Microsoft Excel^®^. Duplicate publications were removed and all records were registered. The following initial screening criteria were applied: the search terms had to appear in the title, abstract, or in the full text. Articles were not restricted by language, geographic area, or year of publication.

#### 2.1.3. Eligibility

Inclusion criteria

Studies reporting the molecular identification of *Leptospira santarosai* using techniques such as DNA/DNA hybridization and phylogenetic analysis were included. Additionally, studies that performed serological identification at the serogroup or serovar level using techniques such as the Microscopic Agglutination Test (MAT), Cross-Agglutinin Absorption Test (CAAT), Pulsed-Field Gel Electrophoresis (PFGE), and Multilocus Sequence Typing (MLST) were also included. Studies addressing one or more of the following topics were considered eligible: serological diversity, geographic distribution, natural sources of infection, and human leptospirosis caused by *L. santarosai*. Only original research articles published in indexed, peer-reviewed journals were included.

Exclusion criteria

Articles that were withdrawn, unavailable in databases, without full-text access, opinion pieces or editorials, drafts, conference abstracts, posters, and preprints were excluded. During the full-text screening, manuscripts were excluded if they did not mention *Leptospira santarosai* or *L. santarosai*, addressed the study topics only marginally, incorrectly identified the species at the molecular level, incorrectly identified the serogroup or serovar at the serological level, or lacked information on serological diversity, geographic distribution, environmental sources, or human leptospirosis caused by *Leptospira santarosai*.

#### 2.1.4. Included Studies

The literature search ended on 14 January 2026. The selected articles were downloaded in PDF format and stored in a database for subsequent review. Information related to the four variables analyzed (serological diversity, geographic distribution, environmental sources of infection, and human leptospirosis) was extracted from each article and recorded in a database created in Microsoft Word^®^ (Supplementary Tables S2–S7). The extracted information was organized, tabulated, and graphically summarized to facilitate analysis and synthesis of the available evidence. The protocol used for the systematic review was registered in the International Prospective Register of Ongoing Systematic Reviews (PROSPERO) under the code CRD420251058799 and is available at the following link: (https://www.crd.york.ac.uk/PROSPERO/view/CRD420251058799, accessed on 14 January 2026).

#### 2.1.5. Assessment of Methodological Quality and Risk of Bias

The type of study was determined for the 84 scientific articles selected for the systematic review. Depending on the study type of each article, an appropriate guide was selected for evaluating methodological quality and risk of bias. After applying the guide, each article was classified as high-quality, moderate-quality, or low-quality. Subsequently, the risk of bias for each scientific publication was determined, and the articles were classified as having high risk of bias, medium risk of bias, or low risk of bias. Finally, the strengths and weaknesses of each scientific article were highlighted. The methodological quality and risk of bias of the included studies were evaluated using the STROBE and ARRIVE guide. STROBE guide (Strengthening the Reporting of Observational Studies in Epidemiology) provides guidance for reporting observational studies such as cohort, case–control, and cross-sectional designs; and ARRIVE guide (Animal Research: Reporting of In Vivo Experiments) provides guidelines for reporting research involving animal experiments. The detailed procedure for the quality assessment process and risk of bias is described in detail in the (Supplementary Table S2).

## 3. Results

### 3.1. Search and Selection of Information

**Figure 1:**
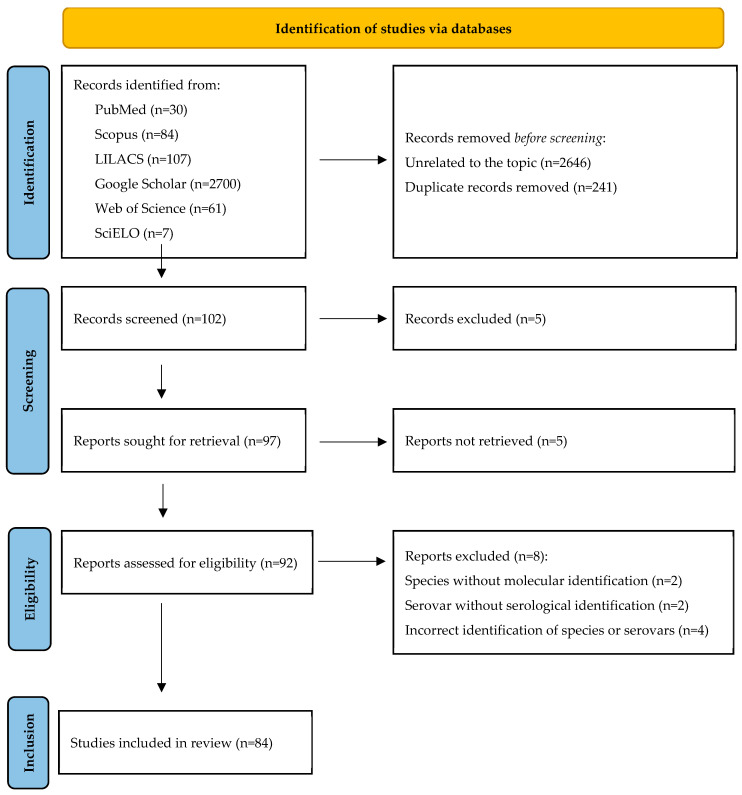
The figure shows the flow diagram used for the search of scientific articles related to *Leptospira santarosai*, following the identification, screening, eligibility, and inclusion stages recommended by the PRISMA 2020 guide for the preparation of systematic reviews.

The search and selection process identified 2989 records containing the terms “*Leptospira santarosai*” OR “*L. santarosai*” (PubMed = 30, Scopus = 84, LILACS = 107, Google Scholar = 2700, Web of Science = 61, and SciELO = 7). During the identification stage, records unrelated to the topic (n = 2646) and duplicate records (n = 241) were removed, leaving 102 records for screening. In the screening stage, the titles and abstracts of these records were evaluated, and 5 records were excluded (n = 5), leaving 97 records sought for retrieval. Of these, 5 reports could not be retrieved (n = 5), leaving 92 reports assessed for eligibility. During the eligibility stage, 8 articles were excluded due to species without molecular identification (n = 2), serovars without serological identification (n = 2), and incorrect identification of species or serovars (n = 4). Finally, 84 articles met the inclusion criteria and were included in the qualitative synthesis (Figure 1).

### 3.2. Evaluation of Methodological Quality and Risk of Bias

The included studies were classified according to their purpose as descriptive or analytical, according to the temporality as cross-sectional, according to the control of factors as experimental or observational, and according to the chronology as retrospective or prospective (Supplementary Table S2). The publications had a compliance range of the STROBE guide between 78–91%. In terms of the level of methodological quality, the distribution was as follows: 3 articles (very high quality—3.57%), 73 articles (high quality—86.9%), 7 articles (moderate to high quality—8.33%), and 1 article (moderate quality—1.19%). In terms of the risk of bias, the distribution was as follows: 4 articles (high risk of bias—4.76%), 7 articles (medium to high risk of bias—8.33%), 68 articles (medium risk of bias—80.95%), and 5 articles (medium to low risk of bias—5.95%) (Table 1 and Supplementary Table S2).

### 3.3. Serological Diversity of Leptospira santarosai

**Figure 2:**
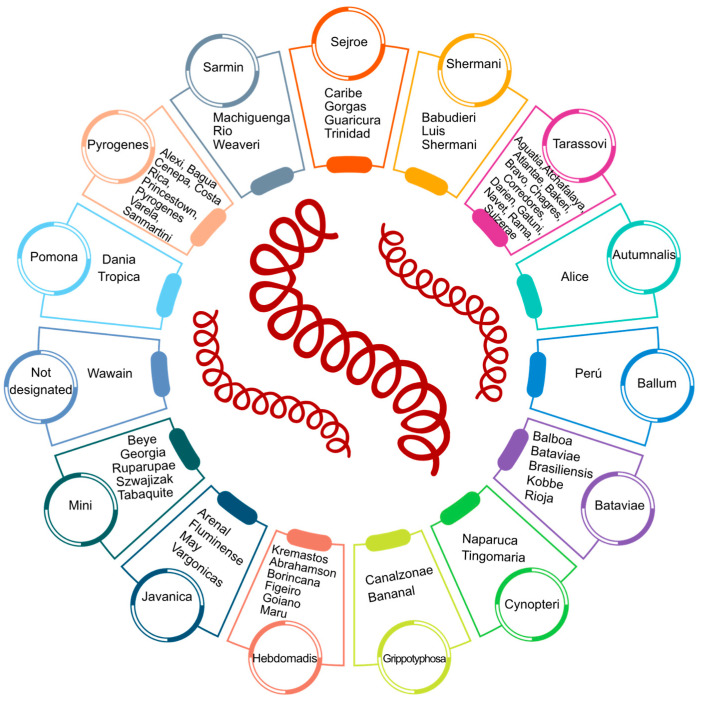
Serological classification of *Leptospira santarosai*, including 59 serovars grouped into 14 serogroups. Serogroup names are represented by circles and serovar names by squares. The serovar Wawain has not been assigned to a serogroup.

The first specific objective of this systematic review was to describe serological diversity of *Leptospira santarosai*. According to the information available in the Medical Reference Center for Leptospirosis (https://leptospira.amsterdamumc.org/filters/species/l-santarosai/, accessed on 14 January 2026), described by Brenner et al. in 1999 [14], NCBI-Taxonomy (https://www.ncbi.nlm.nih.gov/Taxonomy/, accessed on 14 January 2026), and subsequent publications [38,42,43,44,52,68,73,74], a total of 59 serovars have been described. As a result of this systematic review, these 59 serovars were systematically organized into 14 serogroups, providing a synthesis of the current knowledge on the serological diversity of this species (Table 2 and Figure 2).

### 3.4. Geographical Distribution of Leptospira santarosai

**Figure 3:**
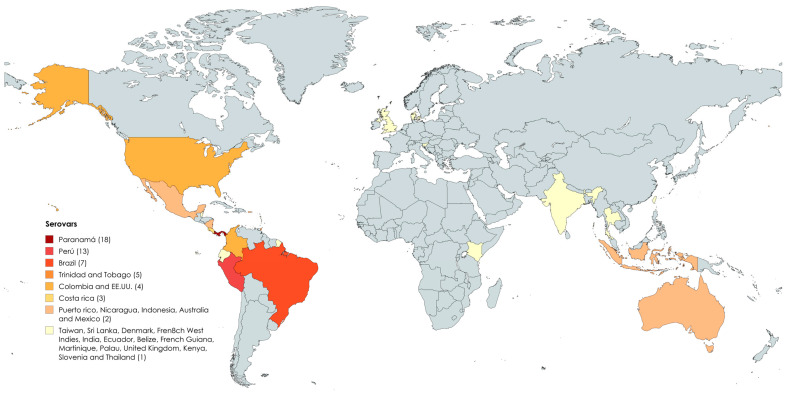
Geographic distribution of *Leptospira santarosai*. The map highlights in color the countries where the species has been reported. The range of red shades represents the number of reported serovars per country, with darker shades indicating higher numbers of serovars and lighter shades indicating lower numbers. Gray indicates countries where *L. santarosai* has not been reported. Overall, the species has been reported on five continents and in 26 countries. The map was created using the web server (https://mapchart.net/world.html, accessed on 14 January 2026).

As a second objective, the geographic distribution of *Leptospira santarosai* was described, revealing its presence on five continents (excluding Antarctica) [12,13,14,51,79]. The continent with the highest number of reports of *Leptospira santarosai* was America, followed by Asia, Europe, Oceanica, and Africa. In America, the species has been reported in Panama, Peru, Brazil, Trinidad and Tobago, Colombia, USA, Costa Rica, Puerto Rico, Nicaragua, French West Indies, Mexico, Ecuador, Belize, French Guiana, and Martinique. In Asia, it has been reported in Indonesia, Taiwan, Sri Lanka, Thailand, and India. In Europe, the species has been reported in Denmark, Slovenia, and the United Kingdom. In Oceania, it has been reported in Australia and Palau, while, in Africa, it has been reported in Kenya. The geographic distribution of serovars associated with *Leptospira santarosai* showed that 59 serovars have been identified across 26 countries and territories. However, in seven countries, isolates were identified only at the species level. Panama reported the highest number of serovars (18), followed by Peru (13) and Brazil (7) (Figure 3 and Supplementary Table S4).

### 3.5. Animals Infected by Leptospira santarosai

**Figure 4:**
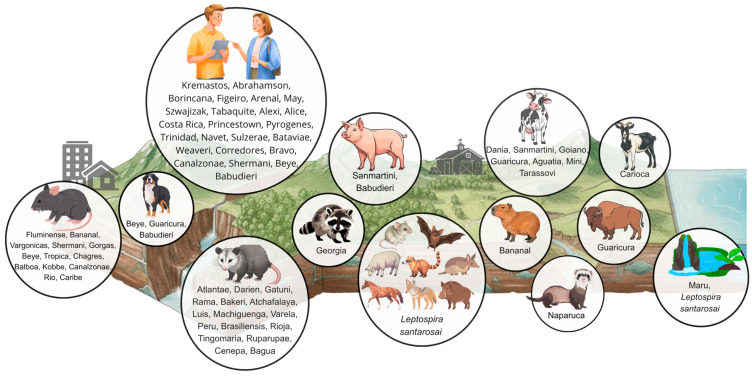
Hosts, accidental hosts, and environmental sources in which *Leptospira santarosai* has been detected (circles). Associated serovars and their host associations are also shown. The background depicts an urban environment with humans, dogs, and rodents, and a jungle environment with wild animals. Rodents are present in both environments. The figure was generated using Google Gemini^®^ (https://gemini.google.com/app?hl=es, accessed on 14 January 2026) and edited in Canva^®^ (https://www.canva.com/es_419/, accessed on 14 January 2026).

The third objective was to relate *Leptospira santarosai* serovars to their animal hosts. A total of 45 serovars were reported infecting 24 hosts, including the following: *Galictis cuja*, pigs (*Sus scrofa domesticus*) [52], rodents (*Rodentia*) [52], raccoons (*Procyon lotor*) [13], cattle (*Bos taurus*) [59], opossums (Didelphimorphia) [13], capybaras (*Hydrochoerus hydrochaeris*) [38], buffalo (*Bubalus bubalis*) [73], dogs (*Canis lupus familiaris*) [30,31,50,76,89], goats (*Capra aegagrus hircus*) [74], coatis (*Nasua*) [16], coyotes (*Canis latrans*) [75], equids (Equidae) [18], sheep (*Ovis aries*) [62], *Marmosops ocellatus* [49,55], *Marmosops paulensis* [49,55], *Metachirus myosuros* [49,55], *Metachirus nudicaudatus* [49,55], *Monodelphis glirina* [49,55], *Monodelphis peruviana* [49,55], nine-banded armadillos (*Dasypus novemcinctus*) [16], *Nectomys squamipes* [52], wild boar (*Sus scrofa*) [35], and bats [78]. Among these hosts, opossums (Didelphimorphia) were associated with the highest number of serovars (16), followed by rodents (13) and cattle (7). Additionally, 13 isolates were identified only at the species level (Figure 4 and Supplementary Table S5). According to the results obtained, three transmission cycles of *Leptospira santarosai* can be identified: a transmission cycle involving wild animals, including *Galictis cuja*, raccoons (*Procyon lotor*), opossums (*Didelphimorphia*), capybaras (*Hydrochoerus hydrochaeris*), coatis (*Nasua*), coyotes (*Canis latrans*), *Marmosops ocellatus*, *Marmosops paulencis*, *Metachirus myosuros*, *Metachirus nudicaudatus*, *Monodelphis glirine*, *Monodelphis peruviana*, nine-banded armadillos (*Dasypus novemcinctus*), *Nectomys squamipes*, wild boar (*Sus scrofa*), and bats; a transmission cycle involving domestic animals, including dogs (*Canis lupus familiaris*), pigs (*Sus scrofa domesticus*), cattle (*Bos taurus*), buffalo (*Bubalus bubalis*), sheep (*Ovis aries*), equids (*Equidae*), and goats (*Capra aegagrus hircus*); and a transmission cycle involving synanthropic animals, represented by rodents (*Rodentia*). Regarding potential transmission routes, infections of different animal species with the same serovar have been reported. For example, the serovar Bananal has been identified in rodents and capybaras, Shermani in rodents and humans, Beye in rodents, dogs, and humans, Canalzonae in rodents and humans, Sanmartini in cattle and pigs, Babudieri in pigs, dogs, and humans, and Guaricura in cattle, buffalo, and dogs. These findings reflect the ability of some serovars to infect multiple animal hosts. Additionally, rodents, dogs, and pigs may act as sources of infection for humans because they share the same serovars.

### 3.6. Leptospira santarosai and Natural Sources of Infection

The fourth specific objective of the systematic review was to evaluate the association of *Leptospira santarosai* with environmental sources. The species has been reported in wet soils, lakes, and waterfalls. However, only one environmental isolate has been identified at the serovar level (*Leptospira santarosai* serovar Maru), which was detected in water sources in the Panama Canal area (Figure 4 and Supplementary Table S5) [12,19,45,54,82,84,88,94].

### 3.7. Leptospira santarosai and Human Leptospirosis

**Figure 5:**
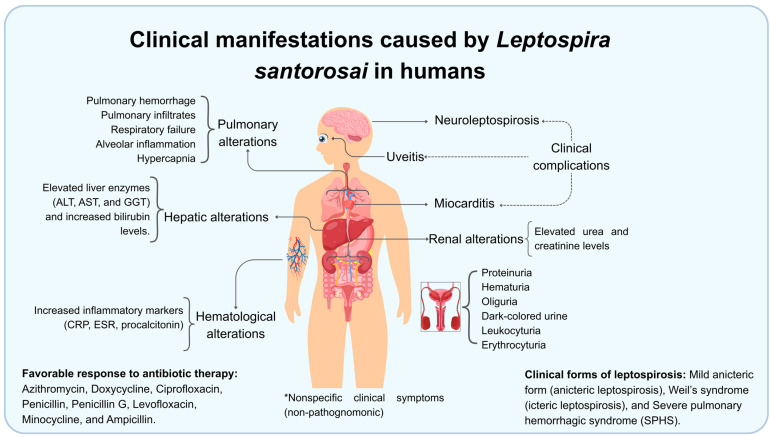
Clinical manifestations of *Leptospira santarosai* infection in humans, including pulmonary, hepatic, renal, and hematological alterations. Clinical forms of the disease, associated complications, and antibiotics reported for successful treatment of the patients are also shown.

The fifth objective was to identify *Leptospira santarosai* as a causative agent of human leptospirosis and to describe the clinical manifestations associated with infection. This species has been reported as an infectious agent in humans, with 24 isolates identified at the serovar level (Kremastos, Abrahamson, Borincana, Figeiro, Arenal, May, Szwajizak, Tabaquite, Alexi, Alice, Costa Rica, Princestown, Pyrogenes, Trinidad, Navet, Sulzerae, Bataviae, Weaveri, Corredores, Bravo, Canalzonae, Shermani, Beye, and Babudieri) [13,14,20,21,22,23,24,25,28,29,32,33,40,41,44,47,51,60,67,68,69,72,79,81,82,83,85,90,96]. The infection has been associated with clinical presentations such as benign anicteric leptospirosis, Weil’s syndrome, and severe pulmonary hemorrhagic syndrome (SPHS) [21,22]. Additionally, complications include myocarditis [23], uveitis [24], and neuroleptospirosis [25,96] have been reported (Figure 5 and Supplementary Table S6). Laboratory findings included urinary abnormalities such as proteinuria, hematuria, leukocyturia, dark-colored urine, leukocyturia, erythruria, and oliguria. Renal alterations include increased urea and creatinine levels. Hematological findings include elevated C-reactive protein (CRP), erythrocyte sedimentation rate (ESR), and procalcitonin levels. Hepatic involvement has been associated with increased liver enzyme levels, including alanine aminotransferase (ALT), aspartate aminotransferase (AST), gamma-glutamil transferasa (GGT), and bilirubin. Pulmonary alterations such as pulmonary hemorrhage, pulmonary infiltrates, respiratory failure, alveolar inflammation, and hypercapnia have also been described. However, no single clinical sign has been pathognomonic for the disease [11,20,21,22,23,30,31,33,44,50,51,59,69,72,76,79,81,83,89,96]. Additionally, *Leptospira santarosai* has been reported to be susceptible to several antibiotics, including azithromycin, doxycycline, ciprofloxacin, penicillin, levofloxacin, minocycline, and ampicillin, resulting in rapid recovery of the patients [40,83,90] (Figure 5 and Supplementary Table S7). Regarding the frequency of clinical presentations, *Leptospira santarosai* was most frequently associated with the benign anicteric form of leptospirosis (8 cases), followed by Weil’s syndrome (3 cases), and Severe Pulmonary Hemorrhagic Syndrome (SPHS) (3 cases). These findings suggest that this species may be less virulent than other species such as *Leptospira interrogans*, and may be better adapted to the human host, since at least 24 serovars with the potential to infect humans have been described. Regarding the frequency of clinical complications, neuroleptospirosis (3 cases), uveitis (2 cases), and myocarditis (1 case) were the most frequently reported. However, these complications were more frequently associated with immunocompromised patients. Regarding the diagnostic relevance of the clinical signs and symptoms, no pathognomonic feature specific to the diagnosis of leptospirosis was identified. Fever and myalgia were the only symptoms reported in most patients, although both were nonspecific. Laboratory findings were highly variable and lacked diagnostic specificity, highlighting the need for serological and molecular tests to confirm infection. It is worth mentioning that marked alterations in laboratory parameters may be associated with the development of severe clinical complication. Overall additional research is needed to better understand the pathophysiology of *Leptospira santarosai* infection.

## 4. Discussion

According to the results of this systematic review, *Leptospira santarosai* is a pathogenic species that exhibits wide serological diversity, with at least 59 serovars grouped into 14 serogroups. This broad intraspecific serological diversity underscores the epidemiological importance of this species and likely reflects its capacity to adapt to a wide range of environments. Indeed, *Leptospira santarosai* has been reported in at least 24 animal hosts, environmental sources such as water bodies, lakes, waterfalls, and wet soils, and humans, for whom 24 infective serovars have been described. Understanding the serological diversity of *Leptospira santarosai* is crucial and presents several challenges in public and veterinary health. From a diagnostic perspective, serological tests, such as the Microscopic Agglutination Test (MAT) depend on including local serovars to be accurate. If the serovars present in a region are unknown, false-negative results may occur. Epidemiologically, identifying circulating serovars allows for a better understanding of transmission patterns, animal reservoirs, and environmental risk factors. Regarding prevention and control, vaccines against *Leptospira* generate serovar-specific immunity only against the serovar included in the vaccine formulation. Therefore, knowledge of serological diversity is essential for the development of effective vaccines and control strategies adapted to local conditions. Additionally, the broad serological diversity of *Leptospira santarosai* has important public health implications. Different serovars can persist in the environmental for prolonged periods and be transmitted through water, wet soils, or animal hosts, increasing the risk of outbreaks. From an evolutionary perspective, the presence of numerous serovars likely reflects a long history of adaptation to diverse environments and hosts, resulting in the diversification of the genetic content and, consequently, the serological phenotype of the species. These findings also highlight important knowledge gaps regarding the ecological determinants of *Leptospira santarosai*. Future studies should investigate the environmental factors that influence the presence, distribution, and dynamics of this species across different ecosystems. According to the list of serovars reported by Brenner et al. in 1999 [14], the pathogenic species with the highest number of serovars in the genus *Leptospira* are *L. interrogans* (91 serovars), *L. santarosai* (59 serovars), *L. borgpetersenii* (49 serovars), *L. kirschneri* (39 serovars)*, L. noguchii* (18 serovars)*,* and *L. weilii* (15 serovars). Therefore, *Leptospira santarosai* is the second most serologically diverse species currently recognized within the genus and is consequently of considerable epidemiological, environmental, and clinical importance.

*Leptospira santarosai* has a wide geographic distribution and has been reported on five continents and 26 countries [12,13,14,51,70,79,91,92,93]. Reports of this species are most frequent in the American continent, particularly in Central America, South America, and the Caribbean islands. Panama reported the highest number of serovars (18), followed by Peru (13) and Brazil (7). However, the greater number of serovars reported in these countries likely reflects a higher sampling effort and a larger number of studies rather than the true serological diversity of the species. Therefore, it is necessary to carry out further research in countries where information is currently unavailable to determine the actual serological diversity and distribution of *Leptospira santarosai*. Additionally, in Taiwan, *Leptospira santarosai* serovar Shermani has been reported as one of the most prevalent serovars associated with human infection. These observations suggest that this species represents an important etiological agent of leptospirosis in various regions of the world [12]. Furthermore, 45 serovars were reported exclusively in a single country, whereas eight serovars were detected in more than one country: Canalzonae (Panama and Colombia), Beye (Panama and Colombia), Pyrogenes (Panama and Indonesia), Babudieri (Peru and Colombia), Tabaquite (Trinidad and Tobago, and the French Wes Indies), Alice (Sri Lanka and Colombia), Shermani (Panama and Taiwan), and Princestown (Trinidad and Tobago, and the USA). These findings indicate that, while many *Leptospira santarosai* serovars appear to have restricted geographic distributions, some are detected across multiple countries, showing a pattern of dispersion among the countries. When comparing geographical regions, the Americas reported the highest number of countries with the presence of *Leptospira santarosai* (15 countries and 57 serovars), followed by Asia (5 countries and 6 serovars), Europe (3 countries and 1 serovar), Oceania (2 countries and 2 serovars), and Africa (1 country, without identification of serovars). Regarding possible distribution routes, dispersion patterns can be observed among countries that share the same serovars. One group includes Panama, Colombia, Peru, Sri Lanka and Indonesia, whereas another includes Trinidad and Tobago, the United States, and the French West Indies. However, clonal dispersal studies among the serovars are required to verify these proposed distribution patterns. It is also important to acknowledge the potential for selection bias, as many countries lack taxonomic studies capable of identifying *Leptospira santarosai* and its serovars. Consequently, the current distribution patterns may reflect differences in research efforts and taxonomic resolution rather than the true geographic distribution of the species.

The evidence indicates that 39 serovars of *Leptospira santarosai* infect 24 hosts [13,16,18,30,31,34,35,38,46,48,49,50,52,53,55,56,57,58,59,61,62,63,64,65,66,73,74,75,76,77,80,86,87,89]. This serological diversity suggests that the species is associated with multiple hosts. Several serovars were reported infecting the same animal species (which is consistent with the theory that some serovars selectively infect certain animal hosts), while other serovars were identified infecting several animal hosts. For example, the serovar Georgia has been reported in *Procyon lotor*, whereas the serovar Carioca has been reported in *Capra aegagrus hircus*. In contrast, several serovars have been detected in multiple animal species, including Sanmartini in pigs and cattle, Babudieri in dogs and pigs, Bananal in capybaras and rodents, Beye in rodents and dogs, and Guaricura in cattle, buffalo, and dogs. These observations demonstrate that certain serovars may circulate among multiple host species, suggesting the possibility of interspecies transmission. The presence of *Leptospira santarosai* in agriculturally important hosts such as cattle, buffalo, goats, and pigs highlight the potential role of livestock in the epidemiology of leptospirosis caused by this species. These observations suggest that animal hosts other than rodents may contribute to the transmission cycle of the disease. Furthermore, it is evident that agricultural activities may also represent a risk factor for leptospirosis caused by this species, given its high capacity to infect various agriculturally important hosts such as cattle, buffalo, goats, and pigs. These observations suggest that livestock may contribute to transmission in both rural and per-urban settings. Reports of infection in domestic animals such as dogs indicate that these animals may also represent potential sources of exposure for humans. In addition, infections detected in wild animals suggest the existence of a sylvatic cycle that may represent another potential source of human infection when people enter these habitats. Several serovars have been reported in both animals and humans, including Canalzonae (rodents and humans), Babudieri (pigs, canines, and humans), Shermani (rodents and humans), Beye (rodents, canines, and humans). This overlap suggests that these hosts may act as a potential risk factor for human infection. Overall, these findings indicate that *Leptospira santarosai* may circulate across wildlife, livestock, and domestic animal populations. Human activities such as agriculture and environmental disturbance may increase contact with infected hosts, thereby increasing the risk of infection.

Furthermore, at least seven studies have reported the presence of *Leptospira santarosai* in various environmental sources such as soils, lakes, and waterfalls [12,19,45,54,84,88,94]. However, only one study identified the isolates at serovar level (*Leptospira santarosai* serovar Maru in water sources from the Panama Canal Zone). This finding demonstrates the ability of this species to persist in the environment. These observations highlight the need for additional studies detecting the presence of pathogenic *Leptospira* species in environmental sources to determine their role in the environmental persistence of the bacteria, evaluate whether environmental replication occurs, and determine whether water and soil act as effective vehicles for disease transmission. Moreover, the detection of *Leptospira santarosai* in water sources could be associated with the infection in non-traditional exposure contexts, such as environmental disasters, recreational water activities, and aquatic events. These scenarios have previously been identified as risk factors for infection by other *Leptospira* species [36,37,39,71,95,97,98,99,100]. The limited number of reports of *Leptospira santarosai* from environmental sources likely reflects both the small number of studies conducted and the methodological challenges associated with the detection and isolation of *Leptospira* from environmental samples. These challenges include the complexity of environmental matrices, which contain DNA from multiple organisms; the presence of substances that inhibit molecular assays, such as humic compounds, heavy metals, and organic matter; the low concentration of bacteria in environmental samples, resulting in reduced detection rates; and the high microbial diversity present in these environments, which requires the use of highly specific molecular markers. Additional limitations include the presence of degraded or fragmented DNA, which can lead to false-negative results, and the lack of standardized protocols for DNA extraction from different environmental matrices. Furthermore, the detection of bacterial DNA does not necessarily indicate the presence of viable or metabolically active organisms, which may lead to inaccurate conclusions regarding environmental transmission risks.

At least 24 serovars belonging to *Leptospira santarosai* have been identified as causative agents of human leptospirosis. The wide variety of serovars reported in human infections demonstrates the presence of multiple animal reservoirs and environmental sources contaminated with the bacterium, which may act as sources of infection for humans. According to the World Health Organization (WHO) and the International Leptospirosis Society (ILS), the disease presents in four broad clinical categories: a mild influenza-like illness; Weil’s syndrome, characterized by jaundice, renal failure, hemorrhage and myocarditis with arrhythmias; meningitis or meningoencephalitis; and pulmonary hemorrhage with respiratory failure (https://iris.paho.org/items/23e5a770-23d2-47eb-87c4-e30ac9ff4e13, accessed on 14 January 2026). Reports of infections caused by *Leptospira santarosai* include cases of benign anicteric leptospirosis, Weil’s syndrome, severe pulmonary hemorrhage syndrome (SPHS), and meningitis or meningoencephalitis (neuroleptospirosis), indicating that this species has been associated with all major clinical presentations of leptospirosis [13,14,20,21,22,23,24,25,28,29,32,33,40,41,44,47,51,60,67,68,69,72,79,81,82,83,85,90,96]. Late sequelae of leptospirosis may include chronic fatigue, neuropsychiatric symptoms such as headache, paresis, paralysis, and mood changes and depression, as well as uveitis. *Leptospira santarosai* has been described as a causative agent of leptospirosis with late ocular complications such as uveitis in humans, suggesting the capacity of this species to invade immune-privileged organs [24]. According to the World Health Organization (WHO) and the International Leptospirosis Society (ILS) in “*Human leptospirosis: a guide for diagnosis, surveillance and control”*, laboratory studies of specimens from hospitalized patients frequently show several non-diagnostic abnormalities, including elevated erythrocyte sedimentation rate, thrombocytopenia, leukocytosis, hyperbilirubinemia, increased serum creatinine, elevated creatinine kinase, and elevated serum amylase, and, after crossing host barriers, *Leptospira* disseminates through the bloodstream and can spread to multiple organs. In patients infected with *Leptospira santarosai,* clinical studies have reported alterations in several hematological and inflammatory markers, including increased C-reactive protein (CRP), erythrocyte sedimentation rate (ESR), and procalcitonin levels. These findings indicate a systemic inflammatory response during infection. The kidneys appear to be the main target organ in *Leptospira* infection, as reflected by the frequent renal alterations reported in infected patients, including elevated urea and creatinine levels, proteinuria, hematuria, leukocyturia, dark-colored urine, erythruria, and oliguria. These findings are consistent with renal involvement during infection. Alterations in liver function have also been described, including increased levels of alanine aminotransferase (ALT), aspartate aminotransferase (AST), gamma-glutamil transferasa (GGT), and bilirubin. Elevated bilirubin levels may lead to jaundice, one of the characteristic clinical signs of leptospirosis. Pulmonary complications have also been reported, including pulmonary hemorrhage, pulmonary infiltrates, respiratory failure, alveolar inflammation, and hypercapnia, indicating that infection may involve the respiratory system [11,20,21,22,23,30,31,33,44,50,51,59,69,72,76,79,81,83,89,96]. Overall, the alterations observed in inflammatory markers and organ function tests suggest that *Leptospira santarosai* infection may involve multiple organs, particularly the kidneys, liver, and lungs. However, no single biomarker has been identified as pathognomonic for the disease. Additionally, *Leptospira santarosai* isolates reported in the literature have shown susceptibility to several antibiotics, including azithromycin, doxycycline, ciprofloxacin, penicillin, levofloxacin, minocycline, and ampicillin, and patients generally recover with appropriate treatment [40,83,90]. These findings demonstrate the importance of timely diagnosis and treatment in preventing severe forms of the disease.

Regarding the limitations of this systematic review, the methodological quality of the 84 included studies was evaluated, resulting in 27 articles classified as having high methodological quality (32.14%), 44 as moderate-to-high methodological quality (52.38%), 12 as moderate methodological quality (14.28%), and 1 as low-to-moderate methodological quality (1.19%). One of the main limitations of the evidence included in the systematic review is that only 27 studies were classified as having high methodological quality according to the quality and risk-of-bias assessment. Additional limitations include the use of only five databases for the literature search, the inclusion of articles published exclusively in English and Spanish, the heterogeneity of the study designs, and the inability to perform a meta-analysis because of the heterogeneity of the available data. Regarding serological diversity, although 59 serovars of *Leptospira santarosai* have been identified, this number likely represents only a fraction of the true diversity of the species. Therefore, additional sampling is needed in countries where studies have not yet been conducted. This sampling should focus on detecting and identifying new serovars associated with *Leptospira santarosai* in human, animal, and environmental samples. With respect to geographic distribution, the main limitation is that the available data originate from only 26 countries, representing 13.33% of the world’s countries (26/195). This finding suggests that the current distribution data remain incomplete and that many regions have yet to be investigated. Similarly, *Leptospira santarosai* has been reported in only 24 animal hosts, indicating that its host range may be substantially underestimated. Environmental sources of infection remain among the least explored aspects of the ecology of *Leptospira santarosai*. To date, the species has been reported in wet soils, lakes, waterfalls, and other water sources. Additional environmental studies are therefore needed to better understand its distribution and persistence in natural ecosystems. Regarding human leptospirosis, 24 serovars associated with *Leptospira santarosai* have been reported to infect humans and produce a broad spectrum of clinical manifestations ranging from mild to severe disease. However, further experimental studies are needed to investigate in greater depth the pathophysiology, pathogenicity, virulence factors, and specific clinical manifestations associated with these serovars in humans.

## 5. Conclusions

*Leptospira santarosai* is a pathogenic species characterized by a high serological diversity, a wide geographic distribution, and the ability to infect multiple domestic, synanthropic, and wild animals. Additionally, it can persist in the environment and infect humans, causing clinical manifestations ranging from mild to severe disease, as well as complications such as uveitis, myocarditis, and neuroleptospirosis. The species has been reported on five continents; however, the vast majority of reports originate from the American continent. Therefore, additional epidemiological studies are needed in other regions of the world. These findings highlight the importance of *Leptospira santarosai* as a causative agent of human and animal leptospirosis worldwide and support its inclusion in serological and molecular diagnostic tests to improve diagnostic sensitivity and specificity. Furthermore, this species should be considered in the development of vaccines for humans and animals in regions where it is prevalent. Future studies should examine the ecological determinants that influence the presence, persistence, distribution, and dynamics of *Leptospira santarosai* across different ecosystems. Evolutionary studies based on genome sequencing are also needed to determine the geographic origin, migration patterns, and dispersal routes of the species. Additionally, studies involving different animal hosts and the identification of the associated serovars are necessary to determine whether individual serovars exhibit host specificity or ecological flexibility. Computational biology and bioinformatics should be used to identify pathogenicity and virulence factors that contribute to the pathophysiology of the disease. Comparative genomic analyses with other pathogenic species of the genus *Leptospira* may also help identify the genetic determinants responsible for differences in virulence. Furthermore, additional studies of environmental sources are needed to better understand the ecological behavior of the species and to support the development of more effective prevention and control strategies. Despite recent advances, important knowledge gaps remain. These include the discovery of new serovars, determination of the true serological diversity of the species, identification of its presence in countries where studies have not yet been conducted, evaluation of its ability to survive or multiply in environmental sources, identification of all animal hosts involved in its transmission cycle, and a better understanding of how the species infects, multiplies, and causes disease in humans and animals.

## Figures and Tables

**Table 1:** Evaluation of methodological quality and risk of bias. The table shows the numerical code of the scientific publications included in the systematic review, the first author of each publication, the percentage of compliance with the STROBE guide, the quality level, and the risk of bias of the 84 articles evaluated.

Number	Publication	STROBE Compliance %	Quality Level	Risk of Bias
1	Peláez et al. [22].	86%	High 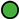	Medium 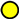
2	Restrepo-Lopez et al. [28].	86%	High 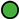	Medium 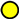
3	Silva-Ramos et al. [29].	86%	High 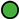	Medium 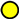
4	Perez-Garcia et al. [30].	86%	High 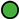	Medium 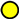
5	Silva-Ramos et al. [31].	86%	High 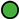	Medium 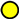
6	Agudelo-Florez et al. [32].	86%	High 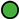	Medium 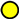
7	Uribe-Restrepo et al. [33].	86%	High 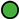	Medium 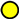
8	Hamond et al. [34].	86%	High 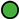	Medium 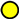
9	Miotto et al. [16].	86%	High 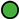	Medium–High 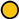
10	Diaz et al. [35].	86%	High 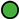	Medium–High 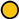
11	Li-Fang et al. [36].	84%	High 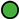	Medium 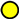
12	Nogueira et al. [37].	84%	High 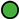	Medium 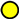
13	Moreno et al. [38].	86%	High 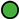	Medium–High 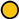
14	Hamond et al. [39].	84%	High 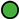	Medium 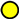
15	Li-Fang et al. [40].	84%	High 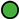	Medium 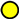
16	Hai Nguyen-Tran et al. [21].	80%	Moderate to High 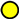	High 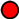
17	Delgado et al. [41].	84%	High 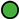	Medium 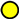
18	Pinto et al. [42].	86%	High 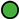	Medium 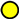
19	Loureiro et al. [43].	86%	High 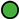	Medium 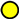
20	Valverde et al. [44].	86%	High 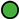	Medium 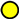
21	Carmon-Gasca et al. [18].	86%	High 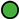	Medium 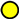
22	Rivera et al. [45].	84%	High 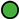	Medium 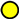
23	Hamond et al. [46].	86%	High 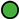	Medium 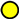
24	Pascal Bourhy et al. [47]	86%	High 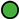	Medium 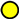
25	Barbosa-Guedes et al. [48].	86%	High 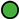	Medium 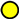
26	Vieira et al. [49].	86%	High 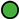	Medium 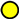
27	Miotto et al. [50].	86%	High 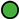	Medium 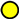
28	Fornazari et al. [17].	86%	High 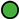	Medium 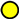
29	Weiss et al. [51].	86%	High 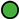	Medium 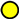
30	Barragan et al. [52].	86%	High 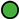	Medium 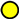
31	Jaeger et al. [53].	86%	High 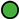	Medium 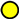
32	Somjit C. et al. [54].	84%	High 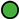	Medium 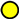
33	Dos santos Madeiros et al. [55].	86%	High 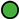	Medium 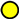
34	Barbosa Guedes et al. [56].	86%	High 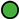	Medium 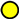
35	Nogueira di Azevedo et al. [57].	86%	High 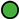	Medium 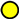
36	Araujo Santos et al. [58].	86%	High 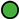	Medium 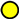
37	Luiza Aymée et al. [59].	86%	High 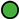	Medium 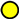
38	Ruzie-Sabljie et al. [60].	86%	High 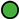	Medium 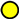
39	Aymée et al. [61].	86%	High 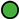	Medium 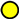
40	Chinchilla et al. [11].	85%	High 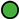	Medium 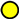
41	De Araujo Santos et al. [62].	86%	High 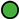	Medium 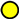
42	Mosquera et al. [63].	86%	High 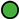	Medium 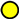
43	Nogueira di Azevedo et al. [64].	86%	High 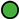	Medium 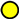
44	Nogueira di Azevedo et al. [65].	86%	High 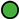	Medium 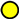
45	Chih-Wei et al. [12].	86%	High 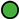	Medium 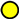
46	Jaeger et al. [66].	85%	High 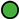	Medium 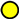
47	Chinchilla et al. [67].	86%	High 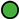	Medium 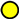
48	Valverde et al. [68].	81%	Moderate to High 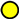	Medium–High 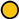
49	Hua-Kung Wang et al. [24].	89%	High 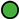	Low–Medium 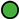
50	Michael R. Wilson et al. [26].	81%	Moderate to High 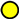	Medium–High 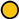
51	Hatem Kallel et al. [69].	81%	Moderate to High 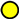	Medium–High 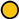
52	Ramos-Vasquez et al. [19]	86%	High 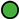	Medium 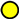
53	Kumari Snehkant Lata et al. [70].	85%	High 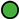	Medium 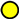
54	Federico S. Kremer et al. [71].	85%	High 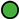	Medium 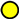
55	Shakila Sudarsshani et al. [72].	89%	High 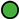	Low–Medium 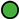
56	Vasconcellos et al. [73].	86%	High 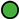	Medium 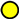
57	Lilenbaum et al. [74].	86%	High 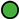	Medium 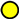
58	Baker et al. [75].	86%	High 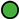	Medium 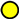
59	Guzman et al. [76].	86%	High 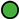	Medium 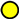
60	Nogueira Di Azevedo et al. [77].	85%	High 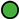	Medium 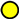
61	Torres-Castro et al. [78].	86%	High 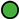	Medium 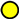
62	Chamidri Naotunna et al. [79].	78%	Moderate 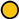	Medium–High 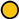
63	Loureiro et al. [80].	86%	High 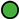	Medium 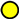
64	Gorman et al. [20].	85%	High 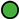	Medium 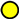
65	Patrick Hochedez et al. [81].	91%	Very High 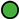	Low–Medium 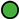
66	Hochedez et al. [82].	91%	Very High 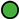	Low–Medium 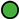
67	Huang-Yu Yang et al. [83].	91%	Very High 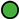	Low–Medium 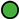
68	Ston et al. [84].	85%	High 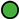	Medium 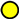
69	Severine Matheus et al. [85].	89%	High 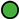	Medium 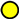
70	Sato et al. [13].	85%	High 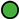	Medium 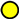
71	Chen-Yi Liao et al. [23]	80%	Moderate to High 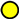	High 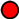
72	Roman-Cardenas et al. [86].	86%	High 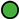	Medium 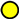
73	Loureiro et al. [87].	86%	High 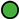	Medium 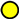
74	Gonzales et al. [25].	80%	Moderate to High 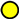	High 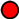
75	Ganosa et al. [88].	85%	High 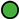	Medium 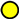
76	Nogueira di azevedo et al. [89].	86%	High 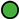	Medium 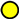
77	Yang et al. [90].	82%	High 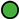	Medium 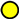
78	Weilin Hu et al. [91].	83%	High 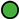	Medium 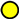
79	Allan et al. [15].	83%	High 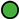	Medium 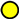
80	De Geus A et al. [92].	86%	High 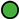	Medium 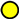
81	De Geus A et al. [93].	86%	High 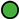	Medium 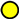
82	Calvopiña et al. [94].	83%	High 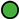	Medium 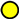
83	Barragan et al. [95].	83%	High 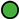	Medium 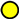
84	Ning Wang et al. [96].	80%	Moderate to High 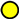	High 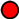

**Table 2:** Serovars associated with *Leptospira santarosai*, including their serogroups, year of isolation, reservoir, country of isolation, author who described the serovar, and the reported reference strain. Dashes indicate unavailable information.

Serogroups	Serovars	Year	Reservoir	Country	Described	Reference Strain
Autumnalis	Alice	1966	Human	Sri Lanka	Chernukha	Alice
Ballum	Perú		Opossum	Perú	-	MW 10
Bataviae	Balboa	1966	Echimyidae -Spiny Rat	Panamá	Sulzer	735 U
	Bataviae	1925	Human	Indonesia	Walch y Soesilo	Schoolby
	Brasiliensis	1961	Opossum	Brazil	Santa Rosa	An 776
	Kobbe	1962	Echimyidae -Spiny Rat	Panamá	Gale	CZ 320
	Rioja	1970	Opossum	Perú	Liceras de Hidalgo	MR 12
Cynopteri	Naparuca		*Galictis cuja*	Perú	-	NN-1
	Tingomaria	1970	Opossum	Perú	Liceras de Hidalgo	M 13
Grippotyphosa	Canalzonae	1964	Echimyidae -Spiny Rat	Panamá	Gale	CZ 188
	Bananal		*Apodemus sylvaticus*	Brazil	-	Aa 14
Hebdomadis	Kremastos	1952	Human	Australia	Wolff y Bohlander	Kremastos
	Abrahamson		Human	Panamá	-	Abrahamson
	Borincana	1951	Human	Puerto Rico	Alexander	HS 622
	Figeiro		Human	Panamá	-	Figeiro
	Goiano	1962	*Bos taurus indicus*	Brazil	Santa Rosa	Bovino 131
	Maru	1962	Water	Panamá	Gale	CZ 285
	Sanmartini	1971	*Sus scrofa domesticus*	Perú	Agirre y Chernukha	CT 63
Javanica	Arenal		Human	Costa Rica		MAVJ 401
	Fluminense	1970	*Apodemus sylvaticus*	Brazil	Cordeiro	Aa 3
	May		Human	Panamá	-	May
	Vargonicas		Rodent	Perú	-	24
Mini	Beye	1960	Echimyidae -Spiny Rat	Panamá	Kmety	1537 U
	Georgia	1952	Procyon	USA	Galton	LT 117
	Ruparupae	1970	Opossum	Perú	Liceras de Hidalgo	M 3
	Szwajizak	1952	Human	Australia	Babudieri	Szwajizak
	Tabaquite	1965	Human	Trinidad and Tobago	Kmety	TRVL 3214
Pomona	Dania		*Bos taurus*	Denmark	-	K 1
	Trópica	1962	Echimyidae -Spiny Rat	Panamá	Gale	CZ 299
Pyrogenes	Alexi	1951	Human	Puerto Rico	Alexander	HS 616
	Bagua		Opossum	Perú	-	MW-12
	Cenepa		Opossum	Perú	-	MW-2
	Costa Rica		Human	Costa Rica	-	INCIENSA 04
	Princestown	1971	Human	Trinidad and Tobago	Green	TRVL 112499
	Pyrogenes	1924	Human	Panamá	-	Northrup
	Varela	1964	Opossum	Nicaragua	Sulzer	1019
Sarmin	Machiguenga	1970	Opossum	Perú	Liceras de Hidalgo	MMD 3
	Rio	1973	*Rattus rattus*	Brazil	Cordeiro	Rr 5
	Weaveri	1961	Human	Panamá	Gale	CZ 390
Sejroe	Caribe	1965	*Rattus norvegicus*	Trinidad and Tobago	Green	TRVL 61866
	Gorgas	1967	Echimyidae -Spiny Rat	Panamá	Sulzer	1413 U
	Guaricura	1962–1968	*Bos taurus indicus*	Brazil	Santa Rosa	Bov. G
	Trinidad	1972	Human	Trinidad and Tobago	Kmety	TRVL 34056
Shermani	Babudieri		*Sus scrofa domesticus*	Perú	-	CI 40
	Luis	1970	Opossum	Perú	Liceras de Hidalgo	M 6
	Shermani	1967	Echimyidae -Spiny Rat	Panamá	Sulzer	1342 K
Tarassovi	Aguatia		*Bos taurus*	Perú	-	45-74
	Atchafalaya		Opossum	USA	Roth	LSU 1013
	Atlantae	1955	Opossum	USA	Wolff y Bohlander	LT 81
	Bakeri	1955	Opossum	USA	Galton	LT 79
	Bravo	1961	Human	Panamá	Gale	Bravo
	Chagres	1967	Echimyidae -Spiny Rat	Panamá	Sulzer	1913 K
	Corredores		Human	Costa Rica	-	JICH 05
	Darien		Opossum	Panamá	Sulzer	637 K
	Gatuni	1967	Opossum	Panamá	Sulzer	1473 K
	Navet	1971	Human	Trinidad and Tobago	Green	TRVL 109873
	Rama	1962	Opossum	Nicaragua	Clark	316
	Sulzerae	1970	Human	China	Zhang Fang-zheng	Mengpeng A 82
Not designated	Wawain *	1999	Opossum	Perú	Brenner	MW6

* Serovar without designated serogroup.

## Data Availability

No new data were created or analyzed in this study. Data sharing is not applicable to this article.

## Supplementary Materials

The following supporting information can be downloaded at: https://www.mdpi.com/article/10.3390/microorganisms14061364/s1. Supplementary Table S1. Search algorithms used in the PubMed, Scopus, Lilacs, Google Scholar, Web of Science, and SciELO databases to identify publications that investigated: serological diversity, geographic distribution, reservoirs, natural sources of infection, human leptospirosis, and genomes of *Leptospira santarosai*. Supplementary Table S2. Summary of the study type of the 84 scientific articles included in the systematic review and the assessment of methodological quality and risk of bias using an appropriate evaluation guide. Supplementary Table S3. Summary of the 84 scientific articles included in the systematic review and the extracted information on serological diversity, geographical distribution, reservoirs, natural sources of infection, and reported cases of human leptospirosis caused by *Leptospira santarosai*. Supplementary Table S4. Summary of geographic distributions of serovars associated with *Leptospira santarosai*. The table lists the countries where the species has been reported, the corresponding serovars, and the total number of serovars identified per country. Supplementary Table S5. Summary of the representative host taxa, environmental sources, and accidental hosts (humans) reported to be infected with serovars of *Leptospira santarosai*. Supplementary Table S6. Summary of clinical presentations and complications described in patients infected with *Leptospira santarosai*. The table summarizes the country, the infecting species or serovar, the reported clinical presentations of the disease, including benign anicteric leptospirosis, Weil’s syndrome, and severe pulmonary hemorrhagic syndrome (SPHS), as well as complications that may occur after infection such as myocarditis, uveitis, and neuroleptospirosis. It also lists the bibliographic sources from which the data were extracted. Supplementary Table S7. Summary of the clinical manifestations and laboratory findings (blood cell count, kidney function, liver function, urine chemistry, lung function, inflammatory markers, and antibiotic response) reported in patients with leptospirosis caused by *Leptospira santarosai*. The table also lists the bibliographic sources from which the information was extracted.
