# Supplementary material for: Leptospira santarosai: A Systematic Review on Its Serological Diversity, Geographical Distribution, Natural Sources of Infection, and Human Leptospirosis
Source: Microorganisms. 2026 Jun 18;14(6):1364. doi: 10.3390/microorganisms14061364 (PMC13304201; doi:10.3390/microorganisms14061364)
Supplement: Supplementary file 1 [file microorganisms-14-01364-s001.zip › PRISMA_2020_abstract_checklist-update.pdf]

PRISMA

PRISMA 2020 for Abstracts Checklist

Section and Topic	Item #	Checklist item	Reported (Yes/No)
**TITLE**			
Title	1	**Identify the report as a systematic review.**<br><br>*Leptospira santarosai: A Systematic Review on Its Serological Diversity, Geographical Distribution, Natural Sources of Infection, and Human Leptospirosis*. (**lines 1-4**)	Yes
**BACKGROUND**			
Objectives	2	**Provide an explicit statement of the main objective(s) or question(s) the review addresses.**<br><br>Accordingly, the objective of this systematic review was to synthesize information on the serological diversity, geographical distribution, natural sources of infection, and human leptospirosis caused by Leptospira santarosai.(**lines 122-125**)	Yes
**METHODS**			
Eligibility criteria	3	**Specify the inclusion and exclusion criteria for the review.**<br><br>**Inclusion criteria**<br>Studies reporting the molecular identification of *Leptospira santarosai* using techniques such as DNA/DNA hybridization and phylogenetic analysis were included. Additionally, studies that performed serological identification at the serogroup or serovar level using techniques such as Microscopic Agglutination Test (MAT), CAAT (Cross Agglutinin Absorption Test), PFGE (Pulsed-Field Gel Electrophoresis), and Multilocus Sequence Typing (MLST) were also included. Studies addressing one or more of the following topics were considered eligible: serological diversity, reservoirs, natural sources of infection, geographic distribution, human leptospirosis and genomes of *L. santarosai*. (lines 146-154)<br><br>**Exclusion criteria**<br>Articles that were withdrawn, unavailable in databases, without full-text access, opinion pieces or editorials, drafts, and preprints were excluded. The selected articles were downloaded in (.pdf) format and stored in a database for later reading. During the full-text review, manuscripts were excluded if they did not mention *Leptospira santarosai*, addressed the topic only marginally, incorrectly identified the species at molecular level, incorrectly identified the serogroup or serovar at serological level, or lacked information on serological diversity, geographic distribution, reservoir diversity, environmental sources, human leptospirosis, or the genome of *Leptospira santarosai*. (**lines 156-164**)	Yes

PRISMA 2020 for Abstracts Checklist

Section and Topic	Item #	Checklist item	Reported (Yes/No)
Information sources	4	**Specify the information sources (e.g. databases, registers) used to identify studies and the date when each was last searched.**<br><br>The literature search ended on 14 January 2026. The selected articles were down-loaded in PDF format and stored in a database for subsequent review. Information related to the four variables analyzed (serological diversity, geographic distribution, environmen-tal sources of infection, and human leptospirosis) was extracted from each article and rec-orded in a database created in Microsoft Word® (Supplementary Tables S2–S7). The ex-tracted information was organized, tabulated, and graphically summarized to facilitate analysis and synthesis of the available evidence. The protocol used for the systematic re-view was registered in the International Prospective Register of Ongoing Systematic Re-views (PROSPERO) under the code CRD420251058799 and is available at the following link:<br>(https://www.crd.york.ac.uk/PROSPERO/view/CRD420251058799, accessed on 14 January 2026).	Yes
Risk of bias	5	**Specify the methods used to assess risk of bias in the included studies.**<br><br>The type of study was determined for the 84 scientific articles selected for the system-atic review. Depending on the study type of each article, an appropriate guide was select-ed for evaluating methodological quality and risk of bias. After applying the guide, each article was classified as high-quality, moderate-quality, or low-quality. Subsequently, the risk of bias for each scientific publication was determined, and the articles were classified as having high risk of bias, medium risk of bias, or low risk of bias. Finally, the strengths and weaknesses of each scientific article were highlighted. The methodological quality and risk of bias of the included studies were evaluated using the STROBE and ARRIVE guide. STROBE guide (Strengthening the Reporting of Observational Studies in Epidemi-ology) provides guidance for reporting observational studies such as cohort, case–control, and cross-sectional designs; and ARRIVE guide (Animal Research: Reporting of In Vivo Experiments) provides guidelines for reporting research involving animal experiments. The detailed procedure for the quality assessment process and risk of bias is described in detail in the (Supplementary Table S2).	Yes
Synthesis of results	6	**Specify the methods used to present and synthesise results.**<br>The studies that met the eligibility criteria were included in the systematic review. Information related to the six variables analyzed (serological diversity, geographic distribution, reservoirs, environmental sources of infection, human leptospirosis, and genomic diversity) was extracted from each article and recorded in a database created in Microsoft Word. The extracted information was organized, tabulated, and graphically summarized to facilitate analysis and synthesis of the available evidence. (**Lines 167-173**)	Yes
**RESULTS**			
Included studies	7	**Give the total number of included studies and participants and summarise relevant characteristics of**	Yes

Section and Topic	Item #	Checklist item	Reported (Yes/No)
		**studies.**<br><br>Finally, 84 articles met the inclusion criteria and were included in the qualitative synthesis (**Figure 1.**).	
Synthesis of results	8	**Present results for main outcomes, preferably indicating the number of included studies and participants for each.**<br><br>**3.1 Search and selection of information.**<br><br>The search and selection process identified 2,989 records containing the terms "Leptospira santarosai" or "L. santarosai" (PubMed = 30, Scopus = 84, LILACS = 107, Google Scholar = 2,700, Web of Science = 61, SciELO = 7). During the identification stage, records unrelated to the topic (n = 2,646) and duplicate records (n = 241) were removed, leaving 102 records for screening. In the screening stage, the titles and abstracts of these records were evaluated, and five records were excluded (n = 5), leaving 97 records sought for retrieval. Of these, five reports could not be retrieved (n = 5), leaving 92 reports assessed for eligibility. During the eligibility stage, eight articles were excluded due to species without molecular identification (n = 2), serovars without serological identification (n = 2), and incorrect identification of species or serovars (n = 4). Finally, 84 articles met the inclusion criteria and were included in the qualitative synthesis (**Figure 1**).<br><br>The included studies were classified according to their purpose as descriptive or analytical, according to temporality as cross-sectional, according to control of factors as experimental or observational, and according to chronology as retrospective or prospective. The methodological quality of the 84 studies was evaluated, resulting in 27 articles classified as high methodological quality, 44 as moderate-to-high methodological quality, 12 as moderate methodological quality, and 1 as low-to-moderate methodological quality (**Supplementary Table S2**). The information on serological diversity, geographical distribution, reservoirs, natural sources of infection, human leptospirosis and genomes was extracted from each of the 84 articles (**Supplementary Table S3**).<br><br>**3.2 *Leptospira santarosai* history**<br><br>Historically, *Leptospira santarosai* was first isolated from the kidney of a spiny rat captured in the Panama Canal zone and was proposed as a new species in 1987 by Yasuda et al. This species is named in honor of the Brazilian scientist Carlos Santarosa, a veterinary microbiologist and pioneer in leptospirosis research in Brazil [27].	Yes

Section and Topic	Item #	Checklist item	Reported (Yes/No)
		**3.3 Serological diversity of *Leptospira santarosai***<br><br>The first specific objective of this systematic review was to describe serological diversity of *Leptospira santarosai*. According to the information available in the Medical Reference Center for Leptospirosis (https://leptospira.amsterdamumc.org/filters/species/l-santarosai/), this species is associated with 45 serovars and grouped into 14 serogroups.<br><br>However, based on the list described by Brenner *et al*. in 1999 and recent publications, 14 additional serovars have been reported, resulting in a total of 59 serovars distributed across 14 serogroups associated with *Leptospira santarosai* [13, 15-17, 28-35]. As a result of this systematic review, the identified serovars were systematically organized into 14 serogroups comprising 59 serovars associated with *Leptospira santarosai*, providing a synthesis of the current knowledge on the extensive serological diversity of this species (**Table 1** and **Figure 2**).<br><br>**3.4 Geographical distribution of *Leptospira santarosai***<br>As a second objective, the geographic distribution of *Leptospira santarosai* was described, revealing its presence on five continents (excluding Antarctica) [12–14,51,79]. The continent with the highest number of reports of *Leptospira santarosai* was America, followed by Asia, Europe, Oceanica, and Africa. In America, the species has been reported in Panama, Peru, Brazil, Trinidad and Tobago, Colombia, USA, Costa Rica, Puerto Rico, Nicaragua, French West Indies, Mexico, Ecuador, Belize, French Guiana, and Martinique. In Asia, it has been reported in Indonesia, Taiwan, Sri Lanka, Thailand, and India. In Europe, the species has been reported in Denmark, Slovenia, and the United Kingdom. In Oceania, it has been reported in Australia and Palau, while, in Africa, it has been reported in Kenya. The geographic distribution of serovars associated with *Leptospira santarosai* showed that 59 serovars have been identified across 26 countries and territories. However, in seven countries, isolates were identified only at the species level. Panama reported the highest number of serovars (18), followed by Peru (13) and Brazil (7) (Figure 3 and Supplementary Table S4).<br><br>**3.5 Animals infected by *Leptospira santarosai***	

Section and Topic	Item #	Checklist item	Reported (Yes/No)
		The third objective was to relate Leptospira santarosai serovars to their animal hosts. A total of 45 serovars were reported infecting 24 hosts, including the following: Galictis cuja, pigs (Sus scrofa domesticus) [52], rodents (Rodentia) [52], raccoons (Procyon lotor) [13], cattle (Bos taurus) [59], opossums (Didelphimorphia) [13], capybaras (Hydrochoerus hydrochaeris) [38], buffalo (Bubalus bubalis) [73], dogs (Canis lupus familiaris) [30,31,50,76,89], goats (Capra aegagrus hircus) [74], coatis (Nasua) [16], coyotes (Canis latrans) [75], equids (Equidae) [18], sheep (Ovis aries) [62], Marmosops ocellatus [49,55], Marmosops paulensis [49,55], Metachirus myosuros [49,55], Metachirus nudicaudatus [49,55], Monodelphis glirina [49,55], Monodelphis peruviana [49,55], nine-banded armadillos (Dasypus novemcinctus) [16], Nectomys squamipes [52], wild boar (Sus scrofa) [35], and bats [78]. Among these hosts, opossums (Didelphimorphia) were associated with the highest number of serovars (16), followed by rodents (13) and cattle (7). Additionally, 13 isolates were identified only at the species level (Figure 4 and Supplementary Table S5).<br><br>**3.6 *Leptospira santarosai* and natural sources of infection**<br>The fourth specific objective of the systematic review was to evaluate the association of *Leptospira santarosai* with environmental sources. The species has been reported in wet soils, lakes, and waterfalls. However, only one environmental isolate has been identified at the serovar level (*Leptospira santarosai* serovar Maru), which was detected in water sources in the Panama Canal area (Figure 4 and Supplementary Table S5) [12,19,45,54,82,84,88,94].<br><br>**3.7 *Leptospira santarosai* and human leptospirosis**<br>The fifth objective was to identify *Leptospira santarosai* as a causative agent of human leptospirosis and to describe the clinical manifestations associated with infection. This species has been reported as an infectious agent in humans, with 24 isolates identified at the serovar level (Kremastos, Abrahamson, Borincana, Figeiro, Arenal, May, Szwajizak, Tabaquite, Alexi, Alice, Costa Rica, Princestown, Pyrogenes, Trinidad, Navet, Sulzerae, Bataviae, Weaveri, Corredores, Bravo, Canalzonae, Shermani, Beye, and Babudieri) [13,14,20–25,28,29,32,33,40,41,44,47,51,60,67–69,72,79,81–83,85,90,96]. The infection has been associated with clinical presentations such as benign anicteric leptospirosis, Weil's syndrome, and severe pulmonary hemorrhagic syndrome (SPHS) [21,22]. Additionally, complications include myocarditis [23], uveitis [24], and	

PRISMA

PRISMA 2020 for Abstracts Checklist

Section and Topic	Item #	Checklist item	Reported (Yes/No)
		neuroleptospirosis [25,96] have been reported (Figure 5 and Supplementary Table S6). Laboratory findings included urinary abnormalities such as proteinuria, hematuria, leukocyturia, dark-colored urine, leukocyturia, erythruria, and oliguria. Renal alterations include increased urea and creatinine levels. Hematological findings include elevated C-reactive protein (CRP), erythrocyte sedimentation rate (ESR), and procalcitonin levels. Hepatic involvement has been associated with increased liver enzyme levels, including alanine aminotransferase (ALT), aspartate aminotransferase (AST), gamma-glutamil transferasa (GGT), and bilirubin. Pulmonary alterations such as pulmonary hemorrhage, pulmonary infiltrates, respiratory failure, alveolar inflammation, and hypercapnia have also been described. However, no single clinical sign has been pathognomonic for the disease [11,20–23,30,31,33,44,50,51,59,69,72,76,79,81,83,89,96]. Additionally, *Leptospira santarosai* has been reported to be susceptible to several antibiotics, including azithromycin, doxycycline, ciprofloxacin, penicillin, levofloxacin, minocycline, and ampicillin, resulting in rapid recovery of the patients [40,83,90] (Figure 5 and Supplementary Table S7).	
**DISCUSSION**			
Limitations of evidence	9	**Provide a brief summary of the limitations of the evidence included in the review (e.g. study risk of bias, inconsistency and imprecision).**<br><br>Regarding the limitations of this systematic review, the methodological quality of the 84 included studies was evaluated, resulting in 27 articles classified as having high methodological quality (32.14%), 44 as moderate-to-high methodological quality (52.38%), 12 as moderate methodological quality (14.28%), and 1 as low-to-moderate methodological quality (1.19%). One of the main limitations of the evidence included in the systematic re-view is that only 27 studies were classified as having high methodological quality ac-cording to the quality and risk-of-bias assessment. Additional limitations include the use of only five databases for the literature search, the inclusion of articles published exclu-sively in English and Spanish, the heterogeneity of the study designs, and the inability to perform a meta-analysis because of the heterogeneity of the available data. **(Page 19)**	Yes
Interpretation	10	**Provide a general interpretation of the results and important implications.**	Yes

Section and Topic	Item #	Checklist item	Reported (Yes/No)
		**4. Discussion**<br><br>According to the results of this systematic review, Leptospira santarosai is a pathogenic species that exhibits wide serological diversity, with at least 59 serovars grouped into 14 serogroups. This broad intraspecific serological diversity underscores the epidemiological importance of this species and likely reflects its capacity to adapt to a wide range of envi-ronments. Indeed, Leptospira santarosai has been reported in at least 24 animal hosts, envi-ronmental sources such as water bodies, lakes, waterfalls, and wet soils, and humans, for whom 24 infective serovars have been described. Understanding the serological diversity of Leptospira santarosai is crucial and presents several challenges in public and veterinary health. From a diagnostic perspective, serological tests, such as the Microscopic Aggluti-nation Test (MAT) depend on including local serovars to be accurate. If the serovars pre-sent in a region are unknown, false-negative results may occur. Epidemiologically, identi-fying circulating serovars allows for a better understanding of transmission patterns, an-imal reservoirs, and environmental risk factors. Regarding prevention and control, vac-cines against Leptospira generate serovar-specific immunity only against the serovar in-cluded in the vaccine formulation. Therefore, knowledge of serological diversity is essen-tial for the development of effective vaccines and control strategies adapted to local condi-tions. Additionally, the broad serological diversity of Leptospira santarosai has important public health implications. Different serovars can persist in the environmental for pro-longed periods and be transmitted through water, wet soils, or animal hosts, increasing the risk of outbreaks. From an evolutionary perspective, the presence of numerous serovars likely reflects a long history of adaptation to diverse environments and hosts, resulting in the diversification of the genetic content and, consequently, the serological phenotype of the species. These findings also highlight important knowledge gaps regarding the eco-logical determinants of Leptospira santarosai. Future studies should investigate the envi-ronmental factors that influence the presence, distribution, and dynamics of this species across different ecosystems. According to the list of serovars reported by Brenner et al. in 1999 [14], the pathogenic species with the highest number of serovars in the genus Lepto-spira are L.eptospira interrogans (91 serovars), L.eptospira santarosai (59 serovars), L. borgpetersenii (49 serovars), L. kirschneri (39 serovars), L. noguchii (18 serovars), and L. weilii (15 serovars). Therefore, Leptospira santarosai is the second most serologically diverse	

Section and Topic	Item #	Checklist item	Reported (Yes/No)
		spe-cies currently recognized within the genus and is consequently of considerable epidemio-logical, environmental, and clinical importance.<br><br>Leptospira santarosai has a wide geographic distribution and has been reported on five continents and 26 countries [12–14,51,70,79,91–93]. Reports of this species are most fre-quent in the American continent, particularly in Central America, South America, and the Caribbean islands. Panama reported the highest number of serovars (18), followed by Peru (13) and Brazil (7). However, the greater number of serovars reported in these countries likely reflects a higher sampling effort and a larger number of studies rather than the true serological diversity of the species. Therefore, it is necessary to carry out further research in countries where information is currently unavailable to determine the actual serological diversity and distribution of Leptospira santarosai. Additionally, in Taiwan, Leptospira san-tarosai serovar Shermani has been reported as one of the most prevalent serovars associat-ed with human infection. These observations suggest that this species represents an im-portant etiological agent of leptospirosis in various regions of the world [12]. Furthermore, 45 serovars were reported exclusively in a single country, whereas eight serovars were de-tected in more than one country: Canalzonae (Panama and Colombia), Beye (Panama and Colombia), Pyrogenes (Panama and Indonesia), Babudieri (Peru and Colombia), Tabaquite (Trinidad and Tobago, and the French Wes Indies), Alice (Sri Lanka and Colombia), Shermani (Panama and Taiwan), and Princestown (Trinidad and Tobago, and the USA). These findings indicate that, while many Leptospira santarosai serovars appear to have re-stricted geographic distributions, some are detected across multiple countries, showing a pattern of dispersion among the countries. When comparing geographical regions, the Americas reported the highest number of countries with the presence of Leptospira santaro-sai (15 countries and 57 serovars), followed by Asia (5 countries and 6 serovars), Europe (3 countries and 1 serovar), Oceania (2 countries and 2 serovars), and Africa (1 country, without identification of serovars). Regarding possible distribution routes, dispersion patterns can be observed among countries that share the same serovars. One group in-cludes Panama, Colombia, Peru, Sri Lanka and Indonesia, whereas another includes Trinidad and Tobago, the United States, and the French West Indies. However, clonal dis-persal studies among the serovars are required to verify these proposed distribution patterns. It is also important to acknowledge the potential for selection bias, as many countries lack taxonomic studies capable of identifying Leptospira santarosai and its serovars. Consequently, the	

Section and Topic	Item #	Checklist item	Reported (Yes/No)
		current distribution patterns may reflect differences in re-search efforts and taxonomic resolution rather than the true geographic distribution of the species.<br><br>The evidence indicates that 39 serovars of Leptospira santarosai infect 24 hosts [13,16,18,30,31,34,35,38,46,48–50,52,53,55–59,61–66,73–77,80,86,87,89]. This serological diversity suggests that the species is associated with multiple hosts. Several serovars were reported infecting the same animal species (which is consistent with the theory that some serovars selectively infect certain animal hosts), while other serovars were identified in-fecting several animal hosts. For example, the serovar Georgia has been reported in Procy-on lotor, whereas the serovar Carioca has been reported in Capra aegagrus hircus. In contrast, several serovars have been detected in multiple animal species, including Sanmartini in pigs and cattle, Babudieri in dogs and pigs, Bananal in capybaras and rodents, Beye in rodents and dogs, and Guaricura in cattle, buffalo, and dogs. These observations demon-strate that certain serovars may circulate among multiple host species, suggesting the pos-sibility of interspecies transmission. The presence of Leptospira santarosai in agriculturally important hosts such as cattle, buffalo, goats, and pigs highlight the potential role of live-stock in the epidemiology of leptospirosis caused by this species. These observations sug-gest that animal hosts other than rodents may contribute to the transmission cycle of the disease. Furthermore, it is evident that agricultural activities may also represent a risk fac-tor for leptospirosis caused by this species, given its high capacity to infect various agri-culturally important hosts such as cattle, buffalo, goats, and pigs. These observations suggest that livestock may contribute to transmission in both rural and per-urban settings. Reports of infection in domestic animals such as dogs indicate that these animals may al-so represent potential sources of exposure for humans. In addition, infections detected in wild animals suggest the existence of a sylvatic cycle that may represent another potential source of human infection when people enter these habitats. Several serovars have been reported in both animals and humans, including Canalzonae (rodents and humans), Babudieri (pigs, canines, and humans), Shermani (rodents and humans), Beye (rodents, canines, and humans). This overlap suggests that these hosts may act as a potential risk factor for human infection. Overall, these findings indicate that Leptospira santarosai may circulate across wildlife, livestock, and domestic animal populations. Human activities such as agriculture and	

Section and Topic	Item #	Checklist item	Reported (Yes/No)
		environmental disturbance may increase contact with infected hosts, thereby increasing the risk of infection.<br><br>Furthermore, at least seven studies have reported the presence of Leptospira santarosai in various environmental sources such as soils, lakes, and waterfalls [12,19,45,54,84,88,94]. However, only one study identified the isolates at serovar level (Leptospira santarosai serovar Maru in water sources from the Panama Canal Zone). This finding demonstrates the ability of this species to persist in the environment. These obser-vations highlight the need for additional studies detecting the presence of pathogenic Lep-tospira species in environmental sources to determine their role in the environmental per-sistence of the bacteria, evaluate whether environmental replication occurs, and determine whether water and soil act as effective vehicles for disease transmission. Moreover, the de-tection of Leptospira santarosai in water sources could be associated with the infection in non-traditional exposure contexts, such as environmental disasters, recreational water ac-tivities, and aquatic events. These scenarios have previously been identified as risk factors for infection by other Leptospira species [36,37,39,71,95,97–100]. The limited number of re-ports of Leptospira santarosai from environmental sources likely reflects both the small number of studies conducted and the methodological challenges associated with the de-tection and isolation of Leptospira from environmental samples. These challenges include the complexity of environmental matrices, which contain DNA from multiple organisms; the presence of substances that inhibit molecular assays, such as humic compounds, heavy metals, and organic matter; the low concentration of bacteria in environmental samples, resulting in reduced detection rates; and the high microbial diversity present in these environments, which requires the use of highly specific molecular markers. Addi-tional limitations include the presence of degraded or fragmented DNA, which can lead to false-negative results, and the lack of standardized protocols for DNA extraction from different environmental matrices. Furthermore, the detection of bacterial DNA does not necessarily indicate the presence of viable or metabolically active organisms, which may lead to inaccurate conclusions regarding environmental transmission risks.<br><br>At least 24 serovars belonging to Leptospira santarosai have been identified as causa-tive agents of human leptospirosis. The wide variety of serovars reported in human infec-tions demonstrates the presence of multiple animal reservoirs and environmental	

Section and Topic	Item #	Checklist item	Reported (Yes/No)
		sources contaminated with the bacterium, which may act as sources of infection for humans. Ac-cording to the World Health Organization (WHO) and the International Leptospirosis So-ciety (ILS), the disease presents in four broad clinical categories: a mild influenza-like ill-ness; Weil's syndrome, characterized by jaundice, renal failure, hemorrhage and myocar-ditis with arrhythmias; meningitis or meningoencephalitis; and pulmonary hemorrhage with respiratory failure (https://iris.paho.org/items/23e5a770-23d2-47eb-87c4-e30ac9ff4e13, accessed on 14 Janu-ary 2026). Reports of infections caused by Leptospira santarosai include cases of benign anicteric leptospirosis, Weil's syndrome, severe pulmonary hemorrhage syndrome (SPHS), and meningitis or meningoencephalitis (neuroleptospirosis), indicating that this species has been associated with all major clinical presentations of leptospirosis [13,14,20–25,28,29,32,33,40,41,44,47,51,60,67–69,72,79,81–83,85,90,96]. Late sequelae of leptospiro-sis may include chronic fatigue, neuropsychiatric symptoms such as headache, paresis, paralysis, and mood changes and depression, as well as uveitis. Leptospira santarosai has been described as a causative agent of leptospirosis with late ocular complications such as uveitis in humans, suggesting the capacity of this species to invade immune-privileged organs [24]. According to the World Health Organization (WHO) and the International Leptospirosis Society (ILS) in "Human leptospirosis: a guide for diagnosis, surveillance and control", laboratory studies of specimens from hospitalized patients frequently show sev-eral non-diagnostic abnormalities, including elevated erythrocyte sedimentation rate, thrombocytopenia, leukocytosis, hyperbilirubinemia, increased serum creatinine, elevated creatinine kinase, and elevated serum amylase, and, after crossing host barriers, Leptospira disseminates through the bloodstream and can spread to multiple organs. In patients in-fected with Leptospira santarosai, clinical studies have reported alterations in several he-matological and inflammatory markers, including increased C-reactive protein (CRP), erythrocyte sedimentation rate (ESR), and procalcitonin levels. These findings indicate a systemic inflammatory response during infection. The kidneys appear to be the main tar-get organ in Leptospira infection, as reflected by the frequent renal alterations reported in infected patients, including elevated urea and creatinine levels, proteinuria, hematuria, leukocyturia, dark-colored urine, erythruria, and oliguria. These findings are consistent with renal involvement during infection. Alterations in liver function have also been de-scribed, including increased levels of alanine aminotransferase (ALT), aspartate ami-notransferase (AST), gamma-glutamil transferasa (GGT), and bilirubin. Elevated bilirubin levels may lead to jaundice, one of	

Section and Topic	Item #	Checklist item	Reported (Yes/No)
		the characteristic clinical signs of leptospirosis. Pul-monary complications have also been reported, including pulmonary hemorrhage, pul-monary infiltrates, respiratory failure, alveolar inflammation, and hypercapnia, indicating that infection may involve the respiratory system [11,20–23,30,31,33,44,50,51,59,69,72,76,79,81,83,89,96]. Overall, the alterations observed in in-flammatory markers and organ function tests suggest that Leptospira santarosai infection may involve multiple organs, particularly the kidneys, liver, and lungs. However, no sin-gle biomarker has been identified as pathognomonic for the disease. Additionally, Lepto-spira santarosai isolates reported in the literature have shown susceptibility to several anti-biotics, including azithromycin, doxycycline, ciprofloxacin, penicillin, levofloxacin, minocycline, and ampicillin, and patients generally recover with appropriate treatment [40,83,90]. These findings demonstrate the importance of timely diagnosis and treatment in preventing severe forms of the disease.<br><br>Regarding the limitations of this systematic review, the methodological quality of the 84 included studies was evaluated, resulting in 27 articles classified as having high methodological quality (32.14%), 44 as moderate-to-high methodological quality (52.38%), 12 as moderate methodological quality (14.28%), and 1 as low-to-moderate methodological quality (1.19%). One of the main limitations of the evidence included in the systematic re-view is that only 27 studies were classified as having high methodological quality ac-cording to the quality and risk-of-bias assessment. Additional limitations include the use of only five databases for the literature search, the inclusion of articles published exclu-sively in English and Spanish, the heterogeneity of the study designs, and the inability to perform a meta-analysis because of the heterogeneity of the available data. Regarding se-rological diversity, although 59 serovars of Leptospira santarosai have been identified, this number likely represents only a fraction of the true diversity of the species. Therefore, ad-ditional sampling is needed in countries where studies have not yet been conducted. This sampling should focus on detecting and identifying new serovars associated with Lepto-spira santarosai in human, animal, and environmental samples. With respect to geographic distribution, the main limitation is that the available data originate from only 26 countries, representing 13.33% of the world's countries (26/195). This finding suggests that the cur-rent distribution data remain incomplete and that many regions have yet to be investigat-ed. Similarly, Leptospira santarosai has been reported in only 24 animal hosts, indicating that its host range may be substantially underestimated.	

Section and Topic	Item #	Checklist item	Reported (Yes/No)
		Environmental sources of infec-tion remain among the least explored aspects of the ecology of Leptospira santarosai. To date, the species has been reported in wet soils, lakes, waterfalls, and other water sources. Ad-ditional environmental studies are therefore needed to better understand its distribution and persistence in natural ecosystems. Regarding human leptospirosis, 24 serovars asso-ciated with Leptospira santarosai have been reported to infect humans and produce a broad spectrum of clinical manifestations ranging from mild to severe disease. However, further experimental studies are needed to investigate in greater depth the pathophysiology, pathogenicity, virulence factors, and specific clinical manifestations associated with these serovars in humans.<br><br>**Conclusions**<br><br>*Leptospira santarosai* is a pathogenic species characterized by a high serological diversity, a wide geographic distribution, and the ability to infect multiple domestic, synanthropic, and wild animals. Additionally, it can persist in the environment and infect humans, causing clinical manifestations ranging from mild to severe disease, as well as complications such as uveitis, myocarditis, and neuroleptospirosis. The species has been reported on five continents; however, the vast majority of reports originate from the American continent. Therefore, additional epidemiological studies are needed in other regions of the world. These findings highlight the importance of *Leptospira santarosai* as a causative agent of human and animal leptospirosis worldwide and support its inclusion in serological and molecular diagnostic tests to improve diagnostic sensitivity and specificity. Furthermore, this species should be considered in the development of vaccines for humans and animals in regions where it is prevalent. Future studies should examine the ecological determinants that influence the presence, persistence, distribution, and dynamics of *Leptospira santarosai* across different ecosystems. Evolutionary studies based on genome sequencing are also needed to determine the geographic origin, migration patterns, and dispersal routes of the species. Additionally, studies involving different animal hosts and the identification of the associated serovars are necessary to determine whether individual serovars exhibit host specificity or ecological flexibility. Computational biology and bioinformatics should be used to identify pathogenicity and virulence factors that contribute to the pathophysiology of the disease. Comparative genomic analyses with other pathogenic species of the genus *Leptospira* may also help identify the genetic determinants responsible for differences in virulence. Furthermore, additional studies of	

PRISMA

PRISMA 2020 for Abstracts Checklist

Section and Topic	Item #	Checklist item	Reported (Yes/No)
		environmental sources are needed to better understand the ecological behavior of the species and to support the development of more effective prevention and control strategies. Despite recent advances, important knowledge gaps remain. These include the discovery of new serovars, determination of the true serological diversity of the species, identification of its presence in countries where studies have not yet been conducted, evaluation of its ability to survive or multiply in environmental sources, identification of all animal hosts involved in its transmission cycle, and a better understanding of how the species infects, multiplies, and causes disease in humans and animals.	
**OTHER**			
Funding	11	**Specify the primary source of funding for the review.**<br><br>This project was funded by the Universidad de la Sabana. Grant number MED-20-2024, under the Project "Detección de genes relacionados con la Resistencia antibiótica en Leptospira".	Yes
Registration	12	**Provide the register name and registration number.**<br><br>The protocol used for writing the systematic review was registered in the International Prospective Register of Ongoing Systematic Reviews (PROSPERO) under the code CRD420251058799 and is available at the following link: https://www.crd.york.ac.uk/PROSPERO/view/CRD420251058799<br><br>**(Page 4)**	Yes

From: Page MJ, McKenzie JE, Bossuyt PM, Boutron I, Hoffmann TC, Mulrow CD, et al. The PRISMA 2020 statement: an updated guideline for reporting systematic reviews. BMJ 2021;372:n71. doi: 10.1136/bmj.n71. This work is licensed under CC BY 4.0. To view a copy of this license, visit https://creativecommons.org/licenses/by/4.0/
