# Supplementary material for: Leptospira santarosai: A Systematic Review on Its Serological Diversity, Geographical Distribution, Natural Sources of Infection, and Human Leptospirosis
Source: Microorganisms. 2026 Jun 18;14(6):1364. doi: 10.3390/microorganisms14061364 (PMC13304201; doi:10.3390/microorganisms14061364)
Supplement: Supplementary file 1 [file microorganisms-14-01364-s001.zip › microorganisms-4232833-supplementary.pdf]

Supplementary Table S1. Search algorithms used in the PubMed, Scopus, Lilacs, Google Scholar, Web of Science, and SciELO databases to identify publications that investigated: serological diversity, geographic distribution, reservoirs, natural sources of infection, human leptospirosis, and genomes of *Leptospira santarosai*.

DataBase	Search algorithm	Publications number
PubMed	(*Leptospira santarosai*) OR (*L. santarosai*)	30
Scopus	(ALL (*Leptospira santarosai*) OR TITLE-ABS-KEY (*L. santarosai*))	84
LILACS	(tw:(*Leptospira santarosai*)) OR (tw:(*L. santarosai*))	107
Google Scholar	*Leptospira santarosai* OR *L. santarosai*	2700
Web of Science	*Leptospira santarosai* OR *L. santarosai*	61
SciELO	*Leptospira santarosai* [Todos los indices] or *L. santarosai* [Todos los indices]	7

Supplementary Table S2. Summary of the study type of the 84 scientific articles included in the systematic review and the assessment of methodological quality and risk of bias using an appropriate evaluation guide.

#	Publication	Study type	Bias and quality assessment tools
1	**Peláez *et al*.** [22]. Genetic diversity of *Leptospira* in northwestern Colombia: first report of *Leptospira santarosai* as a recognized leptospirosis agent.	observational, descriptive and laboratory study	STROBE
2	**Restrepo-Lopez *et al*.** [28]. Malaria, Dengue Fever, and Leptospirosis in the Urabá Antioqueño Region, Colombia: Etiological and Molecular Characterization among Patients with Acute Undifferentiated Febrile Illness.	descriptive cross-sectional observational study with a molecular characterization component	STROBE
3	**Silva-Ramos *et al*.** [29]. Molecular Characterization of *Leptospira* Species among Patients with Acute Undifferentiated Febrile Illness from the Municipality of Villeta, Colombia.	descriptive cross-sectional observational study with molecular characterization	STROBE
4	**Perez-Garcia *et al*.** [30]. Canine Leptospirosis in a Northwestern Region of Colombia: Serological, Molecular and Epidemiological Factors.	descriptive cross-sectional observational study with serological, molecular and epidemiological analysis	STROBE
5	**Silva-Ramos *et al*.** [31]. Molecular Evidence of *Leptospira* spp. Infection Among Household Dogs From 15 Municipalities of the Department of Caldas, Colombia.	descriptive cross-sectional observational study with molecular and epidemiological characterization	STROBE
6	**Agudelo-Florez *et al*.** [32]. Genotipificación y evaluación de la dinámica de infección de un aislamiento colombiano de *Leptospira santarosai* en el modelo experimental en hámster.	Preclinical experimental study in an animal model (hamster), with molecular characterization and evaluation of infection dynamics	ARRIVE
7	**Uribe-Restrepo *et al*.** [33]. Clinical presentation of human leptospirosis in febrile patients: Urabá, Colombia.	descriptive cross-sectional observational study with clinical and diagnostic characterization of patients	STROBE

8	**Hamond *et al*.** [34]. *Leptospira borgpetersenii* serovar Hardjo and *Leptospira santarosai* serogroup Pyrogenes isolated from bovine dairy herds in Puerto Rico.	Cross-sectional observational study with experimental laboratory component (isolation and microbiological characterization), oriented towards epidemiological surveillance in cattle.	STROBE
9	**Miotto *et al*.** [16]. Molecular and serological characterization of the first *Leptospira santarosai* strain isolated from a dog.	Experimental laboratory study, descriptive and cross-sectional, oriented to the molecular and serological characterization of a strain isolated in a dog.	STROBE
10	**Diaz *et al*.** [35]. First detection of *Leptospira santarosai* in the reproductive track of a boar: A potential threat to swine production and public health.	Descriptive observational study of case report type in a boar, with experimental laboratory component (molecular and serological), oriented to epidemiological surveillance and public health.	STROBE
11	**Li-Fang *et al*.** [36]. Sequence of *Leptospira santarosai* serovar Shermani genome and prediction of virulence-associated genes.	experimental laboratory study, descriptive and exploratory, based on genomic sequencing and bioinformatics analysis of a bacterial strain.	STROBE
12	**Nogueira *et al*.** [37]. Draft-genome sequences of *Leptospira santarosai* strains isolated from urogenital tract of cows.	Experimental laboratory study, descriptive and exploration, based on genomic sequencing of strains isolated from the urogenital tract of cows.	STROBE
13	**Moreno *et al*.** [38]. Characterization of *Leptospira santarosai* Serogroup Grippotyphosa Serovar Bananal Isolated from Capybara (*Hydrochaeris hydrochaeris*) in Brazil.	Descriptive observational study of the case report type in wildlife, with an experimental laboratory component (isolation and microbiological/serological characterization).	STROBE

14	**Hamond *et al.*** [39]. A multilocus variable number tandem repeat analysis assay provides high discrimination for genotyping *Leptospira santarosai* strains.	Methodological experimental study of the validation of a molecular technique (MLVA) for genotyping of bacterial strains.	STROBE
15	**Li-Fang *et al.*** [40]. Potential impact on kidney infection: a whole-genome analysis of *Leptospira santarosai* serovar Shermani.	Descriptive and exploratory experimental study of complete genomic analysis, aimed at identifying genes potentially associated with kidney infection.	STROBE
16	**Hai Nguyen-Tran *et al.*** [21]. Use of Advanced Diagnostics for Timely Identification of Travel-associated *Leptospira santarosai* Infection in Four Adolescents Through Plasma Microbial Cell-free DNA Sequencing With the Karius Test.	Descriptive observational study of the clinical case series type, with a methodological component of diagnostic validation by sequencing of cell-free DNA in plasma (Karius Test).	STROBE
17	**Delgado *et al.*** [41]. New Genetic Variants of *Leptospira* spp Characterized by MLST from Peruvian Isolates.	Descriptive experimental study of molecular characterization using MLST, aimed at identifying new genetic variants of *Leptospira* spp. in Peruvian isolates.	STROBE
18	**Pinto *et al.*** [42]. Plurality of *Leptospira* strains on slaughtered animals suggest a broader concept of adaptability of leptospires to cattle.	Cross-sectional descriptive observational study in cattle, with microbiological and molecular analysis of *Leptospira* strains to evaluate bacterial diversity and adaptation.	STROBE
19	**Loureiro *et al.*** [43]. Molecular analysis of leptospires from serogroup Sejroe obtained from asymptomatic cattle in Rio de Janeiro Brazil reveals genetic proximity to serovar Guaricura.	Cross-sectional descriptive observational study in asymptomatic cattle, with microbiological and molecular analysis of *Leptospira* strains of the Sejroe serogroup, aimed at evaluating their genetic proximity to the Guaricura serovar.	STROBE
20	**Valverde *et al.*** [44]. New serovars of *Leptospira* isolated from patients in Costa Rica: implications for public health	Cross-sectional descriptive observational study in humans, with microbiological and	STROBE

		molecular analysis of *Leptospira* isolates, aimed at identifying new serovars and discussing their epidemiological and public health implications.	
21	**Carmon-Gasca *et al*.** [18]. Detection of *Leptospira santarosai* and *L. kirschneri* in cattle: new isolates with potential impact in bovine production and public health.	Cross-sectional descriptive observational study in cattle, with microbiological and molecular analysis of *Leptospira santarosai* and *L. kirschneri* isolates, aimed at evaluating their potential impact on animal production and public health.	STROBE
22	**Rivera *et al*.** [45]. Diversidad genética de aislamientos peruanos de *Leptospira* spp. mediante electroforesis en gel de campo pulsado.	Cross-sectional descriptive observational study, with microbiological and molecular analysis by electrophoresis, aimed at evaluating the genetic diversity of Peruvian isolates of *Leptospira*	STROBE
23	**Hamond *et al*.** [46]. Genotyping of *Leptospira* directly in urine samples of cattle demonstrates a diversity of species and strains in Brazil.	Cross-sectional descriptive observational study in cattle, with microbiological and molecular analysis by direct genotyping in urine, aimed at evaluating the diversity of species and strains of *Leptospira* in Brazil.	STROBE
24	**Pascal Bourhy *et al*.** [47]. Serovar Diversity of Pathogenic *Leptospira* Circulating in the French West Indies.	Cross-sectional descriptive observational study, with microbiological and molecular analysis of *Leptospira* isolates, aimed at evaluating the diversity of circulating pathogenic serovars in the French Antilles and their epidemiological implications.	STROBE

25	**Barbosa-Guedes *et al*.** [48]. Circulating *Leptospira* species identified in cattle of the Brazilian Amazon.	Cross-sectional descriptive observational study in cattle, with microbiological and molecular analysis of *Leptospira* isolates, aimed at evaluating the diversity of circulating species in the Brazilian Amazon and their epidemiological and productive implications.	STROBE
26	**Vieira *et al*.** [49]. Pathogenic *Leptospira* species are widely disseminated among small mammals in Atlantic Forest biome.	Cross-sectional descriptive observational study in small wild mammals, with microbiological and molecular analysis, aimed at evaluating the diversity and dissemination of pathogenic *Leptospira* species in the Atlantic Forest biome and their epidemiological implications.	STROBE
27	**Miotto *et al*.** [50]. Prospective study of canine leptospirosis in shelter and stray dog populations: Identification of chronic carriers and different *Leptospira* species infecting dogs.	Prospective observational cohort study in shelter and stray dogs, with microbiological and molecular analysis, aimed at identifying chronic carriers and characterizing the *Leptospira* species that infect canines.	STROBE
28	**Fornazari *et al*.** [17]. *Leptospira* reservoirs among wildlife in Brazil: Beyond rodents.	Cross-sectional descriptive observational study in Brazilian wildlife, with microbiological and molecular analysis, aimed at identifying *Leptospira* reservoirs beyond rodents and evaluating their epidemiological implications.	STROBE
29	**Weiss *et al*.** [51]. An Extended Multilocus Sequence Typing (MLST) Scheme for Rapid Direct Typing of *Leptospira* from Clinical Samples.	Methodological and experimental study of diagnostic validation, aimed at the development of an expanded MLST scheme	STROBE

		for rapid typing of *Leptospira* directly in clinical samples.	
30	**Barragan *et al*.** [52]. High *Leptospira* Diversity in Animals and Humans Complicates the Search for Common Reservoirs of Human Disease in Rural Ecuador.	Cross-sectional descriptive observational study, with microbiological and molecular analysis, with an eco-epidemiological approach, aimed at characterizing the diversity of *Leptospira* in humans and animals in a rural environment of Ecuador and at evaluating the difficulty of identifying common reservoirs of the disease.	STROBE
31	**Jaeger *et al*.** [53]. Novel MLST sequence types of pathogenic *Leptospira* spp.: Opening the black box of animal leptospirosis in Brazil.	Methodological and experimental laboratory study, with a cross-sectional descriptive approach and molecular analysis, aimed at identifying new types of MLST sequences in pathogenic strains of *Leptospira* isolated from animals in Brazil, to better understand the genetic diversity and epidemiology of animal leptospirosis.	STROBE
32	**Somjit C. *et al*.** [54]. Potentially Pathogenic *Leptospira* in the Environment of an Elephant Camp in Thailand.	Cross-sectional descriptive observational study, with microbiological and molecular analysis, aimed at characterizing the presence of potentially pathogenic *Leptospira* in the environment of an elephant camp in Thailand and evaluating	STROBE

		its epidemiological importance as a source of risk for animals and humans.	
33	**Dos santos Madeiros *et al*.** [55]. Small Mammals as Carriers/Hosts of *Leptospira* spp. in the Western Amazon Forest.	Cross-sectional descriptive observational study, with microbiological and molecular analysis, aimed at characterizing the role of small mammals as carriers of *Leptospira* spp. in the western Amazon and evaluating their epidemiological importance as reservoirs of the disease.	STROBE
34	**Barbosa Guedes *et al*.** [56]. *Leptospira* strains isolated from cattle in the Amazon region, Brazil, evidence of a variety of species and serogroups with a high frequency of the Sejroe serogroup.	Cross-sectional descriptive observational study, with microbiological and molecular analysis, aimed at characterizing the diversity of *Leptospira* strains isolated from cattle in the Brazilian Amazon and at evaluating the frequency of serogroups, especially Sejroe, in the regional epidemiological context.	STROBE
35	**Nogueira di Azevedo *et al*.** [57]. Characterization of leptospiral DNA in the follicular fluid of non-pregnant cows.	Cross-sectional descriptive observational study, with microbiological and molecular analysis, of an exploratory nature, aimed at characterizing the presence of leptospiral DNA in the follicular fluid of non-pregnant cows and evaluating its possible epidemiological and reproductive relevance.	STROBE
36	**Araujo Santos *et al*.** [58]. New insights on *Leptospira* sp. infection in ewes maintained in field semiarid conditions.	Cross-sectional descriptive observational study, with microbiological and molecular analysis, of an exploratory nature, aimed at characterizing *Leptospira* infection in sheep	STROBE

		kept in semi-arid conditions and evaluating its epidemiological importance in sheep production.	
37	**Luiza Aymée *et al*.** [59]. *Leptospira* spp. strains associated with Bovine Genital Leptospirosis (BGL).	Cross-sectional descriptive observational study, with microbiological and molecular analysis, of an exploratory nature, aimed at characterizing the *Leptospira* strains associated with bovine genital leptospirosis and evaluating their epidemiological relevance in cattle reproduction.	STROBE
38	**Ruzie-Sabljie *et al*.** [60]. First Report on *Leptospira* Species Isolated from Patients in Slovenia.	This is a descriptive cross-sectional observational study, with microbiological and molecular analysis, of an exploratory nature, aimed at characterizing the *Leptospira* species isolated from human patients in Slovenia and providing unprecedented epidemiological evidence in that country.	STROBE
39	**Aymée *et al*.** [61]. The role of *Leptospira santarosai* serovar Guaricura as agent of Bovine Genital Leptospirosis.	Cross-sectional descriptive observational study, with microbiological and molecular analysis, aimed at confirming the role of *Leptospira santarosai* serovar Guaricura as an agent of bovine genital leptospirosis and providing relevant epidemiological evidence for cattle reproduction.	STROBE
40	**Chinchilla *et al*.** [11]. Phylogenomic of *Leptospira santarosai*, a prevalent pathogenic species in the Americas.	A cross-sectional descriptive methodological study, based on phylogenomic and comparative analysis of	STROBE

		Leptospira santarosai strains, aimed at characterizing their genetic diversity and providing evidence on their role as a prevalent pathogenic species in the Americas.	
41	**De Araujo Santos *et al*.** [62]. Follow-up investigation revealed that sheep may play an important role in the transmission of *Leptospira* spp. infection in Caatinga biome field conditions.	Longitudinal observational study of follow-up, descriptive and epidemiological, with microbiological and molecular analysis, aimed at evaluating the role of sheep as reservoirs of *Leptospira* in field conditions of the Caatinga biome.	STROBE
42	**Mosquera *et al*.** [63]. Mixed *Leptospira* infections in domestic animals from a rural community with high leptospirosis endemicity.	Cross-sectional descriptive observational study, with microbiological and molecular analysis, aimed at characterizing mixed *Leptospira* infections in domestic animals of an endemic rural community, providing relevant epidemiological evidence for animal and public health.	STROBE
43	**Nogueira di Azevedo *et al*.** [64]. Genetic Diversity and Clonal Expansion of Pathogenic *Leptospira* in Brazil: A Multi-Host and Multi-Regional Panorama	Cross-sectional descriptive observational study, of a molecular epidemiological, multi-host and multi-regional nature, aimed at characterizing the genetic diversity and clonal expansion of pathogenic *Leptospira* in Brazil.	STROBE
44	**Nogueira di Azevedo *et al*.** [65]. Ecological range and host–biome associations of pathogenic *Leptospira* in Brazil: A One Health perspective from a tropical area.	Cross-sectional descriptive observational study, of an ecological and molecular epidemiological nature, multi-host and multi-regional, aimed at characterizing the	STROBE

		associations between hosts, biomes and the distribution of pathogenic *Leptospira* in Brazil under a One Health approach.	
45	**Chih-Wei *et al*.** [12]. Leptospirosis in Taiwan – an underestimated infectious disease.	A retrospective, descriptive, observational study of a national epidemiological nature, aimed at highlighting the burden of leptospirosis in Taiwan and its underestimation as a public health problem	STROBE
46	**Jaeger *et al*.** [66]. VNTR analysis demonstrates new patterns and high genetic diversity of *Leptospira* sp. of animal origin in Brazil.	Cross-sectional descriptive observational study, of a microbiological and molecular nature, aimed at characterizing the genetic diversity of *Leptospira* of animal origin in Brazil through VNTR analysis, with epidemiological implications in the veterinary and public health context.	STROBE
47	**Chinchilla *et al*.** [67]. In-house isolation protocol from human serum samples demonstrates the circulating of a broad diversity of *Leptospira* serogroups in Costa Rica.	Methodological and observational cross-sectional descriptive study, of a microbiological and epidemiological nature in humans, aimed at validating an isolation protocol and characterizing the diversity of *Leptospira* serogroups circulating in Costa Rica.	STROBE
48	**Valverde *et al*.** [68]. Arenal, a new *Leptospira* serovar of serogroup Javanica, isolated from a patient in Costa Rica.	Descriptive observational study of a clinical case, with a microbiological and taxonomic approach, aimed at isolating and characterizing a new serovar of *Leptospira* in humans in Costa Rica.	STROBE

49	**Hua-Kung Wang *et al*.** [24]. Factor associated with severity and mortality in patients with confirmed leptospirosis at a regional hospital in northern Taiwan.	Retrospective analytical observational study, hospital-based in humans, aimed at identifying factors associated with the severity and mortality of leptospirosis in a regional hospital in Taiwan.	STROBE
50	**Michael R. Wilson *et al*.** [26]. Actionable Diagnosis of Neuroleptospirosis by Next-Generation Sequencing.	Descriptive observational clinical case study, with a methodological and diagnostic focus, demonstrating the application of NGS to identify *Leptospira* in a patient with neuroleptospirosis.	STROBE
51	**Hatem Kallel *et al*.** [69]. First report of human *Leptospira santarosai* infection in French Guiana.	Descriptive observational clinical case study, with a microbiological and diagnostic focus, that reports for the first time the human infection by *Leptospira santarosai* in French Guiana.	STROBE
52	**Ramos-Vasquez *et al*.** [19]. Isolation and molecular identification of *Leptospira santarosai* and *Leptospira interrogans* in equines from eastern Mexico.	Cross-sectional descriptive observational study, of a microbiological and molecular nature, aimed at isolating and identifying *Leptospira* species in horses in Mexico, with veterinary epidemiological and public health relevance.	STROBE
53	**Kumari Snehkant Lata *et al*.** [70]. Whole genome sequencing and de novo assembly of three virulent Indian isolated of *Leptospira*.	Experimental laboratory study, with a genomic and molecular approach, oriented towards the sequencing and de novo assembly of virulent *Leptospira* isolates in India, with relevance to taxonomic and epidemiological research.	STROBE

54	**Federico S. Kremer et al.** [71]. Draft Genome Sequences of *Leptospira santarosai* Strains U160, U164, and U233, Isolated from Asymptomatic Cattle.	Experimental genomic and molecular study, of an exploratory descriptive nature, oriented to the preliminary sequencing of *Leptospira santarosai* strains isolated in asymptomatic cattle, with relevance for veterinary and epidemiological research.	STROBE
55	**Shakila Sudarsshani et al.** [72]. Leptospirosis: A Potential Culprit for Chronic Kidney Disease of Uncertain Etiology.	This is a narrative review study with an epidemiological and exploratory approach, aimed at generating hypotheses about the relationship between leptospirosis and chronic kidney disease of uncertain etiology.	STROBE
56	**Vasconcellos et al.** [73]. Isolation of *Leptospira santarosai*, serovar guaricura from buffaloes (*Bubalus bubalis*) in Vale do Ribeira, São Paulo, Brazil.	Cross-sectional descriptive observational study, with a microbiological and veterinary focus, oriented towards the isolation and characterization of *Leptospira santarosai* serovar guaricura in buffaloes in Brazil, with epidemiological relevance for animal and public health.	STROBE
57	**Lilenbaum et al.** [74]. Molecular characterization of the first leptospires isolated from goats in Brazil.	Cross-sectional descriptive observational study, with a microbiological and molecular approach, focused on the isolation and characterization of *Leptospira* in goats in Brazil, with veterinary and public health epidemiological relevance.	STROBE
58	**Baker et al.** [75]. Survey of Coyotes (Canis latrans) for Vector-Borne and Bacterial Pathogens in South Carolina and Tennessee, USA.	Cross-sectional descriptive observational study, with an epidemiological and microbiological approach, focused on the detection of pathogens in coyotes in South	STROBE

		Carolina and Tennessee, with relevance to public health and the ecology of zoonotic diseases.	
59	**Guzman et al.** [76]. Domestic dogs in indigenous Amazonian communities: key players in *Leptospira* cycling and transmission?	Cross-sectional descriptive observational study, with a veterinary and zoonotic epidemiological focus, supported by microbiological/molecular analyses, aimed at exploring the role of domestic dogs in Amazonian communities as reservoirs and transmitters of *Leptospira*	STROBE
60	**Nogueira Di Azevedo et al.** [77]. Comparative genomics of *Leptospira santarosai* reveal genomic adaptations in bovine genital strains.	Experimental laboratory study with a focus on comparative genomics and bioinformatics analysis, aimed at describing genomic adaptations of *Leptospira santarosai* in strains isolated from cattle, with implications for epidemiology and pathogenesis.	STROBE
61	**Torres-Castro et al.** [78]. *Leptospira* patógena en murciélagos de Campeche y Yucatán, México.	Cross-sectional descriptive observational study, with an epidemiological and microbiological approach, aimed at detecting pathogenic *Leptospira* in bats in Campeche and Yucatán, with relevance to public health and the ecology of zoonotic transmission.	STROBE
62	**Chamidri Naotunna et al.** [79]. Etiological agents causing leptospirosis in Sri Lanka: A review.	Narrative review of the scientific literature on the etiological agents of leptospirosis in Sri Lanka, with an epidemiological and microbiological focus, aimed at synthesizing	STROBE

		existing knowledge and highlighting local adaptations of the disease.	
63	**Loureiro *et al*.** [80]. Usage of a selective media (EMJH-STAFF) in primary culturing of pathogenic leptospires from bovine clinical samples.	Experimental laboratory study, with a methodological and microbiological approach, aimed at validating the use of a selective medium for the primary isolation of *Leptospira* in bovine clinical samples.	STROBE
64	**Gorman *et al*.** [20]. *Leptospira* enrichment culture followed by ONT metagenomic sequencing allows better detection of *Leptospira* presence and diversity in water and soil samples.	Methodological experimental laboratory study, with a microbiological and genomic approach, aimed at validating a metagenomic enrichment and sequencing strategy to improve the detection and characterization of *Leptospira* in environmental water and soil samples.	STROBE
65	**Patrick Hochedez *et al*.** [81]. Factors Associated with Severe Leptospirosis, Martinique, 2010–2013.	Retrospective analytical observational study of hospital cohorts, with a clinical-epidemiological approach, aimed at identifying factors associated with severe leptospirosis in Martinique between 2010 and 2013.	STROBE
66	**Hochedez *et al*.** [82]. Outbreak of leptospirosis among canyoning participants, Martinique, 2011.	Descriptive observational study of an epidemic outbreak, with a clinical-epidemiological and public health approach, aimed at characterizing the cases of leptospirosis that occurred among canyoning participants in Martinique in 2011.	STROBE

67	**Huang-Yu Yang *et al*.** [83]. Overlooked Risk for Chronic Kidney Disease after Leptospiral Infection: A Population-Based Survey and Epidemiological Cohort Evidence.	Mixed observational study (cross-sectional population survey and epidemiological cohort), with a clinical-epidemiological approach, aimed at evaluating the association between *Leptospira* infection and the risk of chronic kidney disease.	STROBE
68	**Ston *et al*.** [84]. Diverse lineages of pathogenic *Leptospira* species are widespread in the environment in Puerto Rico, USA.	Cross-sectional descriptive observational study, with an environmental and microbiological-genomic focus, aimed at characterizing the diversity of lineages of pathogenic *Leptospira* in water and soil of Puerto Rico.	STROBE
69	**Severine Matheus *et al*.** [85]. Microbiological investigations of severe tropical infections in French Amazonia: a prospective pilot study of first-line tests and metagenomics.	Prospective pilot, observational clinical-epidemiological study with methodological and microbiological-genomic component, aimed at evaluating the usefulness of first-line tests and metagenomics in the diagnosis of severe tropical infections in French Guiana.	STROBE
70	**Sato *et al*.** [13]. A systematic survey of environmental DNA in Palau's lakes and waterfalls reveals an increase in *Leptospira* levels after flooding.	Cross-sectional descriptive observational study, with an environmental and molecular focus, aimed at characterizing the presence and variation of *Leptospira* using eDNA in lakes and waterfalls in Palau, and at evaluating the impact of flooding on their levels.	STROBE

71	**Chen-Yi Liao *et al*.** [23]. Acute Respiratory Distress Syndrome Manifested by Leptospirosis Successfully Treated by Extracorporeal Membrane Oxygenation (ECMO).	Descriptive clinical study, case report type, aimed at showing the successful application of ECMO in the management of leptospirosis complicated by ARDS.	STROBE
72	**Roman-Cardenas *et al*.** [86]. Identificación molecular de *Leptospira* spp., presente en el ganado lechero del cantón Loja- Ecuador.	Cross-sectional descriptive observational study with an epidemiological and microbiological-molecular approach, aimed at identifying the presence of *Leptospira* spp. in dairy cattle in Loja, Ecuador.	STROBE
73	**Loureiro *et al*.** [87]. High frequency of leptospiral vaginal carriers among slaughtered cows.	Cross-sectional descriptive observational study with an epidemiological and microbiological-molecular approach, aimed at estimating the frequency of vaginal carriers of *Leptospira* in slaughtered cows.	STROBE
74	**Gonzales *et al*.** [25]. Detection of Leptospirosis Genome from the Aqueous Humor of a Patient with Bilateral Uveitis.	Descriptive clinical study, case report type, with a microbiological-molecular component, aimed at showing the detection of the *Leptospira* genome in the aqueous humor of a patient with bilateral uveitis.	STROBE
75	**Ganosa *et al*.** [88]. Determining risk for severe leptospirosis by molecular analysis of environmental surface waters for pathogenic *Leptospira*.	Cross-sectional descriptive observational study with an environmental and molecular focus, aimed at evaluating the risk of severe leptospirosis by detecting pathogenic *Leptospira* in surface waters.	STROBE
76	**Nogueira di azevedo *et al*.** [89]. Molecular Epidemiology of Pathogenic *Leptospira* spp. Infecting Dogs in Latin America.	A descriptive, cross-sectional, multicenter, observational study with a molecular-epidemiological approach, aimed at	STROBE

		genetically characterizing the *Leptospira* strains that infect dogs in Latin America.	
77	**Yang *et al*.** [90]. Leptospirosis renal disease: understanding the initiation by Toll-like receptors.	Experimental laboratory study, with an immunological and molecular approach, aimed at elucidating the pathogenic mechanisms of renal leptospirosis through the role of Toll-like receptors.	STROBE
78	**Weilin Hu *et al*.** [91]. *Leptospira* and leptospirosis in China.	Epidemiological and microbiological narrative review on leptospirosis in China, aimed at contextualizing the disease in terms of distribution, agents and clinical relevance.	STROBE
79	**Allan *et al*.** [15]. Epidemiology of Leptospirosis in Africa: A Systematic Review of a Neglected Zoonosis and a Paradigm for 'One Health' in Africa.	A systematic epidemiological review with a "One Health" approach, aimed at characterizing the situation of leptospirosis in Africa and highlighting the need for integrated approaches between human, animal and environmental health.	STROBE
80	**De Geus A *et al*.** [92]. Clinical leptospirosis in Kenya (2): A field study in Nyanza Province.	A descriptive cross-sectional field observational study, with a clinical-epidemiological approach, conducted in patients from Nyanza Province (Kenya) to characterize leptospirosis under local conditions.	STROBE
81	**De Geus A *et al*.** [93]. Clinical leptospirosis in Kenya (1): a clinical study in Kwale District, Coast Province.	A descriptive cross-sectional field observational study, with a clinical-epidemiological approach, carried out in patients from Kwale District (Kenya Coast)	STROBE

		to characterize leptospirosis under local conditions.	
82	**Calvopiña *et al*.** [94]. Leptospirosis in Ecuador: Current Status and Future Prospects.	A narrative epidemiological and public health review on leptospirosis in Ecuador, describing the current state of the disease and proposing future perspectives for its control.	STROBE
83	**Barragan *et al*.** [95]. Draft Genome Sequence of the First Pathogenic *Leptospira* Isolates from Ecuador.	Experimental genomic-descriptive study, focused on the sequencing and molecular characterization of the first pathogenic strains of *Leptospira* isolated in Ecuador.	STROBE
84	**Ning Wang *et al*.** [96]. Atypical leptospirosis: an overlooked cause of aseptic meningitis.	Descriptive clinical observational case series study, focused on documenting the atypical presentation of leptospirosis as a cause of aseptic meningitis.	STROBE

QUALITY ASSESSMENT OF THE PUBLICATIONS

STROBE (*STRENGTHENING THE REPORTING OF OBSERVATIONAL STUDIES IN EPIDEMIOLOGY*)

STROBE Evaluation Methodology

1. **Identification of study type**

The evaluator determines whether the article corresponds to an observational study (cohort, case-control, cross-sectional). If not, the non-applicable items are marked accordingly.

2. **Review of the 23 STROBE items**

The evaluator examines each item in the checklist (title, abstract, background, objectives, design, participants, variables, bias, results, discussion, funding, ethics).

3. **Assignment of compliance**

Each item is classified as:
Yes: when it is clearly reported.
Partial: when it is reported incompletely or implicitly.
N/A: when it does not apply to the study design.

4. **Scoring**

The evaluator assigns 1 point to items marked as Yes, 0.5 points to items marked as Partial, and excludes N/A items from the denominator.

5. **Calculation of compliance percentage**

The evaluator calculates the compliance percentage using the formula:

$$\text{Compliance \%} = \frac{\text{Yes} + (0.5 \times \text{Partial})}{\text{Applicable items}} \times 100$$

6. **Classification of overall quality**

Based on the percentage obtained, the evaluator classifies the article as:
High quality (80–100%)
Moderate quality (50–79%)
Low quality (0–49%)

7. **Narrative synthesis**

The evaluator complements the score with a qualitative analysis of strengths (clarity of objectives, robust methodology, well-presented results) and weaknesses (bias not discussed, absence of sample size calculation, lack of ethical or funding information).

8. **Final judgment**

Finally, the evaluator issues an overall judgment on the article's quality and risk of bias, integrating both the compliance percentage and the narrative synthesis.

RISK OF BIAS ASSESSMENT METHODOLOGY (STROBE-based)

Identify critical STROBE items

The evaluator focuses on items most relevant to bias:

- Bias (item 9) → Are potential biases identified and discussed?
- Participants (items 6 and 13) → Is participant selection clearly described?
- Variables (item 7) → Are variables defined and measured reliably?
- Study size (item 10) → Is sample size justified?
- Statistical methods (item 12) → Are methods appropriate and well reported?

Evaluate limitations

- The evaluator checks whether the article acknowledges its limitations (item 19) and assesses how these affect internal and external validity.

Assign risk of bias level

Based on the number and severity of deficiencies:

Low risk of bias → Most critical items are well reported and discussed.

Medium risk of bias → Some critical items are incomplete or weakly discussed, but the study retains reasonable validity.

High risk of bias → Several critical items are missing or poorly reported; results are unreliable or difficult to generalize.

Integrate with STROBE compliance percentage
The evaluator combines the compliance percentage with the qualitative bias assessment to provide a final judgment.

Risk of Bias Level	Criteria	Examples
**Low risk of bias**	Most critical STROBE items are clearly reported and discussed.	Biases identified and managed; participant selection well described; variables defined and measured with valid methods; sample size justified; statistical methods appropriate.
**Medium risk of bias**	Some critical items are incomplete or poorly discussed, but the study retains reasonable validity.	Biases mentioned but not quantified; unclear inclusion criteria; insufficient sample size but results still interpretable; limited statistical analysis.
**High risk of bias**	Several critical items are missing or poorly reported; results are unreliable or difficult to generalize.	Biases not discussed; participant selection unclear or not described; variables poorly defined; no sample size justification; inappropriate or absent statistical methods.

Publication 1:

Peláez *et al.* Genetic diversity of *Leptospira* in northwestern Colombia: first report of *Leptospira santarosai* as a recognized leptospirosis agent.

STROBE Item	Compliance	Observation
Title/Abstract	Yes	Clear, informative, mentions genetic diversity and new agent.
Background/Rationale	Yes	Provides epidemiological context in Antioquia, Colombia.
Objectives	Yes	Explicit aim: identify species/serovars and report L. santarosai.
Study Design	Yes	Laboratory-based genetic diversity study described.
Setting	Yes	Antioquia, Colombia; isolates from humans and environment.
Participants	Partial	Human/animal isolates described, but selection criteria limited.
Variables	Yes	Species and serovar identification defined via MLST, PFGE, monoclonal antibodies.
Data Sources/Measurement	Yes	Molecular and serological methods clearly explained.
Bias	Partial	Potential biases in isolate selection not fully discussed.
Study Size	Partial	25 isolates analyzed; justification of sample size not provided.
Quantitative Variables	Yes	Genetic markers and serovar typing reported.
Statistical Methods	Partial	Phylogenetic analysis described, but limited statistical detail.
Participants (Results)	Yes	Numbers of isolates per species/serovar reported.
Descriptive Data	Yes	Clear breakdown of isolates (L. santarosai, L. interrogans, L. meyeri).
Outcome Data	Yes	Identification of new etiologic agents reported.
Main Results	Yes	L. santarosai recognized as human pathogen in Colombia.
Other Analyses	Partial	Limited additional analyses beyond phylogenetics.
Key Results (Discussion)	Yes	Findings summarized clearly.
Limitations	Partial	Limitations of sample size and representativeness not deeply elaborated.
Interpretation	Yes	Results interpreted in context of regional epidemiology.
Generalizability	Partial	Implications for Colombia discussed, but external generalizability limited.
Funding	Yes	Funding sources acknowledged.
Ethical Considerations	Yes	Ethical approval implied for human/animal isolates.

Overall Assessment

- **STROBE Compliance %:** 86%
- **Quality Level:** High
- **Risk of Bias:** Medium (due to limited discussion of bias, sample size justification, and generalizability).
- **Strengths:** Clear objectives, robust molecular methods, novel identification of L. santarosai as a human pathogen.

- **Weaknesses:** Small sample size, limited statistical detail, incomplete discussion of biases and limitations.

Publication 2:

Malaria, Dengue Fever, and Leptospirosis in the Urabá Antioqueño Region, Colombia: Etiological and Molecular Characterization among Patients with Acute Undifferentiated Febrile Illness.

STROBE Item	Compliance	Observation
Title/Abstract	Yes	Clear, informative, specifies diseases and molecular characterization.
Background/Rationale	Yes	Provides epidemiological context of AUFI in Urabá Antioqueño.
Objectives	Yes	Explicit aim: characterize etiologies and molecular diversity.
Study Design	Yes	Active surveillance study described.
Setting	Yes	Two hospitals in Urabá Antioqueño, Colombia.
Participants	Yes	184 febrile patients enrolled voluntarily; inclusion criteria described.
Variables	Yes	Pathogens identified (Plasmodium spp., DENV serotypes/genotypes, Leptospira spp.).
Data Sources/Measurement	Yes	Direct, serological, molecular, and rapid diagnostic methods explained.
Bias	Partial	Potential selection bias acknowledged but not deeply analyzed.
Study Size	Partial	184 patients; justification of sample size not fully discussed.
Quantitative Variables	Yes	Pathogen prevalence and genotypes reported.
Statistical Methods	Partial	Phylogenetic analysis described; limited statistical detail beyond molecular typing.
Participants (Results)	Yes	Numbers and distribution of etiologies reported.
Descriptive Data	Yes	Clear breakdown of malaria, dengue, and leptospirosis cases.
Outcome Data	Yes	Identification of species, serotypes, and genotypes.
Main Results	Yes	Malaria most frequent; DENV genotypes and Leptospira species identified.
Other Analyses	Partial	Limited additional analyses beyond phylogenetics.
Key Results (Discussion)	Yes	Findings summarized clearly.
Limitations	Partial	Limitations of sample size and representativeness mentioned but not deeply elaborated.
Interpretation	Yes	Results interpreted in context of regional epidemiology.
Generalizability	Partial	Implications for Urabá discussed; external generalizability limited.
Funding	Yes	Funding sources acknowledged.
Ethical Considerations	Yes	Ethical approval obtained for patient enrollment.

Overall Assessment

- **STROBE Compliance %:** 86%
- **Quality Level:** High

- **Risk of Bias:** Medium (due to limited discussion of bias, sample size justification, and generalizability).
- **Strengths:** Clear objectives, robust molecular methods, comprehensive pathogen characterization, relevant to public health.
- **Weaknesses:** Sample size justification limited, incomplete bias analysis, restricted generalizability beyond Urabá region.

Publication 3:

Molecular Characterization of *Leptospira* Species among Patients with Acute Undifferentiated Febrile Illness from the Municipality of Villeta, Colombia.

STROBE Item	Compliance	Observation
Title/Abstract	Yes	Clear, informative, specifies molecular characterization in Villeta.
Background/Rationale	Yes	Provides epidemiological context of leptospirosis in Colombia.
Objectives	Yes	Explicit aim: identify circulating Leptospira species among febrile patients.
Study Design	Yes	Active surveillance, molecular characterization study described.
Setting	Yes	Municipality of Villeta, Colombia; hospital-based febrile patients.
Participants	Yes	56 febrile patients enrolled; inclusion criteria described.
Variables	Yes	Pathogen identification via qPCR and sequencing of multiple genes.
Data Sources/Measurement	Yes	Molecular methods (qPCR, conventional PCR, sequencing) explained.
Bias	Partial	Potential selection bias acknowledged but not deeply analyzed.
Study Size	Partial	56 patients; justification of sample size not fully discussed.
Quantitative Variables	Yes	Pathogen prevalence and genetic diversity reported.
Statistical Methods	Partial	Phylogenetic analysis described; limited statistical detail beyond molecular typing.
Participants (Results)	Yes	Numbers and distribution of positive cases reported.
Descriptive Data	Yes	Clear breakdown of Leptospira species identified.
Outcome Data	Yes	Identification of L. santarosai and other pathogenic species.
Main Results	Yes	25% of patients positive; pathogenic clade identified.
Other Analyses	Partial	Limited additional analyses beyond phylogenetics.
Key Results (Discussion)	Yes	Findings summarized clearly.
Limitations	Partial	Limitations of sample size and representativeness mentioned but not deeply elaborated.
Interpretation	Yes	Results interpreted in context of regional epidemiology.
Generalizability	Partial	Implications for Villeta discussed; external generalizability limited.

Funding	Yes	Funding sources acknowledged.
Ethical Considerations	Yes	Ethical approval obtained for patient enrollment.

Overall Assessment

- **STROBE Compliance %:** 86%
- **Quality Level:** High
- **Risk of Bias:** Medium (due to limited discussion of bias, sample size justification, and generalizability).
- **Strengths:** Clear objectives, robust molecular methods, identification of pathogenic Leptospira species in Villeta.
- **Weaknesses:** Small sample size, incomplete bias analysis, restricted generalizability beyond Villeta region.

Publication 4:

Canine Leptospirosis in a Northwestern Region of Colombia: Serological, Molecular and Epidemiological Factors.

STROBE Item	Compliance	Observation
Title/Abstract	Yes	Clear, informative, specifies canine leptospirosis and factors studied.
Background/Rationale	Yes	Provides epidemiological context of leptospirosis in Colombia.
Objectives	Yes	Explicit aim: characterize serological, molecular, and epidemiological factors in dogs.
Study Design	Yes	Cross-sectional study described.
Setting	Yes	Northwestern Colombia; veterinary clinics and field sampling.
Participants	Yes	105 dogs enrolled; inclusion criteria described.
Variables	Yes	Serovars, molecular markers, and epidemiological risk factors defined.
Data Sources/Measurement	Yes	Serological (MAT), molecular (PCR), and epidemiological surveys explained.
Bias	Partial	Potential selection bias acknowledged but not deeply analyzed.
Study Size	Partial	105 dogs; justification of sample size not fully discussed.
Quantitative Variables	Yes	Seroprevalence and molecular detection rates reported.
Statistical Methods	Partial	Logistic regression used for risk factors; limited detail on statistical assumptions.
Participants (Results)	Yes	Numbers and distribution of positive dogs reported.
Descriptive Data	Yes	Clear breakdown of serovars and molecular findings.
Outcome Data	Yes	Identification of pathogenic Leptospira species and associated risk factors.
Main Results	Yes	Seroprevalence ~30%; molecular detection confirmed pathogenic species.
Other Analyses	Partial	Limited additional analyses beyond logistic regression.

Key Results (Discussion)	Yes	Findings summarized clearly.
Limitations	Partial	Limitations of sample size and representativeness mentioned but not deeply elaborated.
Interpretation	Yes	Results interpreted in context of canine and zoonotic epidemiology.
Generalizability	Partial	Implications for Colombia discussed; external generalizability limited.
Funding	Yes	Funding sources acknowledged.
Ethical Considerations	Yes	Ethical approval obtained for animal research.

Overall Assessment

- **STROBE Compliance %:** 86%
- **Quality Level:** High
- **Risk of Bias:** Medium (due to limited discussion of bias, sample size justification, and generalizability).
- **Strengths:** Clear objectives, robust serological and molecular methods, identification of risk factors relevant to canine and zoonotic transmission.
- **Weaknesses:** Small sample size, incomplete bias analysis, restricted generalizability beyond the study region.

Publication 5:

Molecular Evidence of *Leptospira* spp. Infection Among Household Dogs From 15 Municipalities of the Department of Caldas, Colombia.

STROBE Item	Compliance	Observation
Title/Abstract	Yes	Clear, informative, specifies canine leptospirosis in Caldas.
Background/Rationale	Yes	Provides epidemiological context of leptospirosis in Colombia.
Objectives	Yes	Explicit aim: detect molecular evidence of Leptospira spp. in household dogs.
Study Design	Yes	Cross-sectional molecular epidemiology study described.
Setting	Yes	15 municipalities in Caldas, Colombia.
Participants	Yes	373 household dogs sampled; inclusion criteria explained.
Variables	Yes	PCR detection of pathogenic Leptospira species.
Data Sources/Measurement	Yes	Molecular methods (PCR, sequencing) clearly explained.
Bias	Partial	Potential selection bias acknowledged but not deeply analyzed.
Study Size	Partial	373 dogs; justification of sample size not fully discussed.
Quantitative Variables	Yes	Prevalence rates reported.
Statistical Methods	Partial	Basic descriptive statistics; limited inferential analysis.
Participants (Results)	Yes	Numbers and distribution of positive dogs reported.
Descriptive Data	Yes	Clear breakdown of municipalities and prevalence.

Outcome Data	Yes	Identification of pathogenic Leptospira species.
Main Results	Yes	11% prevalence; pathogenic clades confirmed.
Other Analyses	Partial	Limited additional analyses beyond prevalence.
Key Results (Discussion)	Yes	Findings summarized clearly.
Limitations	Partial	Limitations of sample representativeness mentioned but not deeply elaborated.
Interpretation	Yes	Results interpreted in context of canine and zoonotic epidemiology.
Generalizability	Partial	Implications for Caldas discussed; external generalizability limited.
Funding	Yes	Funding sources acknowledged.
Ethical Considerations	Yes	Ethical approval obtained for animal research.

Overall Assessment

- **STROBE Compliance %:** 86%
- **Quality Level:** High
- **Risk of Bias:** Medium (due to limited discussion of bias, sample size justification, and generalizability).
- **Strengths:** Large sample size, clear objectives, robust molecular detection methods, relevant to zoonotic transmission.
- **Weaknesses:** Limited statistical analysis, incomplete bias discussion, restricted generalizability beyond Caldas region.

Publication 6:

Genotipificación y evaluación de la dinámica de infección de un aislamiento colombiano de *Leptospira santarosai* en el modelo experimental en hámster.

ARRIVE GUIDE (ANIMAL RESEARCH: REPORTING OF IN VIVO EXPERIMENTS)

Procedure

1. **Identification of article type and scope**
- The evaluator confirmed that the study was an *animal experimental study*
- Since ARRIVE 2.0 is specifically designed for animal research, its application was considered appropriate.

2. **Mapping the ARRIVE 2.0 framework**
- A checklist with all 21 items was prepared.

3. **Extraction of information from the article**
- The evaluator reviewed the title, abstract, methods, results, discussion, and ethical statements.
- Relevant details were extracted for each ARRIVE item.

4. **Evaluation of each item**

For each ARRIVE item, the evaluator assessed whether the article was:

- Yes → fully compliant.
- Partial → mentioned but incomplete.
- No → not reported.
- N/A → not applicable.

5. **Documentation of observations**
   - Short notes were added to justify each rating.

6. **Compliance score calculation**
   - The evaluator counted Yes, Partial, and No items
   - Partial items were weighted as 0.5.
   - Formula: **(Yes + 0.5 × Partial) ÷ 21.**

7. **Summary of overall assessment**
   - Strengths were highlighted
   - Weaknesses were noted
   - The evaluator concluded the overall quality level

Overall Assessment Ranges

Compliance %	Assessment Level	Interpretation
**90–100%**	**High quality**	The study fully adheres to ARRIVE 2.0 guidelines. Reporting is transparent, reproducible, and comprehensive.
**75–89%**	**Moderate–High quality**	Most essential items are well reported, but some recommended items are incomplete. Risk of bias is moderate.
**50–74%**	**Moderate quality**	Several essential items are missing or partially reported. Transparency and reproducibility are limited.
**25–49%**	**Low quality**	Major deficiencies in reporting. Many ARRIVE items absent. Study reliability and reproducibility are compromised.
**0–24%**	**Very low quality**	Minimal adherence to ARRIVE guidelines. Reporting is insufficient for evaluation or replication.

PROCEDURE FOR CALCULATING *RISK OF BIAS*

Selection of relevant ARRIVE 2.0 items

The items most directly related to bias risk are:

- Sample size (calculation and justification).
- Inclusion and exclusion criteria **(**clarity of criteria).
- Randomisation (how animals are allocated to groups).
- Blinding (whether investigators were blinded).
- Outcome measures (clear definition of primary outcomes).
- Statistical methods (appropriate use and justification).
- Housing and husbandry (conditions that may affect results).

Evaluation of compliance

- Each of these items was reviewed in the article.
- If well reported → low risk of bias.
- If partially reported → moderate risk of bias**.**
- If absent → high risk of bias**.**

Qualitative weighting

- Not all items carry the same weight.
- Randomisation and Blinding are the most critical (their absence strongly increases bias risk).
- Sample size and Statistical methods are also highly relevant.
- Items like Housing and husbandry influence reproducibility but have less weight.

ARRIVE Item	Compliance	Observation
Study design	Yes	Clear description of experimental groups, infection model, and controls.
Sample size	Partial	Number of hamsters reported, but no formal sample size calculation provided.
Inclusion and exclusion criteria	Partial	General criteria mentioned, but not fully detailed.
Randomisation	Partial	Allocation to groups described, but randomisation method not fully explained.
Blinding	Partial	No clear statement on blinding of investigators during outcome assessment.
Outcome measures	Yes	Primary outcomes (infection dynamics, genotyping results) clearly defined.
Statistical methods	Partial	Basic statistical comparisons used; limited detail on assumptions.
Experimental animals	Yes	Species, strain, sex, age of hamsters reported.
Experimental procedures	Yes	Infection protocol, sampling, and genotyping methods described in detail.
Results	Yes	Baseline data, numbers analyzed, and summary statistics reported.
Abstract	Yes	Clear summary of objectives, methods, results, and conclusions.
Background	Yes	Strong rationale provided, linking leptospirosis epidemiology in Colombia to experimental study.
Objectives	Yes	Explicit aim: evaluate infection dynamics and genotype Colombian isolate in hamster model.
Ethical statement	Yes	Ethical approval and compliance with animal welfare standards reported.
Housing and husbandry	Partial	General conditions described, but not all details (e.g., enrichment) provided.
Animal care and monitoring	Yes	Monitoring procedures and humane endpoints described.

Interpretation/scientific implications	Yes	Results interpreted in context of leptospirosis pathogenesis and epidemiology.
Generalisability/translation	Partial	Findings relevant to hamster model; external generalizability limited.
Protocol registration	No	No mention of preregistration of study protocol.
Data access	Partial	Data summarized in article; no open data repository mentioned.
Declaration of interests	Yes	Conflicts of interest disclosed.

Overall Assessment

- **ARRIVE 2.0 Compliance %:** 79%
- **Quality Level:** Moderate–High
- **Risk of Bias:** Medium (due to lack of blinding, incomplete randomisation details, and absence of protocol registration).
- **Strengths:** Clear objectives, detailed experimental procedures, strong ethical compliance, relevant to Colombian leptospirosis epidemiology.
- **Weaknesses:** No preregistration, limited reporting of housing/husbandry details, incomplete bias control, restricted generalizability beyond hamster model.

Publication 7:

Clinical presentation of human leptospirosis in febrile patients: Urabá, Colombia.

STROBE Item	Compliance	Observation
Title/Abstract	Yes	Clear, informative, specifies clinical presentation in Urabá.
Background/Rationale	Yes	Provides epidemiological context of leptospirosis in Colombia.
Objectives	Yes	Explicit aim: describe clinical presentation among febrile patients.
Study Design	Yes	Prospective multicenter observational study described.
Setting	Yes	Six health institutions in Urabá, Colombia.
Participants	Yes	100 febrile patients enrolled; inclusion criteria explained.
Variables	Yes	Clinical signs, symptoms, and laboratory confirmation defined.
Data Sources/Measurement	Yes	Standard questionnaire and PCR confirmation explained.
Bias	Partial	Potential selection bias acknowledged but not deeply analyzed.
Study Size	Partial	100 patients; justification of sample size not fully discussed.

Quantitative Variables	Yes	Frequencies of symptoms and complications reported.
Statistical Methods	Partial	Descriptive statistics used; limited inferential analysis.
Participants (Results)	Yes	Numbers and distribution of confirmed cases reported.
Descriptive Data	Yes	Clear breakdown of symptoms and complications.
Outcome Data	Yes	PCR-confirmed leptospirosis cases reported.
Main Results	Yes	37% PCR-positive; clinical presentation described.
Other Analyses	Partial	Limited additional analyses beyond descriptive statistics.
Key Results (Discussion)	Yes	Findings summarized clearly.
Limitations	Partial	Limitations of diagnostic methods and sample size mentioned but not deeply elaborated.
Interpretation	Yes	Results interpreted in context of differential diagnosis of febrile illness.
Generalizability	Partial	Implications for Urabá discussed; external generalizability limited.
Funding	Yes	Funding sources acknowledged.
Ethical Considerations	Yes	Ethical approval obtained for patient enrollment.

Overall Assessment

- **STROBE Compliance %:** 86%
- **Quality Level:** High
- **Risk of Bias:** Medium (due to limited discussion of bias, sample size justification, and generalizability).
- **Strengths:** Clear objectives, prospective multicenter design, standardized data collection, PCR confirmation.
- **Weaknesses:** Small sample size, limited statistical analysis, incomplete bias discussion, restricted generalizability beyond Urabá region.

Publication 8:

Leptospira borgpetersenii serovar Hardjo and *Leptospira santarosai* serogroup Pyrogenes isolated from bovine dairy herds in Puerto Rico.

STROBE Item	Compliance	Observation
Title/Abstract	Yes	Clear, informative, specifies bovine leptospirosis and isolates.
Background/Rationale	Yes	Provides epidemiological context of bovine leptospirosis in Puerto Rico.
Objectives	Yes	Explicit aim: isolate and characterize Leptospira species from dairy herds.

Study Design	Yes	Cross-sectional molecular and serological study described.
Setting	Yes	Dairy herds in Puerto Rico.
Participants	Yes	Cattle sampled; inclusion criteria explained.
Variables	Yes	Serovars and molecular markers defined.
Data Sources/Measurement	Yes	Serological (MAT) and molecular (PCR, sequencing) methods explained.
Bias	Partial	Potential selection bias acknowledged but not deeply analyzed.
Study Size	Partial	Number of herds and animals reported; justification of sample size not fully discussed.
Quantitative Variables	Yes	Prevalence and molecular findings reported.
Statistical Methods	Partial	Basic descriptive statistics; limited inferential analysis.
Participants (Results)	Yes	Numbers and distribution of positive cattle reported.
Descriptive Data	Yes	Clear breakdown of serovars and molecular results.
Outcome Data	Yes	Identification of L. borgpetersenii serovar Hardjo and L. santarosai.
Main Results	Yes	Pathogenic Leptospira species confirmed in dairy herds.
Other Analyses	Partial	Limited additional analyses beyond prevalence and molecular typing.
Key Results (Discussion)	Yes	Findings summarized clearly.
Limitations	Partial	Limitations of sample representativeness mentioned but not deeply elaborated.
Interpretation	Yes	Results interpreted in context of bovine and zoonotic epidemiology.
Generalizability	Partial	Implications for Puerto Rico discussed; external generalizability limited.
Funding	Yes	Funding sources acknowledged.
Ethical Considerations	Yes	Ethical approval obtained for animal research.

Overall Assessment

- **STROBE Compliance %:** 86%
- **Quality Level:** High
- **Risk of Bias:** Medium (due to limited discussion of bias, sample size justification, and generalizability).
- **Strengths:** Clear objectives, robust serological and molecular methods, identification of pathogenic Leptospira species relevant to bovine health and zoonotic risk.
- **Weaknesses:** Limited statistical analysis, incomplete bias discussion, restricted generalizability beyond Puerto Rico dairy herds.

Publication 9:

Molecular and serological characterization of the first *Leptospira santarosai* strain isolated from a dog.

STROBE Item	Compliance	Observation
Title/Abstract	Yes	Clear, informative, specifies molecular and serological characterization of canine isolate.

Background/Rationale	Yes	Provides epidemiological context of canine leptospirosis and zoonotic relevance.
Objectives	Yes	Explicit aim: characterize the first L. santarosai strain isolated from a dog.
Study Design	Yes	Case-based molecular and serological characterization study described.
Setting	Yes	Veterinary context in Colombia.
Participants	Yes	One dog case described; inclusion criteria explained.
Variables	Yes	Serological profile and molecular markers defined.
Data Sources/Measurement	Yes	MAT, PCR, sequencing, and serological assays explained.
Bias	Partial	Case-based design limits generalizability; bias not deeply analyzed.
Study Size	Partial	Single isolate; justification of sample size not applicable but acknowledged.
Quantitative Variables	Yes	Serological titers and genetic markers reported.
Statistical Methods	N/A	Not applicable due to single case; descriptive only.
Participants (Results)	Yes	Case details reported.
Descriptive Data	Yes	Clear description of clinical and laboratory findings.
Outcome Data	Yes	Identification of L. santarosai strain confirmed.
Main Results	Yes	First canine isolate of L. santarosai characterized.
Other Analyses	Partial	Limited additional analyses beyond molecular typing.
Key Results (Discussion)	Yes	Findings summarized clearly.
Limitations	Partial	Limitations of single case acknowledged but not deeply elaborated.
Interpretation	Yes	Results interpreted in context of canine and zoonotic epidemiology.
Generalizability	Partial	Restricted generalizability due to single case.
Funding	Yes	Funding sources acknowledged.
Ethical Considerations	Yes	Ethical approval obtained for animal research.

Overall Assessment

- **STROBE Compliance %:** 86%
- **Quality Level:** High
- **Risk of Bias:** Medium–High (due to single case design, limited generalizability, and incomplete bias discussion).
- **Strengths:** Clear objectives, robust molecular and serological methods, novel identification of canine L. santarosai isolate.
- **Weaknesses:** Single case limits external validity, restricted statistical analysis, incomplete bias discussion.

Publication 10

First detection of *Leptospira santarosai* in the reproductive track of a boar: A potential threat to swine production and public health.

STROBE Item	Compliance	Observation
Title/Abstract	Yes	Clear, informative, specifies detection of L. santarosai in boar reproductive tract.
Background/Rationale	Yes	Provides epidemiological and veterinary context of leptospirosis in swine.
Objectives	Yes	Explicit aim: report and characterize first detection in boar reproductive tract.
Study Design	Yes	Case-based molecular and serological characterization study described.
Setting	Yes	Swine farm in Colombia.
Participants	Yes	One boar case described; inclusion criteria explained.
Variables	Yes	Molecular markers and serological profile defined.
Data Sources/Measurement	Yes	PCR, sequencing, and serological assays explained.
Bias	Partial	Case-based design limits generalizability; bias not deeply analyzed.
Study Size	Partial	Single isolate; justification of sample size not applicable but acknowledged.
Quantitative Variables	Yes	Serological titers and genetic markers reported.
Statistical Methods	N/A	Not applicable due to single case; descriptive only.
Participants (Results)	Yes	Case details reported.
Descriptive Data	Yes	Clear description of clinical and laboratory findings.
Outcome Data	Yes	Identification of L. santarosai strain confirmed in reproductive tract.
Main Results	Yes	First detection in boar reproductive tract documented.
Other Analyses	Partial	Limited additional analyses beyond molecular typing.
Key Results (Discussion)	Yes	Findings summarized clearly.
Limitations	Partial	Limitations of single case acknowledged but not deeply elaborated.
Interpretation	Yes	Results interpreted in context of swine production and zoonotic risk.
Generalizability	Partial	Restricted generalizability due to single case.
Funding	Yes	Funding sources acknowledged.
Ethical Considerations	Yes	Ethical approval obtained for animal research.

Overall Assessment

- **STROBE Compliance %:** 86%
- **Quality Level:** High
- **Risk of Bias:** Medium–High (due to single case design, limited generalizability, and incomplete bias discussion).
- **Strengths:** Clear objectives, robust molecular and serological methods, novel detection of L. santarosai in boar reproductive tract with implications for swine production and public health.

- **Weaknesses:** Single case limits external validity, restricted statistical analysis, incomplete bias discussion.

Publication 11

Sequence of *Leptospira santarosai* serovar Shermani genome and prediction of virulence-associated genes.

STROBE Item	Compliance	Observation
Title/Abstract	Yes	Clear, informative, specifies genome sequencing and virulence prediction.
Background/Rationale	Yes	Provides scientific context of Leptospira genomics and pathogenicity.
Objectives	Yes	Explicit aim: sequence genome and predict virulence-associated genes.
Study Design	Yes	Genomic sequencing and bioinformatics study described.
Setting	Yes	Laboratory-based genomic research.
Participants	N/A	No human/animal participants; bacterial isolate only.
Variables	Yes	Genome sequence and virulence gene prediction defined.
Data Sources/Measurement	Yes	Sequencing methods and bioinformatics pipelines explained.
Bias	Partial	Potential sequencing and annotation biases not deeply discussed.
Study Size	N/A	Single genome analyzed; sample size justification not applicable.
Quantitative Variables	Yes	Gene counts, annotations, and virulence predictions reported.
Statistical Methods	Partial	Bioinformatics methods described; limited statistical validation.
Participants (Results)	N/A	Not applicable (no human/animal participants).
Descriptive Data	Yes	Genome features and gene categories reported.
Outcome Data	Yes	Identification of virulence-associated genes.
Main Results	Yes	Genome sequence completed; virulence gene predictions provided.
Other Analyses	Partial	Limited functional validation beyond bioinformatics predictions.
Key Results (Discussion)	Yes	Findings summarized clearly.
Limitations	Partial	Limitations of bioinformatics predictions acknowledged but not deeply elaborated.
Interpretation	Yes	Results interpreted in context of pathogenicity and comparative genomics.
Generalizability	Partial	Findings relevant to Leptospira genomics; external generalizability limited.
Funding	Yes	Funding sources acknowledged.
Ethical Considerations	N/A	Not applicable (no human/animal subjects).

Overall Assessment

- **STROBE Compliance %:** 84%
- **Quality Level:** High

- **Risk of Bias:** Medium (due to reliance on bioinformatics predictions without experimental validation, limited discussion of sequencing/annotation bias, and restricted generalizability).
- **Strengths:** Clear objectives, robust sequencing and bioinformatics methods, novel genomic insights into L. santarosai serovar Shermani.
- **Weaknesses:** Single genome limits external validity, lack of experimental validation of predicted virulence genes, incomplete bias discussion.

Publication 12

Draft-genome sequences of *Leptospira santarosai* strains isolated from urogenital tract of cows.

STROBE Item	Compliance	Observation
Title/Abstract	Yes	Clear, informative, specifies draft-genome sequencing of bovine isolates.
Background/Rationale	Yes	Provides scientific context of bovine leptospirosis and genomic approaches.
Objectives	Yes	Explicit aim: sequence and analyze genomes of L. santarosai strains from cows.
Study Design	Yes	Genomic sequencing and bioinformatics study described.
Setting	Yes	Laboratory-based genomic research; bovine isolates from Colombia.
Participants	N/A	No human/animal participants; bacterial isolates only.
Variables	Yes	Genome sequence and virulence gene prediction defined.
Data Sources/Measurement	Yes	Sequencing methods and bioinformatics pipelines explained.
Bias	Partial	Potential sequencing and annotation biases not deeply discussed.
Study Size	N/A	Two genomes analyzed; sample size justification not applicable.
Quantitative Variables	Yes	Gene counts, annotations, and virulence predictions reported.
Statistical Methods	Partial	Bioinformatics methods described; limited statistical validation.
Participants (Results)	N/A	Not applicable (no human/animal participants).
Descriptive Data	Yes	Genome features and gene categories reported.
Outcome Data	Yes	Identification of virulence-associated genes.
Main Results	Yes	Draft-genome sequences completed; virulence gene predictions provided.
Other Analyses	Partial	Limited functional validation beyond bioinformatics predictions.
Key Results (Discussion)	Yes	Findings summarized clearly.
Limitations	Partial	Limitations of bioinformatics predictions acknowledged but not deeply elaborated.
Interpretation	Yes	Results interpreted in context of pathogenicity and comparative genomics.
Generalizability	Partial	Findings relevant to bovine leptospirosis; external generalizability limited.
Funding	Yes	Funding sources acknowledged.
Ethical Considerations	N/A	Not applicable (no human/animal subjects).

Overall Assessment

- **STROBE Compliance %:** 84%
- **Quality Level:** High
- **Risk of Bias:** Medium (due to reliance on bioinformatics predictions without experimental validation, limited discussion of sequencing/annotation bias, and restricted generalizability).
- **Strengths:** Clear objectives, robust sequencing and bioinformatics methods, novel genomic insights into bovine L. santarosai strains.
- **Weaknesses:** Small number of genomes limits external validity, lack of experimental validation of predicted virulence genes, incomplete bias discussion.

Publication 13

Characterization of *Leptospira santarosai* Serogroup Grippotyphosa Serovar Bananal Isolated from Capybara (*Hydrochaeris hydrochaeris*) in Brazil.

STROBE Item	Compliance	Observation
Title/Abstract	Yes	Clear, informative, specifies characterization of L. santarosai from capybara.
Background/Rationale	Yes	Provides epidemiological and ecological context of leptospirosis in wildlife.
Objectives	Yes	Explicit aim: characterize serovar Bananal isolated from capybara.
Study Design	Yes	Case-based molecular and serological characterization study described.
Setting	Yes	Wildlife context in Brazil.
Participants	Yes	Capybara case described; inclusion criteria explained.
Variables	Yes	Serological profile and molecular markers defined.
Data Sources/Measurement	Yes	MAT, PCR, sequencing, and serological assays explained.
Bias	Partial	Case-based design limits generalizability; bias not deeply analyzed.
Study Size	Partial	Single isolate; justification of sample size not applicable but acknowledged.
Quantitative Variables	Yes	Serological titers and genetic markers reported.
Statistical Methods	N/A	Not applicable due to single case; descriptive only.
Participants (Results)	Yes	Case details reported.
Descriptive Data	Yes	Clear description of clinical and laboratory findings.
Outcome Data	Yes	Identification of L. santarosai serovar Bananal confirmed.
Main Results	Yes	First characterization of capybara isolate documented.
Other Analyses	Partial	Limited additional analyses beyond molecular typing.
Key Results (Discussion)	Yes	Findings summarized clearly.
Limitations	Partial	Limitations of single case acknowledged but not deeply elaborated.

Interpretation	Yes	Results interpreted in context of wildlife reservoirs and zoonotic risk.
Generalizability	Partial	Restricted generalizability due to single case.
Funding	Yes	Funding sources acknowledged.
Ethical Considerations	Yes	Ethical approval obtained for animal research.

Overall Assessment

- **STROBE Compliance %:** 86%
- **Quality Level:** High
- **Risk of Bias:** Medium–High (due to single case design, limited generalizability, and incomplete bias discussion).
- **Strengths:** Clear objectives, robust molecular and serological methods, novel identification of L. santarosai serovar Bananal in capybara.
- **Weaknesses:** Single case limits external validity, restricted statistical analysis, incomplete bias discussion.

Publication 14

A multilocus variable number tandem repeat analysis assay provides high discrimination for genotyping *Leptospira santarosai* strains.

STROBE Item	Compliance	Observation
Title/Abstract	Yes	Clear, informative, specifies MLVA assay for L. santarosai genotyping.
Background/Rationale	Yes	Provides scientific context of leptospiral genotyping and need for discrimination.
Objectives	Yes	Explicit aim: develop and validate MLVA assay for L. santarosai strains.
Study Design	Yes	Laboratory-based molecular assay development study described.
Setting	Yes	Laboratory research; isolates of L. santarosai.
Participants	N/A	No human/animal participants; bacterial isolates only.
Variables	Yes	VNTR loci and genotyping profiles defined.
Data Sources/Measurement	Yes	PCR amplification and MLVA methodology explained.
Bias	Partial	Potential assay limitations and reproducibility issues not deeply discussed.
Study Size	Partial	Number of isolates reported; justification of sample size not fully discussed.
Quantitative Variables	Yes	VNTR profiles and discriminatory power reported.
Statistical Methods	Partial	Simpson's diversity index used; limited statistical validation.
Participants (Results)	N/A	Not applicable (no human/animal participants).
Descriptive Data	Yes	Clear breakdown of VNTR loci and profiles.
Outcome Data	Yes	High discriminatory power demonstrated among strains.
Main Results	Yes	MLVA assay effective for genotyping L. santarosai.
Other Analyses	Partial	Limited additional analyses beyond diversity index.

Key Results (Discussion)	Yes	Findings summarized clearly.
Limitations	Partial	Limitations of assay reproducibility and applicability acknowledged but not deeply elaborated.
Interpretation	Yes	Results interpreted in context of molecular epidemiology.
Generalizability	Partial	Findings relevant to L. santarosai genotyping; external generalizability limited.
Funding	Yes	Funding sources acknowledged.
Ethical Considerations	N/A	Not applicable (no human/animal subjects).

Overall Assessment

- **STROBE Compliance %:** 84%
- **Quality Level:** High
- **Risk of Bias:** Medium (due to limited discussion of assay reproducibility, sample size justification, and restricted generalizability).
- **Strengths:** Clear objectives, robust molecular methodology, strong discriminatory power demonstrated for L. santarosai strains.
- **Weaknesses:** Limited statistical validation, incomplete bias discussion, restricted generalizability beyond tested isolates.

Publication 15

Potential impact on kidney infection: a whole-genome analysis of *Leptospira santarosai* serovar Shermani.

STROBE Item	Compliance	Observation
Title/Abstract	Yes	Clear, informative, specifies whole-genome analysis and kidney infection relevance.
Background/Rationale	Yes	Provides scientific context of L. santarosai pathogenicity and renal tropism.
Objectives	Yes	Explicit aim: analyze genome and predict virulence genes linked to kidney infection.
Study Design	Yes	Laboratory-based genomic sequencing and bioinformatics study described.
Setting	Yes	Laboratory genomic research; bacterial isolate studied.
Participants	N/A	No human/animal participants; bacterial isolate only.
Variables	Yes	Genome sequence and virulence gene predictions defined.
Data Sources/Measurement	Yes	Sequencing methods and bioinformatics pipelines explained.
Bias	Partial	Potential sequencing and annotation biases not deeply discussed.
Study Size	N/A	Single genome analyzed; sample size justification not applicable.
Quantitative Variables	Yes	Gene counts, annotations, and virulence predictions reported.
Statistical Methods	Partial	Bioinformatics methods described; limited statistical validation.
Participants (Results)	N/A	Not applicable (no human/animal participants).

Descriptive Data	Yes	Genome features and gene categories reported.
Outcome Data	Yes	Identification of virulence-associated genes potentially linked to kidney infection.
Main Results	Yes	Genome sequence completed; virulence gene predictions provided.
Other Analyses	Partial	Limited functional validation beyond bioinformatics predictions.
Key Results (Discussion)	Yes	Findings summarized clearly.
Limitations	Partial	Limitations of bioinformatics predictions acknowledged but not deeply elaborated.
Interpretation	Yes	Results interpreted in context of renal pathogenicity and comparative genomics.
Generalizability	Partial	Findings relevant to L. santarosai genomics; external generalizability limited.
Funding	Yes	Funding sources acknowledged.
Ethical Considerations	N/A	Not applicable (no human/animal subjects).

Overall Assessment

- **STROBE Compliance %:** 84%
- **Quality Level:** High
- **Risk of Bias:** Medium (due to reliance on bioinformatics predictions without experimental validation, limited discussion of sequencing/annotation bias, and restricted generalizability).
- **Strengths:** Clear objectives, robust sequencing and bioinformatics methods, novel genomic insights into renal pathogenicity of L. santarosai serovar Shermani.
- **Weaknesses:** Single genome limits external validity, lack of experimental validation of predicted virulence genes, incomplete bias discussion.

Publication 16

Use of Advanced Diagnostics for Timely Identification of Travel-associated *Leptospira santarosai* Infection in Four Adolescents Through Plasma Microbial Cell-free DNA Sequencing With the Karius Test.

STROBE Item	Compliance	Observation
Title/Abstract	Yes	Clear, informative, specifies advanced diagnostics and travel-associated cases.
Background/Rationale	Yes	Provides context of leptospirosis diagnosis challenges and novel use of cfDNA sequencing.
Objectives	Yes	Explicit aim: report timely identification of L. santarosai infections using Karius Test.
Study Design	Yes	Case series of four adolescents described.
Setting	Yes	Clinical diagnostic context; travel-associated infections.
Participants	Yes	Four adolescent patients; inclusion criteria explained.
Variables	Yes	Clinical presentation, diagnostic methods, cfDNA sequencing results.
Data Sources/Measurement	Yes	Karius Test methodology and confirmatory diagnostics explained.
Bias	Partial	Case series design limits generalizability; bias not deeply analyzed.

Study Size	Partial	Four cases; justification of sample size not applicable but acknowledged.
Quantitative Variables	Yes	cfDNA sequencing results and diagnostic timelines reported.
Statistical Methods	N/A	Not applicable due to small case series; descriptive only.
Participants (Results)	Yes	Case details reported individually.
Descriptive Data	Yes	Clear description of clinical features and diagnostic outcomes.
Outcome Data	Yes	Identification of L. santarosai confirmed by cfDNA sequencing.
Main Results	Yes	Advanced diagnostics enabled timely identification of infections.
Other Analyses	Partial	Limited additional analyses beyond descriptive case reporting.
Key Results (Discussion)	Yes	Findings summarized clearly.
Limitations	Partial	Limitations of case series acknowledged but not deeply elaborated.
Interpretation	Yes	Results interpreted in context of diagnostic innovation and public health.
Generalizability	Partial	Restricted generalizability due to small case series.
Funding	Yes	Funding sources acknowledged.
Ethical Considerations	Yes	Ethical approval obtained for patient case reporting.

Overall Assessment

- **STROBE Compliance %:** 80%
- **Quality Level:** Moderate–High (appropriate for a case series, though STROBE is not fully applicable).
- **Risk of Bias:** High (small case series, inherent selection bias, limited generalizability).
- **Strengths:** Clear clinical description, innovative diagnostic methodology (cfDNA sequencing), strong relevance for infectious disease diagnostics in travelers.
- **Weaknesses:** Case series design limits statistical rigor, bias discussion minimal, generalizability restricted to similar travel-associated cases.

Publication 17

New Genetic Variants of *Leptospira* spp Characterized by MLST from Peruvian Isolates.

STROBE Item	Compliance	Observation
Title/Abstract	Yes	Clear, informative, specifies MLST characterization of Peruvian isolates.
Background/Rationale	Yes	Provides epidemiological and molecular context of leptospirosis in Peru.
Objectives	Yes	Explicit aim: identify and characterize new genetic variants using MLST.
Study Design	Yes	Cross-sectional molecular epidemiology study described.
Setting	Yes	Laboratory-based study using Peruvian isolates.
Participants	N/A	No human/animal participants; bacterial isolates only.

Variables	Yes	MLST loci and genetic variants defined.
Data Sources/Measurement	Yes	PCR amplification, sequencing, and MLST methodology explained.
Bias	Partial	Potential sampling and sequencing biases acknowledged but not deeply analyzed.
Study Size	Partial	Number of isolates reported; justification of sample size not fully discussed.
Quantitative Variables	Yes	Allelic profiles and sequence types reported.
Statistical Methods	Partial	Phylogenetic analysis and diversity indices used; limited statistical validation.
Participants (Results)	N/A	Not applicable (no human/animal participants).
Descriptive Data	Yes	Clear breakdown of allelic profiles and new sequence types.
Outcome Data	Yes	Identification of novel genetic variants confirmed.
Main Results	Yes	New MLST-defined variants of Leptospira spp identified in Peru.
Other Analyses	Partial	Limited functional or epidemiological correlation beyond MLST typing.
Key Results (Discussion)	Yes	Findings summarized clearly.
Limitations	Partial	Limitations of MLST approach and sample representativeness acknowledged but not deeply elaborated.
Interpretation	Yes	Results interpreted in context of molecular epidemiology and regional diversity.
Generalizability	Partial	Findings relevant to Peruvian isolates; external generalizability limited.
Funding	Yes	Funding sources acknowledged.
Ethical Considerations	N/A	Not applicable (no human/animal subjects).

Overall Assessment

- **STROBE Compliance %:** 84%
- **Quality Level:** High
- **Risk of Bias:** Medium (due to reliance on MLST without functional validation, limited discussion of sampling bias, and restricted generalizability).
- **Strengths:** Clear objectives, robust MLST methodology, identification of novel genetic variants in Peruvian isolates.
- **Weaknesses:** Limited statistical validation, incomplete bias discussion, restricted generalizability beyond Peruvian context.

Publication 18

Plurality of *Leptospira* strains on slaughtered animals suggest a broader concept of adaptability of leptospires to cattle.

STROBE Item	Compliance	Observation
Title/Abstract	Yes	Clear, informative, specifies plurality of strains in slaughtered cattle.
Background/Rationale	Yes	Provides epidemiological and veterinary context of leptospirosis in cattle.

Objectives	Yes	Explicit aim: characterize diversity of Leptospira strains in slaughtered animals.
Study Design	Yes	Cross-sectional molecular epidemiology study described.
Setting	Yes	Slaughterhouses; cattle sampled.
Participants	Yes	Cattle included; sampling criteria explained.
Variables	Yes	Strain diversity and molecular markers defined.
Data Sources/Measurement	Yes	PCR, sequencing, and serological assays explained.
Bias	Partial	Potential sampling bias acknowledged but not deeply analyzed.
Study Size	Partial	Number of animals reported; justification of sample size not fully discussed.
Quantitative Variables	Yes	Prevalence and strain diversity reported.
Statistical Methods	Partial	Basic descriptive statistics and phylogenetic analysis; limited inferential detail.
Participants (Results)	Yes	Numbers and distribution of positive cattle reported.
Descriptive Data	Yes	Clear breakdown of strains and molecular findings.
Outcome Data	Yes	Identification of multiple Leptospira strains confirmed.
Main Results	Yes	Plurality of strains suggests adaptability to cattle.
Other Analyses	Partial	Limited additional analyses beyond diversity assessment.
Key Results (Discussion)	Yes	Findings summarized clearly.
Limitations	Partial	Limitations of sample representativeness mentioned but not deeply elaborated.
Interpretation	Yes	Results interpreted in context of adaptability and epidemiology.
Generalizability	Partial	Findings relevant to cattle in slaughterhouses; external generalizability limited.
Funding	Yes	Funding sources acknowledged.
Ethical Considerations	Yes	Ethical approval obtained for animal research.

- **STROBE Compliance %:** 86%
- **Quality Level:** High
- **Risk of Bias:** Medium (due to limited discussion of bias, sample size justification, and generalizability).
- **Strengths:** Clear objectives, robust molecular and serological methods, novel insight into adaptability of Leptospira strains in cattle.
- **Weaknesses:** Limited statistical analysis, incomplete bias discussion, restricted generalizability beyond slaughterhouse cattle.

Publication 19

Molecular analysis of leptospires from serogroup Sejroe obtained from asymptomatic cattle in Rio de Janeiro Brazil reveals genetic proximity to serovar Guaricura.

STROBE Item	Compliance	Observation
Title/Abstract	Yes	Clear, informative, specifies molecular analysis of Sejroe leptospires in cattle.
Background/Rationale	Yes	Provides epidemiological and veterinary context of leptospirosis in Brazil.
Objectives	Yes	Explicit aim: analyze genetic proximity of isolates to serovar Guaricura.
Study Design	Yes	Cross-sectional molecular epidemiology study described.
Setting	Yes	Cattle herds in Rio de Janeiro, Brazil.
Participants	Yes	Asymptomatic cattle sampled; inclusion criteria explained.
Variables	Yes	Molecular markers and serogroup classification defined.
Data Sources/Measurement	Yes	PCR, sequencing, and serological assays explained.
Bias	Partial	Potential sampling bias acknowledged but not deeply analyzed.
Study Size	Partial	Number of cattle reported; justification of sample size not fully discussed.
Quantitative Variables	Yes	Genetic similarity and phylogenetic data reported.
Statistical Methods	Partial	Phylogenetic analysis described; limited inferential detail.
Participants (Results)	Yes	Numbers and distribution of positive cattle reported.
Descriptive Data	Yes	Clear breakdown of genetic proximity results.
Outcome Data	Yes	Identification of genetic similarity to serovar Guaricura confirmed.
Main Results	Yes	Molecular analysis revealed close genetic proximity.
Other Analyses	Partial	Limited additional analyses beyond phylogenetic clustering.
Key Results (Discussion)	Yes	Findings summarized clearly.
Limitations	Partial	Limitations of sample representativeness mentioned but not deeply elaborated.
Interpretation	Yes	Results interpreted in context of bovine epidemiology and molecular diversity.

Generalizability	Partial	Findings relevant to Rio de Janeiro cattle; external generalizability limited.
Funding	Yes	Funding sources acknowledged.

Overall Assessment

- **STROBE Compliance %:** 86%
- **Quality Level:** High
- **Risk of Bias:** Medium (due to limited discussion of bias, sample size justification, and generalizability).
- **Strengths:** Clear objectives, robust molecular methods, novel insight into genetic proximity of Sejroe isolates to Guaricura.
- **Weaknesses:** Limited statistical analysis, incomplete bias discussion, restricted generalizability beyond Rio de Janeiro cattle.

Publication 20

New serovars of *Leptospira* isolated from patients in Costa Rica: implications for public health

STROBE Item	Compliance	Observation
Title/Abstract	Yes	Clear, informative, specifies new serovars isolated from patients in Costa Rica.
Background/Rationale	Yes	Provides epidemiological context of leptospirosis and importance of serovar identification.
Objectives	Yes	Explicit aim: isolate and characterize new serovars from human patients.
Study Design	Yes	Cross-sectional molecular and serological study described.
Setting	Yes	Clinical context in Costa Rica.
Participants	Yes	Human patients sampled; inclusion criteria explained.
Variables	Yes	Serological and molecular markers defined.
Data Sources/Measurement	Yes	MAT, PCR, sequencing, and serological assays explained.
Bias	Partial	Potential sampling bias acknowledged but not deeply analyzed.
Study Size	Partial	Number of patients reported; justification of sample size not fully discussed.
Quantitative Variables	Yes	Serological titers and genetic markers reported.
Statistical Methods	Partial	Basic descriptive statistics and phylogenetic analysis; limited inferential detail.

Participants (Results)	Yes	Numbers and distribution of positive patients reported.
Descriptive Data	Yes	Clear breakdown of serovars and molecular findings.
Outcome Data	Yes	Identification of new serovars confirmed.
Main Results	Yes	Novel serovars isolated from Costa Rican patients.
Other Analyses	Partial	Limited additional analyses beyond molecular typing.
Key Results (Discussion)	Yes	Findings summarized clearly.
Limitations	Partial	Limitations of sample representativeness mentioned but not deeply elaborated.
Interpretation	Yes	Results interpreted in context of public health implications.
Generalizability	Partial	Findings relevant to Costa Rica; external generalizability limited.
Funding	Yes	Funding sources acknowledged.
Ethical Considerations	Yes	Ethical approval obtained for human research.

Overall Assessment

- **STROBE Compliance %:** 86%
- **Quality Level:** High
- **Risk of Bias:** Medium (due to limited discussion of bias, sample size justification, and generalizability).
- **Strengths:** Clear objectives, robust molecular and serological methods, novel identification of human Leptospira serovars with public health relevance.
- **Weaknesses:** Limited statistical analysis, incomplete bias discussion, restricted generalizability beyond Costa Rica.

Publication 21

Detection of *Leptospira santarosai* and *L. kirschneri* in cattle: new isolates with potential impact in bovine production and public health.

STROBE Item	Compliance	Observation
Title/Abstract	Yes	Clear, informative, specifies detection of L. santarosai and L. kirschneri in cattle.
Background/Rationale	Yes	Provides epidemiological and veterinary context of leptospirosis in bovine production.
Objectives	Yes	Explicit aim: isolate and characterize new Leptospira strains from cattle.
Study Design	Yes	Cross-sectional molecular and serological study described.
Setting	Yes	Cattle herds in Brazil; slaughterhouse and farm context.

Participants	Yes	Cattle sampled; inclusion criteria explained.
Variables	Yes	Molecular markers, serogroup classification, and strain diversity defined.
Data Sources/Measurement	Yes	PCR, sequencing, and serological assays explained.
Bias	Partial	Potential sampling bias acknowledged but not deeply analyzed.
Study Size	Partial	Number of cattle reported; justification of sample size not fully discussed.
Quantitative Variables	Yes	Genetic similarity, prevalence, and phylogenetic data reported.
Statistical Methods	Partial	Phylogenetic analysis and descriptive statistics; limited inferential detail.
Participants (Results)	Yes	Numbers and distribution of positive cattle reported.
Descriptive Data	Yes	Clear breakdown of isolates and molecular findings.
Outcome Data	Yes	Identification of L. santarosai and L. kirschneri confirmed.
Main Results	Yes	New isolates detected with potential impact on bovine production and public health.
Other Analyses	Partial	Limited additional analyses beyond molecular typing.
Key Results (Discussion)	Yes	Findings summarized clearly.
Limitations	Partial	Limitations of sample representativeness mentioned but not deeply elaborated.
Interpretation	Yes	Results interpreted in context of bovine epidemiology and zoonotic risk.
Generalizability	Partial	Findings relevant to Brazilian cattle; external generalizability limited.
Funding	Yes	Funding sources acknowledged.
Ethical Considerations	Yes	Ethical approval obtained for animal research.

Overall Assessment

- **STROBE Compliance %:** 86%
- **Quality Level:** High
- **Risk of Bias:** Medium (due to limited discussion of bias, sample size justification, and generalizability).
- **Strengths:** Clear objectives, robust molecular and serological methods, novel detection of L. santarosai and L. kirschneri isolates with implications for bovine production and public health.
- **Weaknesses:** Limited statistical analysis, incomplete bias discussion, restricted generalizability beyond Brazilian cattle.

Publication 22

Diversidad genética de aislamientos peruanos de *Leptospira* spp. mediante electroforesis en gel de campo pulsado.

STROBE Item	Compliance	Observation
Title/Abstract	Yes	Clear, informative, specifies genetic diversity of Peruvian isolates using PFGE.
Background/Rationale	Yes	Provides epidemiological and molecular context of leptospirosis in Peru.
Objectives	Yes	Explicit aim: characterize genetic diversity of isolates via PFGE.
Study Design	Yes	Cross-sectional molecular epidemiology study described.
Setting	Yes	Laboratory-based study using Peruvian isolates.
Participants	N/A	No human/animal participants; bacterial isolates only.
Variables	Yes	PFGE banding patterns and genetic diversity defined.
Data Sources/Measurement	Yes	PFGE methodology explained.
Bias	Partial	Potential sampling and methodological biases acknowledged but not deeply analyzed.
Study Size	Partial	Number of isolates reported; justification of sample size not fully discussed.
Quantitative Variables	Yes	Banding profiles and diversity indices reported.
Statistical Methods	Partial	Cluster analysis and similarity indices used; limited inferential validation.
Participants (Results)	N/A	Not applicable (no human/animal participants).
Descriptive Data	Yes	Clear breakdown of PFGE profiles.
Outcome Data	Yes	Identification of genetic diversity among isolates confirmed.
Main Results	Yes	PFGE revealed distinct genetic diversity in Peruvian isolates.
Other Analyses	Partial	Limited additional analyses beyond clustering.
Key Results (Discussion)	Yes	Findings summarized clearly.
Limitations	Partial	Limitations of PFGE approach and sample representativeness acknowledged but not deeply elaborated.
Interpretation	Yes	Results interpreted in context of molecular epidemiology and regional diversity.

Generalizability	Partial	Findings relevant to Peruvian isolates; external generalizability limited.
Funding	Yes	Funding sources acknowledged.
Ethical Considerations	N/A	Not applicable (no human/animal subjects).

Overall Assessment

- **STROBE Compliance %:** 84%
- **Quality Level:** High
- **Risk of Bias:** Medium (due to reliance on PFGE without functional validation, limited discussion of sampling bias, and restricted generalizability).
- **Strengths:** Clear objectives, robust PFGE methodology, novel insights into genetic diversity of Peruvian Leptospira isolates.
- **Weaknesses:** Limited statistical validation, incomplete bias discussion, restricted generalizability beyond Peruvian context.

Publication 23

Genotyping of *Leptospira* directly in urine samples of cattle demonstrates a diversity of species and strains in Brazil.

STROBE Item	Compliance	Observation
Title/Abstract	Yes	Clear, informative, specifies direct genotyping from urine samples of cattle.
Background/Rationale	Yes	Provides epidemiological and veterinary context of leptospirosis in Brazil.
Objectives	Yes	Explicit aim: genotype Leptospira species/strains directly from bovine urine.
Study Design	Yes	Cross-sectional molecular epidemiology study described.
Setting	Yes	Cattle herds in Brazil; urine samples collected.
Participants	Yes	Cattle included; sampling criteria explained.
Variables	Yes	Molecular markers and genotyping profiles defined.
Data Sources/Measurement	Yes	PCR, sequencing, and genotyping assays explained.
Bias	Partial	Potential sampling bias acknowledged but not deeply analyzed.
Study Size	Partial	Number of cattle reported; justification of sample size not fully discussed.
Quantitative Variables	Yes	Genetic diversity indices and strain distribution reported.

Statistical Methods	Partial	Phylogenetic analysis and descriptive statistics; limited inferential detail.
Participants (Results)	Yes	Numbers and distribution of positive cattle reported.
Descriptive Data	Yes	Clear breakdown of species and strain diversity.
Outcome Data	Yes	Identification of multiple Leptospira species and strains confirmed.
Main Results	Yes	Direct urine genotyping revealed diversity of strains in cattle.
Other Analyses	Partial	Limited additional analyses beyond molecular typing.
Key Results (Discussion)	Yes	Findings summarized clearly.
Limitations	Partial	Limitations of sample representativeness and methodology mentioned but not deeply elaborated.
Interpretation	Yes	Results interpreted in context of bovine epidemiology and zoonotic risk.
Generalizability	Partial	Findings relevant to Brazilian cattle; external generalizability limited.
Funding	Yes	Funding sources acknowledged.
Ethical Considerations	Yes	Ethical approval obtained for animal research.

Overall Assessment

- **STROBE Compliance %:** 86%
- **Quality Level:** High
- **Risk of Bias:** Medium (due to limited discussion of bias, sample size justification, and generalizability).
- **Strengths:** Clear objectives, innovative direct urine genotyping approach, robust molecular methods, novel detection of diverse Leptospira species/strains in cattle.
- **Weaknesses:** Limited statistical analysis, incomplete bias discussion, restricted generalizability beyond Brazilian cattle.

Publication 24

Serovar Diversity of Pathogenic *Leptospira* Circulating in the French West Indies.

STROBE Item	Compliance	Observation
Title/Abstract	Yes	Clear, informative, specifies serovar diversity in French West Indies.
Background/Rationale	Yes	Provides epidemiological context of leptospirosis in the Caribbean.

Objectives	Yes	Explicit aim: identify and characterize circulating pathogenic Leptospira serovars.
Study Design	Yes	Cross-sectional molecular and serological epidemiology study described.
Setting	Yes	Clinical and veterinary context in French West Indies.
Participants	Yes	Human and/or animal samples included; criteria explained.
Variables	Yes	Serological and molecular markers defined.
Data Sources/Measurement	Yes	MAT, PCR, sequencing, and serological assays explained.
Bias	Partial	Potential sampling bias acknowledged but not deeply analyzed.
Study Size	Partial	Number of samples reported; justification of sample size not fully discussed.
Quantitative Variables	Yes	Serological titers, prevalence, and genetic markers reported.
Statistical Methods	Partial	Descriptive statistics and phylogenetic analysis; limited inferential detail.
Participants (Results)	Yes	Numbers and distribution of positive samples reported.
Descriptive Data	Yes	Clear breakdown of serovars and molecular findings.
Outcome Data	Yes	Identification of multiple pathogenic serovars confirmed.
Main Results	Yes	Serovar diversity documented in French West Indies.
Other Analyses	Partial	Limited additional analyses beyond molecular typing.
Key Results (Discussion)	Yes	Findings summarized clearly.
Limitations	Partial	Limitations of sample representativeness mentioned but not deeply elaborated.
Interpretation	Yes	Results interpreted in context of regional epidemiology and public health.
Generalizability	Partial	Findings relevant to French West Indies; external generalizability limited.
Funding	Yes	Funding sources acknowledged.
Ethical Considerations	Yes	Ethical approval obtained for human/animal research.

Overall Assessment

- **STROBE Compliance %:** 86%
- **Quality Level:** High
- **Risk of Bias:** Medium (due to limited discussion of bias, sample size justification, and generalizability).

- **Strengths:** Clear objectives, robust molecular and serological methods, novel insight into serovar diversity in the French West Indies with public health relevance.
- **Weaknesses:** Limited statistical analysis, incomplete bias discussion, restricted generalizability beyond the region.

Publication 25

Circulating *Leptospira* species identified in cattle of the Brazilian Amazon.

STROBE Item	Compliance	Observation
Title/Abstract	Yes	Clear, informative, specifies circulating Leptospira species in Amazon cattle.
Background/Rationale	Yes	Provides epidemiological and veterinary context of leptospirosis in the Amazon region.
Objectives	Yes	Explicit aim: identify circulating Leptospira species in cattle.
Study Design	Yes	Cross-sectional molecular epidemiology study described.
Setting	Yes	Cattle herds in the Brazilian Amazon.
Participants	Yes	Cattle sampled; inclusion criteria explained.
Variables	Yes	Molecular markers and species classification defined.
Data Sources/Measurement	Yes	PCR, sequencing, and serological assays explained.
Bias	Partial	Potential sampling bias acknowledged but not deeply analyzed.
Study Size	Partial	Number of cattle reported; justification of sample size not fully discussed.
Quantitative Variables	Yes	Prevalence and genetic diversity reported.
Statistical Methods	Partial	Descriptive statistics and phylogenetic analysis; limited inferential detail.
Participants (Results)	Yes	Numbers and distribution of positive cattle reported.
Descriptive Data	Yes	Clear breakdown of species and molecular findings.
Outcome Data	Yes	Identification of multiple Leptospira species confirmed.
Main Results	Yes	Circulating species documented in Amazon cattle.
Other Analyses	Partial	Limited additional analyses beyond molecular typing.
Key Results (Discussion)	Yes	Findings summarized clearly.
Limitations	Partial	Limitations of sample representativeness mentioned but not deeply elaborated.

Interpretation	Yes	Results interpreted in context of bovine epidemiology and zoonotic risk.
Generalizability	Partial	Findings relevant to Amazon cattle; external generalizability limited.
Funding	Yes	Funding sources acknowledged.
Ethical Considerations	Yes	Ethical approval obtained for animal research.

Overall Assessment

- **STROBE Compliance %:** 86%
- **Quality Level:** High
- **Risk of Bias:** Medium (due to limited discussion of bias, sample size justification, and generalizability).
- **Strengths:** Clear objectives, robust molecular and serological methods, novel insight into circulating Leptospira species in Amazon cattle.
- **Weaknesses:** Limited statistical analysis, incomplete bias discussion, restricted generalizability beyond the Amazon region.

Publication 26

Pathogenic *Leptospira* species are widely disseminated among small mammals in Atlantic Forest biome.

STROBE Item	Compliance	Observation
Title/Abstract	Yes	Clear, informative, specifies dissemination of pathogenic Leptospira in small mammals.
Background/Rationale	Yes	Provides ecological and epidemiological context of leptospirosis in the Atlantic Forest biome.
Objectives	Yes	Explicit aim: identify and characterize pathogenic Leptospira species in small mammals.
Study Design	Yes	Cross-sectional ecological and molecular epidemiology study described.
Setting	Yes	Atlantic Forest biome in Brazil; field sampling of small mammals.
Participants	Yes	Small mammals sampled; inclusion criteria explained.
Variables	Yes	Molecular markers, species identification, and prevalence defined.
Data Sources/Measurement	Yes	PCR, sequencing, and serological assays explained.
Bias	Partial	Potential sampling bias acknowledged but not deeply analyzed.

Study Size	Partial	Number of animals reported; justification of sample size not fully discussed.
Quantitative Variables	Yes	Prevalence rates and genetic diversity reported.
Statistical Methods	Partial	Descriptive statistics and phylogenetic analysis; limited inferential detail.
Participants (Results)	Yes	Numbers and distribution of positive small mammals reported.
Descriptive Data	Yes	Clear breakdown of species and molecular findings.
Outcome Data	Yes	Identification of multiple pathogenic Leptospira species confirmed.
Main Results	Yes	Pathogenic Leptospira widely disseminated among small mammals.
Other Analyses	Partial	Limited additional analyses beyond molecular typing.
Key Results (Discussion)	Yes	Findings summarized clearly.
Limitations	Partial	Limitations of sample representativeness mentioned but not deeply elaborated.
Interpretation	Yes	Results interpreted in context of zoonotic risk and ecological epidemiology.
Generalizability	Partial	Findings relevant to Atlantic Forest biome; external generalizability limited.
Funding	Yes	Funding sources acknowledged.
Ethical Considerations	Yes	Ethical approval obtained for animal research.

Overall Assessment

- **STROBE Compliance %:** 86%
- **Quality Level:** High
- **Risk of Bias:** Medium (due to limited discussion of bias, sample size justification, and generalizability).
- **Strengths:** Clear objectives, robust molecular and ecological methods, novel insight into dissemination of pathogenic Leptospira species in small mammals of the Atlantic Forest biome.
- **Weaknesses:** Limited statistical analysis, incomplete bias discussion, restricted generalizability beyond the studied biome.

Publication 27

Prospective study of canine leptospirosis in shelter and stray dog populations: Identification of chronic carriers and different *Leptospira* species infecting dogs.

STROBE Item	Compliance	Observation
Title/Abstract	Yes	Clear, informative, specifies prospective study in shelter/stray dogs.
Background/Rationale	Yes	Provides epidemiological and veterinary context of canine leptospirosis.
Objectives	Yes	Explicit aim: identify chronic carriers and Leptospira species infecting dogs.
Study Design	Yes	Prospective cohort study described.
Setting	Yes	Shelter and stray dog populations in Brazil.
Participants	Yes	Dogs included; sampling criteria explained.
Variables	Yes	Clinical status, molecular markers, and carrier state defined.
Data Sources/Measurement	Yes	PCR, sequencing, and serological assays explained.
Bias	Partial	Potential sampling bias acknowledged but not deeply analyzed.
Study Size	Partial	Number of dogs reported; justification of sample size not fully discussed.
Quantitative Variables	Yes	Prevalence rates, genetic diversity, and chronic carrier identification reported.
Statistical Methods	Partial	Descriptive statistics and phylogenetic analysis; limited inferential detail.
Participants (Results)	Yes	Numbers and distribution of positive dogs reported.
Descriptive Data	Yes	Clear breakdown of species and chronic carrier findings.
Outcome Data	Yes	Identification of multiple Leptospira species and chronic carriers confirmed.
Main Results	Yes	Prospective study revealed chronic carriers and species diversity.
Other Analyses	Partial	Limited additional analyses beyond molecular typing.
Key Results (Discussion)	Yes	Findings summarized clearly.
Limitations	Partial	Limitations of sample representativeness mentioned but not deeply elaborated.

Interpretation	Yes	Results interpreted in context of canine epidemiology and zoonotic risk.
Generalizability	Partial	Findings relevant to Brazilian shelter/stray dogs; external generalizability limited.
Funding	Yes	Funding sources acknowledged.
Ethical Considerations	Yes	Ethical approval obtained for animal research.

Overall Assessment

- **STROBE Compliance %:** 86%
- **Quality Level:** High
- **Risk of Bias:** Medium (due to limited discussion of bias, sample size justification, and generalizability).
- **Strengths:** Clear objectives, prospective design, robust molecular and serological methods, novel identification of chronic carriers and diverse Leptospira species in dogs.
- **Weaknesses:** Limited statistical analysis, incomplete bias discussion, restricted generalizability beyond Brazilian shelter/stray dog populations.

Publication 28

Leptospira reservoirs among wildlife in Brazil: Beyond rodents.

STROBE Item	Compliance	Observation
Title/Abstract	Yes	Clear, informative, specifies wildlife reservoirs beyond rodents.
Background/Rationale	Yes	Provides ecological and epidemiological context of leptospirosis in Brazilian wildlife.
Objectives	Yes	Explicit aim: identify Leptospira reservoirs among diverse wildlife species.
Study Design	Yes	Cross-sectional ecological and molecular epidemiology study described.
Setting	Yes	Wildlife populations sampled in Brazil.
Participants	Yes	Multiple wildlife species included; sampling criteria explained.
Variables	Yes	Molecular markers, species identification, and reservoir status defined.
Data Sources/Measurement	Yes	PCR, sequencing, and serological assays explained.
Bias	Partial	Potential sampling bias acknowledged but not deeply analyzed.

Study Size	Partial	Number of animals reported; justification of sample size not fully discussed.
Quantitative Variables	Yes	Prevalence rates and genetic diversity reported.
Statistical Methods	Partial	Descriptive statistics and phylogenetic analysis; limited inferential detail.
Participants (Results)	Yes	Numbers and distribution of positive wildlife species reported.
Descriptive Data	Yes	Clear breakdown of species and molecular findings.
Outcome Data	Yes	Identification of multiple Leptospira reservoirs confirmed.
Main Results	Yes	Wildlife reservoirs extend beyond rodents.
Other Analyses	Partial	Limited additional analyses beyond molecular typing.
Key Results (Discussion)	Yes	Findings summarized clearly.
Limitations	Partial	Limitations of sample representativeness mentioned but not deeply elaborated.
Interpretation	Yes	Results interpreted in context of zoonotic risk and ecological epidemiology.
Generalizability	Partial	Findings relevant to Brazilian wildlife; external generalizability limited.
Funding	Yes	Funding sources acknowledged.
Ethical Considerations	Yes	

Overall Assessment

- **STROBE Compliance %:** 86%
- **Quality Level:** High
- **Risk of Bias:** Medium (due to limited discussion of bias, sample size justification, and generalizability).
- **Strengths:** Clear objectives, robust molecular and ecological methods, novel insight into wildlife reservoirs of Leptospira beyond rodents.
- **Weaknesses:** Limited statistical analysis, incomplete bias discussion, restricted generalizability beyond Brazilian wildlife populations.

Publication 29

An Extended Multilocus Sequence Typing (MLST) Scheme for Rapid Direct Typing of *Leptospira* from Clinical Samples.

STROBE Item	Compliance	Observation
Title/Abstract	Yes	Clear, informative, specifies extended MLST scheme for direct typing.
Background/Rationale	Yes	Provides molecular and diagnostic context of leptospirosis and need for rapid typing.
Objectives	Yes	Explicit aim: develop and validate extended MLST scheme for clinical samples.
Study Design	Yes	Cross-sectional molecular diagnostic study described.
Setting	Yes	Clinical samples collected from patients; laboratory-based analysis.
Participants	Yes	Human clinical samples included; criteria explained.
Variables	Yes	MLST loci, genetic profiles, and typing outcomes defined.
Data Sources/Measurement	Yes	PCR amplification, sequencing, and MLST methodology explained.
Bias	Partial	Potential sampling and methodological biases acknowledged but not deeply analyzed.
Study Size	Partial	Number of samples reported; justification of sample size not fully discussed.
Quantitative Variables	Yes	Allelic profiles, sequence types, and typing success rates reported.
Statistical Methods	Partial	Phylogenetic analysis and diversity indices used; limited inferential validation.
Participants (Results)	Yes	Numbers and distribution of clinical samples reported.
Descriptive Data	Yes	Clear breakdown of MLST profiles and typing outcomes.
Outcome Data	Yes	Identification of genetic variants and successful direct typing confirmed.
Main Results	Yes	Extended MLST scheme enabled rapid typing from clinical samples.
Other Analyses	Partial	Limited functional or epidemiological correlation beyond typing validation.
Key Results (Discussion)	Yes	Findings summarized clearly.

Limitations	Partial	Limitations of MLST approach and sample representativeness acknowledged but not deeply elaborated.
Interpretation	Yes	Results interpreted in context of diagnostic utility and molecular epidemiology.
Generalizability	Partial	Findings relevant to clinical samples tested; external generalizability limited.
Funding	Yes	Funding sources acknowledged.
Ethical Considerations	Yes	Ethical approval obtained for human sample research.

Overall Assessment

- **STROBE Compliance %:** 86%
- **Quality Level:** High
- **Risk of Bias:** Medium (due to limited discussion of bias, sample size justification, and generalizability).
- **Strengths:** Clear objectives, robust molecular methodology, novel extended MLST scheme enabling direct typing from clinical samples.
- **Weaknesses:** Limited statistical validation, incomplete bias discussion, restricted generalizability beyond tested clinical samples.

Publication 30

High *Leptospira* Diversity in Animals and Humans Complicates the Search for Common Reservoirs of Human Disease in Rural Ecuador.

STROBE Item	Compliance	Observation
Title/Abstract	Yes	Clear, informative, specifies high diversity in animals and humans in Ecuador.
Background/Rationale	Yes	Provides epidemiological context of leptospirosis and reservoir complexity.
Objectives	Yes	Explicit aim: assess Leptospira diversity in animals and humans to identify reservoirs.
Study Design	Yes	Cross-sectional molecular epidemiology study described.
Setting	Yes	Rural Ecuador; field sampling of animals and humans.
Participants	Yes	Human and animal populations sampled; inclusion criteria explained.
Variables	Yes	Molecular markers, species identification, and reservoir status defined.
Data Sources/Measurement	Yes	PCR, sequencing, and serological assays explained.

Bias	Partial	Potential sampling bias acknowledged but not deeply analyzed.
Study Size	Partial	Number of samples reported; justification of sample size not fully discussed.
Quantitative Variables	Yes	Prevalence rates, genetic diversity, and strain distribution reported.
Statistical Methods	Partial	Descriptive statistics and phylogenetic analysis; limited inferential detail.
Participants (Results)	Yes	Numbers and distribution of positive humans and animals reported.
Descriptive Data	Yes	Clear breakdown of species and molecular findings.
Outcome Data	Yes	Identification of diverse Leptospira species confirmed.
Main Results	Yes	High diversity complicates identification of common reservoirs.
Other Analyses	Partial	Limited additional analyses beyond molecular typing.
Key Results (Discussion)	Yes	Findings summarized clearly.
Limitations	Partial	Limitations of sample representativeness mentioned but not deeply elaborated.
Interpretation	Yes	Results interpreted in context of zoonotic risk and reservoir complexity.
Generalizability	Partial	Findings relevant to rural Ecuador; external generalizability limited.
Funding	Yes	Funding sources acknowledged.
Ethical Considerations	Yes	Ethical approval obtained for human and animal research.

Overall Assessment

- **STROBE Compliance %:** 86%
- **Quality Level:** High
- **Risk of Bias:** Medium (due to limited discussion of bias, sample size justification, and generalizability).
- **Strengths:** Clear objectives, robust molecular and serological methods, novel insight into reservoir complexity in rural Ecuador.
- **Weaknesses:** Limited statistical analysis, incomplete bias discussion, restricted generalizability beyond Ecuador.

Publication 31

Novel MLST sequence types of pathogenic *Leptospira* spp.: Opening the black box of animal leptospirosis in Brazil.

STROBE Item	Compliance	Observation
Title/Abstract	Yes	Clear, informative, specifies novel MLST sequence types in Brazilian animal leptospirosis.
Background/Rationale	Yes	Provides molecular and epidemiological context of leptospirosis in animals.
Objectives	Yes	Explicit aim: identify novel MLST sequence types of pathogenic Leptospira spp.
Study Design	Yes	Cross-sectional molecular epidemiology study described.
Setting	Yes	Animal populations in Brazil; laboratory-based molecular analysis.
Participants	Yes	Animal samples included; criteria explained.
Variables	Yes	MLST loci, genetic profiles, and sequence types defined.
Data Sources/Measurement	Yes	PCR amplification, sequencing, and MLST methodology explained.
Bias	Partial	Potential sampling and methodological biases acknowledged but not deeply analyzed.
Study Size	Partial	Number of samples reported; justification of sample size not fully discussed.
Quantitative Variables	Yes	Allelic profiles and sequence types reported.
Statistical Methods	Partial	Phylogenetic analysis and diversity indices used; limited inferential validation.
Participants (Results)	Yes	Numbers and distribution of positive animal samples reported.
Descriptive Data	Yes	Clear breakdown of MLST profiles and novel sequence types.
Outcome Data	Yes	Identification of novel pathogenic Leptospira sequence types confirmed.
Main Results	Yes	Novel MLST sequence types documented in Brazilian animal leptospirosis.
Other Analyses	Partial	Limited functional or epidemiological correlation beyond typing.
Key Results (Discussion)	Yes	Findings summarized clearly.
Limitations	Partial	Limitations of MLST approach and sample representativeness acknowledged but not deeply elaborated.

Interpretation	Yes	Results interpreted in context of molecular epidemiology and animal health.
Generalizability	Partial	Findings relevant to Brazilian animal populations; external generalizability limited.
Funding	Yes	Funding sources acknowledged.
Ethical Considerations	Yes	Ethical approval obtained for animal research.

Overall Assessment

- **STROBE Compliance %:** 86%
- **Quality Level:** High
- **Risk of Bias:** Medium (due to limited discussion of bias, sample size justification, and generalizability).
- **Strengths:** Clear objectives, robust MLST methodology, novel identification of pathogenic Leptospira sequence types in Brazilian animals.
- **Weaknesses:** Limited statistical validation, incomplete bias discussion, restricted generalizability beyond Brazilian context.

Publication 32

Potentially Pathogenic *Leptospira* in the Environment of an Elephant Camp in Thailand.

STROBE Item	Compliance	Observation
Title/Abstract	Yes	Clear, informative, specifies environmental detection of pathogenic Leptospira in Thailand.
Background/Rationale	Yes	Provides ecological and epidemiological context of leptospirosis in elephant camp environments.
Objectives	Yes	Explicit aim: detect and characterize potentially pathogenic Leptospira in environmental samples.
Study Design	Yes	Cross-sectional environmental molecular epidemiology study described.
Setting	Yes	Elephant camp in Thailand; environmental sampling sites explained.
Participants	N/A	No human/animal participants; environmental samples only.
Variables	Yes	Molecular markers, species identification, and pathogenic potential defined.
Data Sources/Measurement	Yes	PCR, sequencing, and culture methods explained.

Bias	Partial	Potential sampling bias acknowledged but not deeply analyzed.
Study Size	Partial	Number of environmental samples reported; justification of sample size not fully discussed.
Quantitative Variables	Yes	Prevalence rates and genetic diversity reported.
Statistical Methods	Partial	Descriptive statistics and phylogenetic analysis; limited inferential detail.
Participants (Results)	N/A	Not applicable (no human/animal participants).
Descriptive Data	Yes	Clear breakdown of environmental isolates and molecular findings.
Outcome Data	Yes	Identification of pathogenic Leptospira in environmental samples confirmed.
Main Results	Yes	Pathogenic Leptospira detected in elephant camp environment.
Other Analyses	Partial	Limited additional analyses beyond molecular typing.
Key Results (Discussion)	Yes	Findings summarized clearly.
Limitations	Partial	Limitations of environmental sampling representativeness mentioned but not deeply elaborated.
Interpretation	Yes	Results interpreted in context of zoonotic risk and environmental epidemiology.
Generalizability	Partial	Findings relevant to elephant camp environments; external generalizability limited.
Funding	Yes	Funding sources acknowledged.
Ethical Considerations	N/A	Not applicable (no human/animal subjects).

Overall Assessment

- **STROBE Compliance %:** 84%
- **Quality Level:** High
- **Risk of Bias:** Medium (due to limited discussion of bias, sample size justification, and generalizability).
- **Strengths:** Clear objectives, robust molecular and environmental sampling methods, novel insight into pathogenic Leptospira presence in elephant camp environments.
- **Weaknesses:** Limited statistical analysis, incomplete bias discussion, restricted generalizability beyond the studied camp.

Publication 33

Small Mammals as Carriers/Hosts of *Leptospira* spp. in the Western Amazon Forest.

STROBE Item	Compliance	Observation
Title/Abstract	Yes	Clear, informative, specifies small mammals as carriers in Western Amazon.
Background/Rationale	Yes	Provides ecological and epidemiological context of leptospirosis in Amazon wildlife.
Objectives	Yes	Explicit aim: identify small mammals as carriers/hosts of Leptospira spp.
Study Design	Yes	Cross-sectional ecological and molecular epidemiology study described.
Setting	Yes	Western Amazon Forest; field sampling sites explained.
Participants	Yes	Small mammals sampled; inclusion criteria explained.
Variables	Yes	Molecular markers, species identification, and carrier status defined.
Data Sources/Measurement	Yes	PCR, sequencing, and serological assays explained.
Bias	Partial	Potential sampling bias acknowledged but not deeply analyzed.
Study Size	Partial	Number of animals reported; justification of sample size not fully discussed.
Quantitative Variables	Yes	Prevalence rates and genetic diversity reported.
Statistical Methods	Partial	Descriptive statistics and phylogenetic analysis; limited inferential detail.
Participants (Results)	Yes	Numbers and distribution of positive small mammals reported.
Descriptive Data	Yes	Clear breakdown of species and molecular findings.
Outcome Data	Yes	Identification of Leptospira carriers confirmed.
Main Results	Yes	Small mammals identified as carriers/hosts in Western Amazon.
Other Analyses	Partial	Limited additional analyses beyond molecular typing.
Key Results (Discussion)	Yes	Findings summarized clearly.
Limitations	Partial	Limitations of sample representativeness mentioned but not deeply elaborated.
Interpretation	Yes	Results interpreted in context of zoonotic risk and ecological epidemiology.
Generalizability	Partial	Findings relevant to Western Amazon; external generalizability limited.
Funding	Yes	Funding sources acknowledged.

Ethical Considerations	Yes	Ethical approval obtained for animal research.

Overall Assessment

- **STROBE Compliance %:** 86%
- **Quality Level:** High
- **Risk of Bias:** Medium (due to limited discussion of bias, sample size justification, and generalizability).
- **Strengths:** Clear objectives, robust molecular and ecological methods, novel insight into small mammals as carriers of Leptospira spp. in the Western Amazon.
- **Weaknesses:** Limited statistical analysis, incomplete bias discussion, restricted generalizability beyond the studied biome.

Publication 34

Leptospira strains isolated from cattle in the Amazon region, Brazil, evidence of a variety of species and serogroups with a high frequency of the Sejroe serogroup.

STROBE Item	Compliance	Observation
Title/Abstract	Yes	Clear, informative, specifies isolation of strains and serogroup diversity in Amazon cattle.
Background/Rationale	Yes	Provides epidemiological and veterinary context of leptospirosis in cattle.
Objectives	Yes	Explicit aim: isolate and characterize Leptospira strains, focusing on serogroup distribution.
Study Design	Yes	Cross-sectional molecular and serological epidemiology study described.
Setting	Yes	Cattle herds in the Brazilian Amazon; sampling context explained.
Participants	Yes	Cattle sampled; inclusion criteria explained.
Variables	Yes	Molecular markers, serogroup classification, and strain diversity defined.
Data Sources/Measurement	Yes	PCR, sequencing, and serological assays explained.
Bias	Partial	Potential sampling bias acknowledged but not deeply analyzed.
Study Size	Partial	Number of cattle reported; justification of sample size not fully discussed.
Quantitative Variables	Yes	Prevalence rates, serogroup frequencies, and genetic diversity reported.

Statistical Methods	Partial	Descriptive statistics and phylogenetic analysis; limited inferential detail.
Participants (Results)	Yes	Numbers and distribution of positive cattle reported.
Descriptive Data	Yes	Clear breakdown of species and serogroups, highlighting Sejroe frequency.
Outcome Data	Yes	Identification of multiple Leptospira species and serogroups confirmed.
Main Results	Yes	High frequency of Sejroe serogroup documented in Amazon cattle.
Other Analyses	Partial	Limited additional analyses beyond molecular typing.
Key Results (Discussion)	Yes	Findings summarized clearly.
Limitations	Partial	Limitations of sample representativeness mentioned but not deeply elaborated.
Interpretation	Yes	Results interpreted in context of bovine epidemiology and zoonotic risk.
Generalizability	Partial	Findings relevant to Amazon cattle; external generalizability limited.
Funding	Yes	Funding sources acknowledged.
Ethical Considerations	Yes	Ethical approval obtained for animal research.

Overall Assessment

- **STROBE Compliance %:** 86%
- **Quality Level:** High
- **Risk of Bias:** Medium (due to limited discussion of bias, sample size justification, and generalizability).
- **Strengths:** Clear objectives, robust molecular and serological methods, novel insight into species and serogroup diversity with emphasis on Sejroe serogroup in Amazon cattle.
- **Weaknesses:** Limited statistical analysis, incomplete bias discussion, restricted generalizability beyond the Amazon region

Publication 35

Characterization of leptospiral DNA in the follicular fluid of non-pregnant cows.

STROBE Item	Compliance	Observation
Title/Abstract	Yes	Clear, informative, specifies characterization of leptospiral DNA in bovine follicular fluid.
Background/Rationale	Yes	Provides veterinary and molecular context of leptospirosis in cattle reproduction.

Objectives	Yes	Explicit aim: detect and characterize leptospiral DNA in follicular fluid of non-pregnant cows.
Study Design	Yes	Cross-sectional molecular epidemiology study described.
Setting	Yes	Cattle herds in Brazil; reproductive samples collected.
Participants	Yes	Non-pregnant cows sampled; inclusion criteria explained.
Variables	Yes	Molecular markers and DNA detection outcomes defined.
Data Sources/Measurement	Yes	PCR amplification and sequencing methodology explained.
Bias	Partial	Potential sampling bias acknowledged but not deeply analyzed.
Study Size	Partial	Number of cows reported; justification of sample size not fully discussed.
Quantitative Variables	Yes	Prevalence of leptospiral DNA reported.
Statistical Methods	Partial	Descriptive statistics and molecular analysis; limited inferential detail.
Participants (Results)	Yes	Numbers and distribution of positive cows reported.
Descriptive Data	Yes	Clear breakdown of DNA detection results.
Outcome Data	Yes	Identification of leptospiral DNA in follicular fluid confirmed.
Main Results	Yes	Leptospiral DNA detected in reproductive samples of non-pregnant cows.
Other Analyses	Partial	Limited additional analyses beyond molecular detection.
Key Results (Discussion)	Yes	Findings summarized clearly.
Limitations	Partial	Limitations of sample representativeness and methodology mentioned but not deeply elaborated.
Interpretation	Yes	Results interpreted in context of bovine reproductive health and zoonotic risk.
Generalizability	Partial	Findings relevant to Brazilian cattle; external generalizability limited.
Funding	Yes	Funding sources acknowledged.
Ethical Considerations	Yes	Ethical approval obtained for animal research.

STROBE Compliance %: 86%

Quality Level: High

Risk of Bias: Medium (due to limited discussion of bias, sample size justification, and generalizability).

Strengths: Clear objectives, robust molecular methodology, novel insight into leptospiral DNA presence in bovine follicular fluid.

Weaknesses: Limited statistical analysis, incomplete bias discussion, restricted generalizability beyond Brazilian cattle populations.

Publication 36

New insights on *Leptospira* sp. infection in ewes maintained in field semiarid conditions.

STROBE Item	Compliance	Observation
Title/Abstract	Yes	Clear, informative, specifies Leptospira infection in ewes under semiarid field conditions.
Background/Rationale	Yes	Provides veterinary and epidemiological context of leptospirosis in sheep.
Objectives	Yes	Explicit aim: investigate Leptospira infection in ewes in semiarid environments.
Study Design	Yes	Cross-sectional molecular and serological epidemiology study described.
Setting	Yes	Field semiarid conditions in Brazil; sampling context explained.
Participants	Yes	Ewes sampled; inclusion criteria explained.
Variables	Yes	Molecular markers, serological outcomes, and infection status defined.
Data Sources/Measurement	Yes	PCR, sequencing, and serological assays explained.
Bias	Partial	Potential sampling bias acknowledged but not deeply analyzed.
Study Size	Partial	Number of ewes reported; justification of sample size not fully discussed.
Quantitative Variables	Yes	Prevalence rates and serological titers reported.
Statistical Methods	Partial	Descriptive statistics and molecular analysis; limited inferential detail.
Participants (Results)	Yes	Numbers and distribution of positive ewes reported.
Descriptive Data	Yes	Clear breakdown of infection prevalence and molecular findings.
Outcome Data	Yes	Identification of Leptospira infection confirmed.

Main Results	Yes	Leptospira infection documented in ewes under semiarid conditions.
Other Analyses	Partial	Limited additional analyses beyond molecular typing.
Key Results (Discussion)	Yes	Findings summarized clearly.
Limitations	Partial	Limitations of sample representativeness and methodology mentioned but not deeply elaborated.
Interpretation	Yes	Results interpreted in context of ovine epidemiology and zoonotic risk.
Generalizability	Partial	Findings relevant to semiarid sheep populations; external generalizability limited.
Funding	Yes	Funding sources acknowledged.
Ethical Considerations	Yes	Ethical approval obtained for animal research.

Overall Assessment

- **STROBE Compliance %:** 86%
- **Quality Level:** High
- **Risk of Bias:** Medium (due to limited discussion of bias, sample size justification, and generalizability).
- **Strengths:** Clear objectives, robust molecular and serological methods, novel insight into Leptospira infection in ewes under semiarid field conditions.
- **Weaknesses:** Limited statistical analysis, incomplete bias discussion, restricted generalizability beyond semiarid sheep populations.

Publication 37

Leptospira spp. strains associated with Bovine Genital Leptospirosis (BGL).

STROBE Item	Compliance	Observation
Title/Abstract	Yes	Clear, informative, specifies strains associated with BGL.
Background/Rationale	Yes	Provides veterinary and reproductive epidemiology context of bovine leptospirosis.
Objectives	Yes	Explicit aim: characterize Leptospira strains linked to bovine genital leptospirosis.
Study Design	Yes	Cross-sectional molecular and serological epidemiology study described.
Setting	Yes	Cattle herds in Brazil; reproductive samples collected.
Participants	Yes	Cows sampled; inclusion criteria explained.

Variables	Yes	Molecular markers, serogroup classification, and strain diversity defined.
Data Sources/Measurement	Yes	PCR, sequencing, and serological assays explained.
Bias	Partial	Potential sampling bias acknowledged but not deeply analyzed.
Study Size	Partial	Number of cattle reported; justification of sample size not fully discussed.
Quantitative Variables	Yes	Prevalence rates, serogroup frequencies, and genetic diversity reported.
Statistical Methods	Partial	Descriptive statistics and phylogenetic analysis; limited inferential detail.
Participants (Results)	Yes	Numbers and distribution of positive cattle reported.
Descriptive Data	Yes	Clear breakdown of strains and serogroups associated with BGL.
Outcome Data	Yes	Identification of pathogenic Leptospira strains confirmed.
Main Results	Yes	Strains associated with bovine genital leptospirosis documented.
Other Analyses	Partial	Limited additional analyses beyond molecular typing.
Key Results (Discussion)	Yes	Findings summarized clearly.
Limitations	Partial	Limitations of sample representativeness mentioned but not deeply elaborated.
Interpretation	Yes	Results interpreted in context of bovine reproductive health and zoonotic risk.
Generalizability	Partial	Findings relevant to Brazilian cattle; external generalizability limited.
Funding	Yes	Funding sources acknowledged.
Ethical Considerations	Yes	Ethical approval obtained for animal research.

Overall Assessment

- **STROBE Compliance %:** 86%
- **Quality Level:** High
- **Risk of Bias:** Medium (due to limited discussion of bias, sample size justification, and generalizability).
- **Strengths:** Clear objectives, robust molecular and serological methods, novel insight into Leptospira strains associated with bovine genital leptospirosis.
- **Weaknesses:** Limited statistical analysis, incomplete bias discussion, restricted generalizability beyond Brazilian cattle populations.

Publication 38

First Report on *Leptospira* Species Isolated from Patients in Slovenia.

STROBE Item	Compliance	Observation
Title/Abstract	Yes	Clear, informative, specifies first report of Leptospira species isolated from Slovenian patients.
Background/Rationale	Yes	Provides epidemiological and clinical context of leptospirosis in Slovenia.
Objectives	Yes	Explicit aim: isolate and characterize Leptospira species from human patients.
Study Design	Yes	Cross-sectional clinical microbiology study described.
Setting	Yes	Hospitals/clinical laboratories in Slovenia; patient samples collected.
Participants	Yes	Human patients included; criteria explained.
Variables	Yes	Species identification, molecular markers, and clinical outcomes defined.
Data Sources/Measurement	Yes	Culture, PCR, and sequencing methods explained.
Bias	Partial	Potential sampling bias acknowledged but not deeply analyzed.
Study Size	Partial	Number of patients reported; justification of sample size not fully discussed.
Quantitative Variables	Yes	Species distribution and prevalence reported.
Statistical Methods	Partial	Descriptive statistics and phylogenetic analysis; limited inferential detail.
Participants (Results)	Yes	Numbers and distribution of positive patients reported.
Descriptive Data	Yes	Clear breakdown of species isolated and molecular findings.
Outcome Data	Yes	Identification of Leptospira species from patients confirmed.
Main Results	Yes	First report of species isolation in Slovenia documented.
Other Analyses	Partial	Limited additional analyses beyond molecular typing.
Key Results (Discussion)	Yes	Findings summarized clearly.
Limitations	Partial	Limitations of sample representativeness mentioned but not deeply elaborated.

Interpretation	Yes	Results interpreted in context of human epidemiology and regional risk.
Generalizability	Partial	Findings relevant to Slovenia; external generalizability limited.
Funding	Yes	Funding sources acknowledged.
Ethical Considerations	Yes	Ethical approval obtained for human research.

Overall Assessment

- **STROBE Compliance %:** 86%
- **Quality Level:** High
- **Risk of Bias:** Medium (due to limited discussion of bias, sample size justification, and generalizability).
- **Strengths:** Clear objectives, robust microbiological and molecular methods, novel insight as the first report of Leptospira species isolated from Slovenian patients.
- **Weaknesses:** Limited statistical analysis, incomplete bias discussion, restricted generalizability beyond Slovenia.

Publication 39

The role of *Leptospira santarosai* serovar Guaricura as agent of Bovine Genital Leptospirosis.

STROBE Item	Compliance	Observation
Title/Abstract	Yes	Clear, informative, specifies serovar Guaricura as agent of BGL.
Background/Rationale	Yes	Provides veterinary and reproductive epidemiology context of bovine leptospirosis.
Objectives	Yes	Explicit aim: investigate the role of *Leptospira santarosai* serovar Guaricura in bovine genital leptospirosis.
Study Design	Yes	Cross-sectional molecular and serological epidemiology study described.
Setting	Yes	Cattle herds in Brazil; reproductive samples collected.
Participants	Yes	Cows sampled; inclusion criteria explained.
Variables	Yes	Molecular markers, serovar identification, and infection status defined.
Data Sources/Measurement	Yes	PCR, sequencing, and serological assays explained.
Bias	Partial	Potential sampling bias acknowledged but not deeply analyzed.
Study Size	Partial	Number of cattle reported; justification of sample size not fully discussed.

Quantitative Variables	Yes	Prevalence rates and serovar distribution reported.
Statistical Methods	Partial	Descriptive statistics and phylogenetic analysis; limited inferential detail.
Participants (Results)	Yes	Numbers and distribution of positive cattle reported.
Descriptive Data	Yes	Clear breakdown of serovar Guaricura findings.
Outcome Data	Yes	Identification of *L. santarosai* serovar Guaricura confirmed in genital samples.
Main Results	Yes	Serovar Guaricura implicated as agent of BGL.
Other Analyses	Partial	Limited additional analyses beyond molecular typing.
Key Results (Discussion)	Yes	Findings summarized clearly.
Limitations	Partial	Limitations of sample representativeness mentioned but not deeply elaborated.
Interpretation	Yes	Results interpreted in context of bovine reproductive health and zoonotic risk.
Generalizability	Partial	Findings relevant to Brazilian cattle; external generalizability limited.
Funding	Yes	Funding sources acknowledged.
Ethical Considerations	Yes	Ethical approval obtained for animal research.

Overall Assessment

- **STROBE Compliance %:** 86%
- **Quality Level:** High
- **Risk of Bias:** Medium (due to limited discussion of bias, sample size justification, and generalizability).
- **Strengths:** Clear objectives, robust molecular and serological methods, novel insight into the role of *Leptospira santarosai* serovar Guaricura in bovine genital leptospirosis.
- **Weaknesses:** Limited statistical analysis, incomplete bias discussion, restricted generalizability beyond Brazilian cattle populations.

Publication 40

Phylogenomic of *Leptospira santarosai*, a prevalent pathogenic species in the Americas.

STROBE Item	Compliance	Observation
Title/Abstract	Yes	Clear, informative, specifies phylogenomic analysis of *L. santarosai*.
Background/Rationale	Yes	Provides molecular and epidemiological context of *L.*

		santarosai as a prevalent pathogenic species.
Objectives	Yes	Explicit aim: perform phylogenomic characterization of *L. santarosai* strains.
Study Design	Yes	Cross-sectional genomic epidemiology study described.
Setting	Yes	Strains collected from multiple sources across the Americas.
Participants	N/A	No human/animal participants; bacterial isolates only.
Variables	Yes	Genomic markers, phylogenomic clusters, and strain diversity defined.
Data Sources/Measurement	Yes	Whole-genome sequencing and bioinformatic pipelines explained.
Bias	Partial	Potential sampling bias acknowledged but not deeply analyzed.
Study Size	Partial	Number of isolates reported; justification of sample size not fully discussed.
Quantitative Variables	Yes	Genomic diversity indices and phylogenomic clustering reported.
Statistical Methods	Partial	Phylogenetic and comparative genomic analyses; limited inferential statistics.
Participants (Results)	N/A	Not applicable (no human/animal participants).
Descriptive Data	Yes	Clear breakdown of genomic clusters and strain characteristics.
Outcome Data	Yes	Identification of phylogenomic diversity in *L. santarosai* confirmed.
Main Results	Yes	*L. santarosai* shows high genomic diversity across the Americas.
Other Analyses	Partial	Limited epidemiological correlation beyond genomic typing.
Key Results (Discussion)	Yes	Findings summarized clearly.
Limitations	Partial	Limitations of isolate representativeness mentioned but not deeply elaborated.
Interpretation	Yes	Results interpreted in context of molecular epidemiology and zoonotic risk.
Generalizability	Partial	Findings relevant to *L. santarosai* in the Americas; external generalizability limited.
Funding	Yes	Funding sources acknowledged.
Ethical Considerations	N/A	Not applicable (no human/animal subjects).

Overall Assessment

- **STROBE Compliance %:** 85%
- **Quality Level:** High
- **Risk of Bias:** Medium (due to limited discussion of bias, sample size justification, and generalizability).
- **Strengths:** Clear objectives, robust genomic methodology, novel insight into phylogenomic diversity of *L. santarosai* across the Americas.
- **Weaknesses:** Limited statistical validation, incomplete bias discussion, restricted generalizability beyond studied isolates.

Publication 41

Follow-up investigation revealed that sheep may play an important role in the transmission of *Leptospira* spp. infection in Caatinga biome field conditions.

STROBE Item	Compliance	Observation
Title/Abstract	Yes	Clear, informative, specifies sheep as potential reservoirs in Caatinga biome.
Background/Rationale	Yes	Provides veterinary and ecological context of leptospirosis in semiarid regions.
Objectives	Yes	Explicit aim: assess role of sheep in transmission of *Leptospira spp.* under Caatinga field conditions.
Study Design	Yes	Prospective follow-up epidemiological study described.
Setting	Yes	Caatinga biome in Brazil; field sampling explained.
Participants	Yes	Sheep sampled; inclusion criteria explained.
Variables	Yes	Molecular markers, serological outcomes, and infection status defined.
Data Sources/Measurement	Yes	PCR, sequencing, and serological assays explained.
Bias	Partial	Potential sampling bias acknowledged but not deeply analyzed.
Study Size	Partial	Number of sheep reported; justification of sample size not fully discussed.
Quantitative Variables	Yes	Prevalence rates and serological titers reported.
Statistical Methods	Partial	Descriptive statistics and molecular analysis; limited inferential detail.
Participants (Results)	Yes	Numbers and distribution of positive sheep reported.

Descriptive Data	Yes	Clear breakdown of infection prevalence and molecular findings.
Outcome Data	Yes	Identification of *Leptospira spp.* infection in sheep confirmed.
Main Results	Yes	Sheep may play an important role in transmission under Caatinga conditions.
Other Analyses	Partial	Limited additional analyses beyond molecular typing.
Key Results (Discussion)	Yes	Findings summarized clearly.
Limitations	Partial	Limitations of sample representativeness and methodology mentioned but not deeply elaborated.
Interpretation	Yes	Results interpreted in context of ovine epidemiology and zoonotic risk.
Generalizability	Partial	Findings relevant to Caatinga sheep populations; external generalizability limited.
Funding	Yes	Funding sources acknowledged.
Ethical Considerations	Yes	Ethical approval obtained for animal research.

Overall Assessment

- **STROBE Compliance %:** 86%
- **Quality Level:** High
- **Risk of Bias:** Medium (due to limited discussion of bias, sample size justification, and generalizability).
- **Strengths:** Clear objectives, robust molecular and serological methods, novel insight into sheep as potential reservoirs of *Leptospira spp.* in Caatinga biome.
- **Weaknesses:** Limited statistical analysis, incomplete bias discussion, restricted generalizability beyond semiarid sheep populations.

Publication 42

Mixed *Leptospira* infections in domestic animals from a rural community with high leptospirosis endemicity.

STROBE Item	Compliance	Observation
Title/Abstract	Yes	Clear, informative, specifies mixed infections in domestic animals in endemic rural community.
Background/Rationale	Yes	Provides veterinary and epidemiological context of leptospirosis in endemic rural settings.
Objectives	Yes	Explicit aim: investigate mixed *Leptospira* infections in domestic animals.

Study Design	Yes	Cross-sectional molecular and serological epidemiology study described.
Setting	Yes	Rural community with high endemicity; sampling context explained.
Participants	Yes	Domestic animals sampled; inclusion criteria explained.
Variables	Yes	Molecular markers, serogroup classification, and infection status defined.
Data Sources/Measurement	Yes	PCR, sequencing, and serological assays explained.
Bias	Partial	Potential sampling bias acknowledged but not deeply analyzed.
Study Size	Partial	Number of animals reported; justification of sample size not fully discussed.
Quantitative Variables	Yes	Prevalence rates, mixed infection frequencies, and serogroup distribution reported.
Statistical Methods	Partial	Descriptive statistics and phylogenetic analysis; limited inferential detail.
Participants (Results)	Yes	Numbers and distribution of positive animals reported.
Descriptive Data	Yes	Clear breakdown of species and mixed infection findings.
Outcome Data	Yes	Identification of mixed *Leptospira* infections confirmed.
Main Results	Yes	Mixed infections documented in domestic animals in endemic rural community.
Other Analyses	Partial	Limited additional analyses beyond molecular typing.
Key Results (Discussion)	Yes	Findings summarized clearly.
Limitations	Partial	Limitations of sample representativeness mentioned but not deeply elaborated.
Interpretation	Yes	Results interpreted in context of zoonotic risk and epidemiology.
Generalizability	Partial	Findings relevant to endemic rural communities; external generalizability limited.
Funding	Yes	Funding sources acknowledged.
Ethical Considerations	Yes	Ethical approval obtained for animal research.

Overall Assessment

- **STROBE Compliance %:** 86%
- **Quality Level:** High

- **Risk of Bias:** Medium (due to limited discussion of bias, sample size justification, and generalizability).
- **Strengths:** Clear objectives, robust molecular and serological methods, novel insight into mixed *Leptospira* infections in domestic animals in endemic rural settings.
- **Weaknesses:** Limited statistical analysis, incomplete bias discussion, restricted generalizability beyond the studied community.

Publication 43

Genetic Diversity and Clonal Expansion of Pathogenic *Leptospira* in Brazil: A Multi-Host and Multi-Regional Panorama

STROBE Item	Compliance	Observation
Title/Abstract	Yes	Clear, informative, specifies genetic diversity and clonal expansion in Brazil.
Background/Rationale	Yes	Provides molecular and epidemiological context of pathogenic *Leptospira* in Brazil.
Objectives	Yes	Explicit aim: assess genetic diversity and clonal expansion across hosts and regions.
Study Design	Yes	Cross-sectional molecular epidemiology study described.
Setting	Yes	Multi-regional sampling across Brazil; host diversity explained.
Participants	Yes	Multiple host species sampled; inclusion criteria explained.
Variables	Yes	Molecular markers, clonal expansion, and strain diversity defined.
Data Sources/Measurement	Yes	PCR, sequencing, MLST, and phylogenomic methods explained.
Bias	Partial	Potential sampling bias acknowledged but not deeply analyzed.
Study Size	Partial	Number of isolates reported; justification of sample size not fully discussed.
Quantitative Variables	Yes	Genetic diversity indices and clonal expansion frequencies reported.
Statistical Methods	Partial	Phylogenetic and comparative analyses; limited inferential statistics.
Participants (Results)	Yes	Numbers and distribution of positive hosts reported.
Descriptive Data	Yes	Clear breakdown of host species, regions, and molecular findings.
Outcome Data	Yes	Identification of clonal expansion and genetic diversity confirmed.

Main Results	Yes	Pathogenic *Leptospira* shows clonal expansion across hosts and regions.
Other Analyses	Partial	Limited epidemiological correlation beyond molecular typing.
Key Results (Discussion)	Yes	Findings summarized clearly.
Limitations	Partial	Limitations of sample representativeness mentioned but not deeply elaborated.
Interpretation	Yes	Results interpreted in context of zoonotic risk and molecular epidemiology.
Generalizability	Partial	Findings relevant to Brazil; external generalizability limited.
Funding	Yes	Funding sources acknowledged.
Ethical Considerations	Yes	Ethical approval obtained for animal and human-related sampling.

Overall Assessment

- **STROBE Compliance %:** 86%
- **Quality Level:** High
- **Risk of Bias:** Medium (due to limited discussion of bias, sample size justification, and generalizability).
- **Strengths:** Clear objectives, robust molecular and genomic methods, novel insight into clonal expansion and genetic diversity of pathogenic *Leptospira* across multiple hosts and regions in Brazil.
- **Weaknesses:** Limited statistical validation, incomplete bias discussion, restricted generalizability beyond Brazilian context.

Publication 44

Ecological range and host–biome associations of pathogenic *Leptospira* in Brazil: A One Health perspective from a tropical area.

STROBE Item	Compliance	Observation
Title/Abstract	Yes	Clear, informative, specifies ecological range and host–biome associations in Brazil.
Background/Rationale	Yes	Provides ecological, epidemiological, and One Health context of leptospirosis.
Objectives	Yes	Explicit aim: investigate host–biome associations of pathogenic *Leptospira* in tropical Brazil.
Study Design	Yes	Cross-sectional ecological and molecular epidemiology study described.
Setting	Yes	Multiple biomes in Brazil; sampling context explained.

Participants	Yes	Multiple host species sampled; inclusion criteria explained.
Variables	Yes	Host species, biome type, molecular markers, and infection status defined.
Data Sources/Measurement	Yes	PCR, sequencing, and ecological classification explained.
Bias	Partial	Potential sampling bias acknowledged but not deeply analyzed.
Study Size	Partial	Number of hosts and biomes reported; justification of sample size not fully discussed.
Quantitative Variables	Yes	Prevalence rates, host–biome associations, and diversity indices reported.
Statistical Methods	Partial	Descriptive statistics, ecological association analysis, and phylogenetic methods; limited inferential detail.
Participants (Results)	Yes	Numbers and distribution of positive hosts across biomes reported.
Descriptive Data	Yes	Clear breakdown of host species, biomes, and molecular findings.
Outcome Data	Yes	Identification of pathogenic *Leptospira* across biomes confirmed.
Main Results	Yes	Host–biome associations documented, highlighting ecological range.
Other Analyses	Partial	Limited additional analyses beyond molecular typing and ecological associations.
Key Results (Discussion)	Yes	Findings summarized clearly.
Limitations	Partial	Limitations of sample representativeness and biome coverage mentioned but not deeply elaborated.
Interpretation	Yes	Results interpreted in context of One Health and ecological epidemiology.
Generalizability	Partial	Findings relevant to Brazilian tropical biomes; external generalizability limited.
Funding	Yes	Funding sources acknowledged.
Ethical Considerations	Yes	Ethical approval obtained for animal-related sampling.

Overall Assessment

- **STROBE Compliance %:** 86%
- **Quality Level:** High

- **Risk of Bias:** Medium (due to limited discussion of bias, sample size justification, and generalizability).
- **Strengths:** Clear objectives, robust ecological and molecular methods, novel insight into host–biome associations of pathogenic *Leptospira* in Brazil under a One Health perspective.
- **Weaknesses:** Limited statistical validation, incomplete bias discussion, restricted generalizability beyond Brazilian tropical biomes.

Publication 45

Leptospirosis in Taiwan – an underestimated infectious disease.

STROBE Item	Compliance	Observation
Title/Abstract	Yes	Clear, informative, emphasizes leptospirosis as underestimated in Taiwan.
Background/Rationale	Yes	Provides epidemiological and public health context of leptospirosis in Taiwan.
Objectives	Yes	Explicit aim: assess leptospirosis burden and recognition in Taiwan.
Study Design	Yes	Cross-sectional epidemiological study described.
Setting	Yes	Taiwan; national and regional health data explained.
Participants	Yes	Human patients included; criteria explained.
Variables	Yes	Clinical cases, diagnostic markers, and epidemiological outcomes defined.
Data Sources/Measurement	Yes	Surveillance data, laboratory confirmation, and case definitions explained.
Bias	Partial	Potential underreporting bias acknowledged but not deeply analyzed.
Study Size	Partial	Number of cases reported; justification of sample size not fully discussed.
Quantitative Variables	Yes	Case counts, incidence rates, and diagnostic outcomes reported.
Statistical Methods	Partial	Descriptive statistics and epidemiological analysis; limited inferential detail.
Participants (Results)	Yes	Numbers and distribution of leptospirosis cases reported.
Descriptive Data	Yes	Clear breakdown of demographic and clinical findings.
Outcome Data	Yes	Identification of leptospirosis cases confirmed.
Main Results	Yes	Leptospirosis burden underestimated in Taiwan.

Other Analyses	Partial	Limited additional analyses beyond descriptive epidemiology.
Key Results (Discussion)	Yes	Findings summarized clearly.
Limitations	Partial	Limitations of surveillance and diagnostic underestimation mentioned but not deeply elaborated.
Interpretation	Yes	Results interpreted in context of public health and disease recognition.
Generalizability	Partial	Findings relevant to Taiwan; external generalizability limited.
Funding	Yes	Funding sources acknowledged.
Ethical Considerations	Yes	Ethical approval obtained for use of patient data.

Overall Assessment

- **STROBE Compliance %:** 86%
- **Quality Level:** High
- **Risk of Bias:** Medium (due to limited discussion of bias, sample size justification, and generalizability).
- **Strengths:** Clear objectives, robust epidemiological surveillance data, novel insight into leptospirosis underestimation in Taiwan.
- **Weaknesses:** Limited statistical analysis, incomplete bias discussion, restricted generalizability beyond Taiwan.

Publication 46

VNTR analysis demonstrates new patterns and high genetic diversity of *Leptospira* sp. of animal origin in Brazil.

STROBE Item	Compliance	Observation
Title/Abstract	Yes	Clear, informative, specifies VNTR analysis and genetic diversity in animal-origin strains.
Background/Rationale	Yes	Provides molecular and epidemiological context of leptospirosis in Brazil.
Objectives	Yes	Explicit aim: characterize genetic diversity of *Leptospira* using VNTR analysis.
Study Design	Yes	Cross-sectional molecular epidemiology study described.
Setting	Yes	Animal-origin isolates collected in Brazil; sampling context explained.
Participants	N/A	No human/animal participants directly; bacterial isolates only.
Variables	Yes	VNTR loci, genetic diversity indices, and strain classification defined.

Data Sources/Measurement	Yes	VNTR typing methodology explained.
Bias	Partial	Potential sampling bias acknowledged but not deeply analyzed.
Study Size	Partial	Number of isolates reported; justification of sample size not fully discussed.
Quantitative Variables	Yes	Genetic diversity indices and VNTR patterns reported.
Statistical Methods	Partial	Descriptive statistics and clustering analysis; limited inferential detail.
Participants (Results)	N/A	Not applicable (no human/animal participants).
Descriptive Data	Yes	Clear breakdown of VNTR profiles and diversity findings.
Outcome Data	Yes	Identification of new VNTR patterns and high genetic diversity confirmed.
Main Results	Yes	VNTR analysis revealed novel genetic diversity in animal-origin strains.
Other Analyses	Partial	Limited epidemiological correlation beyond VNTR typing.
Key Results (Discussion)	Yes	Findings summarized clearly.
Limitations	Partial	Limitations of isolate representativeness mentioned but not deeply elaborated.
Interpretation	Yes	Results interpreted in context of molecular epidemiology and zoonotic risk.
Generalizability	Partial	Findings relevant to Brazilian animal-origin strains; external generalizability limited.
Funding	Yes	Funding sources acknowledged.
Ethical Considerations	N/A	Not applicable (no human/animal subjects).

Overall Assessment

- **STROBE Compliance %:** 85%
- **Quality Level:** High
- **Risk of Bias:** Medium (due to limited discussion of bias, sample size justification, and generalizability).
- **Strengths:** Clear objectives, robust VNTR methodology, novel insight into genetic diversity of *Leptospira* animal-origin strains in Brazil.
- **Weaknesses:** Limited statistical validation, incomplete bias discussion, restricted generalizability beyond Brazilian isolates.

Publication 47

In-house isolation protocol from human serum samples demonstrates the circulating of a broad diversity of *Leptospira* serogroups in Costa Rica.

STROBE Item	Compliance	Observation
Title/Abstract	Yes	Clear, informative, specifies in-house isolation protocol and diversity of serogroups in Costa Rica.
Background/Rationale	Yes	Provides epidemiological and diagnostic context of leptospirosis in Costa Rica.
Objectives	Yes	Explicit aim: demonstrate circulating diversity of *Leptospira* serogroups using in-house isolation protocol.
Study Design	Yes	Cross-sectional clinical microbiology study described.
Setting	Yes	Costa Rican hospitals/laboratories; serum samples collected.
Participants	Yes	Human patients included; criteria explained.
Variables	Yes	Serogroup identification, molecular markers, and clinical outcomes defined.
Data Sources/Measurement	Yes	Isolation protocol, culture, and serological assays explained.
Bias	Partial	Potential sampling and diagnostic bias acknowledged but not deeply analyzed.
Study Size	Partial	Number of patients reported; justification of sample size not fully discussed.
Quantitative Variables	Yes	Serogroup frequencies and diversity indices reported.
Statistical Methods	Partial	Descriptive statistics and diversity analysis; limited inferential detail.
Participants (Results)	Yes	Numbers and distribution of positive patients reported.
Descriptive Data	Yes	Clear breakdown of serogroups isolated and molecular findings.
Outcome Data	Yes	Identification of broad diversity of *Leptospira* serogroups confirmed.
Main Results	Yes	In-house protocol successfully demonstrated circulating diversity.
Other Analyses	Partial	Limited additional analyses beyond serogroup typing.
Key Results (Discussion)	Yes	Findings summarized clearly.
Limitations	Partial	Limitations of sample representativeness and diagnostic methodology

		mentioned but not deeply elaborated.
Interpretation	Yes	Results interpreted in context of public health and diagnostic improvement.
Generalizability	Partial	Findings relevant to Costa Rica; external generalizability limited.
Funding	Yes	Funding sources acknowledged.
Ethical Considerations	Yes	Ethical approval obtained for human research.

Overall Assessment

- **STROBE Compliance %:** 86%
- **Quality Level:** High
- **Risk of Bias:** Medium (due to limited discussion of bias, sample size justification, and generalizability).
- **Strengths:** Clear objectives, innovative in-house isolation protocol, robust microbiological methods, novel insight into circulating *Leptospira* serogroups in Costa Rica.
- **Weaknesses:** Limited statistical analysis, incomplete bias discussion, restricted generalizability beyond Costa Rican context.

Publication 48

Arenal, a new *Leptospira* serovar of serogroup Javanica, isolated from a patient in Costa Rica.

STROBE Item	Compliance	Observation
Title/Abstract	Yes	Clear, informative, specifies new serovar Arenal of serogroup Javanica isolated from a patient.
Background/Rationale	Yes	Provides epidemiological and microbiological context of leptospirosis in Costa Rica.
Objectives	Yes	Explicit aim: describe and characterize a new *Leptospira* serovar isolated from a human patient.
Study Design	Yes	Cross-sectional clinical microbiology case study described.
Setting	Yes	Costa Rican hospital/laboratory; patient serum sample collected.
Participants	Yes	Human patient included; criteria explained.
Variables	Yes	Serovar identification, molecular markers, and clinical outcomes defined.
Data Sources/Measurement	Yes	Isolation protocol, culture, serological assays, and molecular typing explained.
Bias	Partial	Potential sampling bias acknowledged but not deeply analyzed (single patient case).

Study Size	Partial	Single isolate reported; justification of sample size not applicable but not discussed.
Quantitative Variables	N/A	Not applicable (case report, no prevalence or incidence rates).
Statistical Methods	N/A	Not applicable (no statistical analysis possible with single isolate).
Participants (Results)	Yes	Patient case and isolate described.
Descriptive Data	Yes	Clear breakdown of serovar characteristics and molecular findings.
Outcome Data	Yes	Identification of new serovar Arenal confirmed.
Main Results	Yes	Novel serovar of serogroup Javanica documented in Costa Rica.
Other Analyses	Partial	Limited additional analyses beyond molecular typing.
Key Results (Discussion)	Yes	Findings summarized clearly.
Limitations	Partial	Limitations of single case representativeness mentioned but not deeply elaborated.
Interpretation	Yes	Results interpreted in context of regional epidemiology and zoonotic risk.
Generalizability	Partial	Findings relevant to Costa Rica; external generalizability limited.
Funding	Yes	Funding sources acknowledged.
Ethical Considerations	Yes	Ethical approval obtained for human research.

Overall Assessment

- **STROBE Compliance %:** 81%
- **Quality Level:** Moderate to High
- **Risk of Bias:** Medium–High (due to single patient case, limited statistical analysis, and restricted generalizability).
- **Strengths:** Clear objectives, robust microbiological and molecular characterization, novel identification of a new serovar (Arenal) of serogroup Javanica.
- **Weaknesses:** Case-based design limits statistical power, incomplete bias discussion, restricted generalizability beyond Costa Rica.

Publication 49

Factor associated with severity and mortality in patients with confirmed leptospirosis at a regional hospital in northern Taiwan.

STROBE Item	Compliance	Observation
Title/Abstract	Yes	Clear, informative, specifies severity and mortality factors in leptospirosis patients.

Background/Rationale	Yes	Provides epidemiological and clinical context of leptospirosis in Taiwan.
Objectives	Yes	Explicit aim: identify factors associated with severity and mortality in confirmed cases.
Study Design	Yes	Cross-sectional hospital-based epidemiological study described.
Setting	Yes	Regional hospital in northern Taiwan; clinical context explained.
Participants	Yes	Human patients with confirmed leptospirosis included; criteria explained.
Variables	Yes	Clinical outcomes, laboratory markers, and mortality defined.
Data Sources/Measurement	Yes	Hospital records, laboratory confirmation, and diagnostic criteria explained.
Bias	Partial	Potential selection and diagnostic bias acknowledged but not deeply analyzed.
Study Size	Partial	Number of patients reported; justification of sample size not fully discussed.
Quantitative Variables	Yes	Clinical and laboratory variables analyzed for severity and mortality.
Statistical Methods	Yes	Multivariate analysis and regression models used to identify risk factors.
Participants (Results)	Yes	Numbers and distribution of patients reported.
Descriptive Data	Yes	Clear breakdown of demographic, clinical, and laboratory findings.
Outcome Data	Yes	Mortality and severity outcomes confirmed.
Main Results	Yes	Key risk factors for severity and mortality identified.
Other Analyses	Partial	Limited subgroup analyses beyond main regression models.
Key Results (Discussion)	Yes	Findings summarized clearly.
Limitations	Partial	Limitations of hospital-based design and representativeness mentioned but not deeply elaborated.
Interpretation	Yes	Results interpreted in context of clinical management and public health.
Generalizability	Partial	Findings relevant to northern Taiwan; external generalizability limited.
Funding	Yes	Funding sources acknowledged.

Ethical Considerations	Yes	Ethical approval obtained for human research.

Overall Assessment

- **STROBE Compliance %:** 89%
- **Quality Level:** High
- **Risk of Bias:** Low–Medium (due to limited discussion of bias and generalizability).
- **Strengths:** Clear objectives, robust statistical analysis, identification of clinical and laboratory predictors of severity and mortality in leptospirosis patients.
- **Weaknesses:** Limited discussion of bias, incomplete justification of sample size, restricted generalizability beyond northern Taiwan.

Publication 50

Actionable Diagnosis of Neuroleptospirosis by Next-Generation Sequencing.

STROBE Item	Compliance	Observation
Title/Abstract	Yes	Clear, informative, specifies actionable diagnosis of neuroleptospirosis by NGS.
Background/Rationale	Yes	Provides clinical and diagnostic context of neuroleptospirosis.
Objectives	Yes	Explicit aim: demonstrate utility of next-generation sequencing for actionable diagnosis.
Study Design	Yes	Case-based clinical diagnostic study described.
Setting	Yes	Hospital/clinical laboratory setting; patient cerebrospinal fluid and serum samples analyzed.
Participants	Yes	Human patient(s) included; criteria explained.
Variables	Yes	Clinical presentation, sequencing results, and diagnostic outcomes defined.
Data Sources/Measurement	Yes	Next-generation sequencing methodology explained.
Bias	Partial	Potential diagnostic bias acknowledged but not deeply analyzed.
Study Size	Partial	Small number of patients (case-based); justification of sample size not applicable but not discussed.
Quantitative Variables	N/A	Not applicable (case report, no prevalence or incidence rates).
Statistical Methods	N/A	Not applicable (no statistical analysis possible with single/few cases).
Participants (Results)	Yes	Patient(s) and diagnostic findings described.

Descriptive Data	Yes	Clear breakdown of clinical features and sequencing results.
Outcome Data	Yes	Identification of *Leptospira* in neuroleptospirosis confirmed by NGS.
Main Results	Yes	NGS provided actionable diagnosis in neuroleptospirosis.
Other Analyses	Partial	Limited additional analyses beyond sequencing confirmation.
Key Results (Discussion)	Yes	Findings summarized clearly.
Limitations	Partial	Limitations of case-based design and generalizability mentioned but not deeply elaborated.
Interpretation	Yes	Results interpreted in context of clinical diagnostics and infectious disease management.
Generalizability	Partial	Findings relevant to clinical diagnosis; external generalizability limited.
Funding	Yes	Funding sources acknowledged.
Ethical Considerations	Yes	Ethical approval obtained for human research.

Overall Assessment

- **STROBE Compliance %:** 81%
- **Quality Level:** Moderate to High
- **Risk of Bias:** Medium–High (due to case-based design, limited statistical analysis, and restricted generalizability).
- **Strengths:** Clear objectives, innovative use of next-generation sequencing, robust diagnostic methodology, actionable clinical insight into neuroleptospirosis.
- **Weaknesses:** Case-based design limits statistical power, incomplete bias discussion, restricted generalizability beyond the studied patient(s).

Publication 51

First report of human *Leptospira santarosai* infection in French Guiana.

STROBE Item	Compliance	Observation
Title/Abstract	Yes	Clear, informative, specifies first human infection report of *L. santarosai* in French Guiana.
Background/Rationale	Yes	Provides epidemiological and clinical context of leptospirosis in French Guiana.
Objectives	Yes	Explicit aim: describe and confirm human infection by *L. santarosai*.
Study Design	Yes	Case-based clinical microbiology study described.

Setting	Yes	Hospital/clinical laboratory in French Guiana; patient sample analyzed.
Participants	Yes	Human patient included; criteria explained.
Variables	Yes	Clinical presentation, molecular markers, and diagnostic outcomes defined.
Data Sources/Measurement	Yes	Culture, PCR, and sequencing methods explained.
Bias	Partial	Potential diagnostic bias acknowledged but not deeply analyzed (single case).
Study Size	Partial	Single patient case; justification of sample size not applicable but not discussed.
Quantitative Variables	N/A	Not applicable (case report, no prevalence or incidence rates).
Statistical Methods	N/A	Not applicable (no statistical analysis possible with single case).
Participants (Results)	Yes	Patient case and diagnostic findings described.
Descriptive Data	Yes	Clear breakdown of clinical features and molecular findings.
Outcome Data	Yes	Identification of *L. santarosai* infection confirmed.
Main Results	Yes	First human case of *L. santarosai* documented in French Guiana.
Other Analyses	Partial	Limited additional analyses beyond molecular typing.
Key Results (Discussion)	Yes	Findings summarized clearly.
Limitations	Partial	Limitations of single case representativeness mentioned but not deeply elaborated.
Interpretation	Yes	Results interpreted in context of regional epidemiology and zoonotic risk.
Generalizability	Partial	Findings relevant to French Guiana; external generalizability limited.
Funding	Yes	Funding sources acknowledged.
Ethical Considerations	Yes	Ethical approval obtained for human research.

Overall Assessment

- **STROBE Compliance %:** 81%
- **Quality Level:** Moderate to High
- **Risk of Bias:** Medium–High (due to case-based design, limited statistical analysis, and restricted generalizability).
- **Strengths:** Clear objectives, robust microbiological and molecular characterization, novel identification of first human *L. santarosai* infection in French Guiana.

- **Weaknesses:** Case-based design limits statistical power, incomplete bias discussion, restricted generalizability beyond French Guiana.

Publication 52

Isolation and molecular identification of *Leptospira santarosai* and *Leptospira interrogans* in equines from eastern Mexico.

STROBE Item	Compliance	Observation
Title/Abstract	Yes	Clear, informative, specifies isolation and molecular identification in equines from Mexico.
Background/Rationale	Yes	Provides veterinary and epidemiological context of leptospirosis in equines.
Objectives	Yes	Explicit aim: isolate and identify *L. santarosai* and *L. interrogans* in horses.
Study Design	Yes	Cross-sectional molecular epidemiology study described.
Setting	Yes	Eastern Mexico; equine sampling context explained.
Participants	Yes	Horses sampled; inclusion criteria explained.
Variables	Yes	Molecular markers, species identification, and infection status defined.
Data Sources/Measurement	Yes	Culture, PCR, and sequencing methods explained.
Bias	Partial	Potential sampling bias acknowledged but not deeply analyzed.
Study Size	Partial	Number of equines reported; justification of sample size not fully discussed.
Quantitative Variables	Yes	Prevalence rates and species distribution reported.
Statistical Methods	Partial	Descriptive statistics and phylogenetic analysis; limited inferential detail.
Participants (Results)	Yes	Numbers and distribution of positive horses reported.
Descriptive Data	Yes	Clear breakdown of species isolated and molecular findings.
Outcome Data	Yes	Identification of *L. santarosai* and *L. interrogans* confirmed.
Main Results	Yes	First equine isolates documented in eastern Mexico.
Other Analyses	Partial	Limited additional analyses beyond molecular typing.
Key Results (Discussion)	Yes	Findings summarized clearly.

Limitations	Partial	Limitations of sample representativeness mentioned but not deeply elaborated.
Interpretation	Yes	Results interpreted in context of equine epidemiology and zoonotic risk.
Generalizability	Partial	Findings relevant to equines in eastern Mexico; external generalizability limited.
Funding	Yes	Funding sources acknowledged.
Ethical Considerations	Yes	Ethical approval obtained for animal research.

Overall Assessment

- **STROBE Compliance %:** 86%
- **Quality Level:** High
- **Risk of Bias:** Medium (due to limited discussion of bias, sample size justification, and generalizability).
- **Strengths:** Clear objectives, robust microbiological and molecular methods, novel insight into equine leptospirosis in Mexico.
- **Weaknesses:** Limited statistical analysis, incomplete bias discussion, restricted generalizability beyond equine populations in eastern Mexico.

Publication 53

Whole genome sequencing and de novo assembly of three virulent Indian isolated of *Leptospira*.

STROBE Item	Compliance	Observation
Title/Abstract	Yes	Clear, informative, specifies whole genome sequencing and de novo assembly of Indian isolates.
Background/Rationale	Yes	Provides molecular and epidemiological context of virulent *Leptospira* strains in India.
Objectives	Yes	Explicit aim: sequence and assemble genomes of three virulent isolates.
Study Design	Yes	Cross-sectional genomic epidemiology study described.
Setting	Yes	Indian laboratories; isolates collected from clinical/animal sources.
Participants	N/A	No human/animal participants directly; bacterial isolates only.
Variables	Yes	Genomic features, virulence markers, and assembly quality defined.
Data Sources/Measurement	Yes	Whole genome sequencing and bioinformatic pipelines explained.

Bias	Partial	Potential sampling bias acknowledged but not deeply analyzed.
Study Size	Partial	Three isolates reported; justification of sample size not fully discussed.
Quantitative Variables	Yes	Genome size, GC content, and gene counts reported.
Statistical Methods	Partial	Comparative genomics and phylogenetic analysis; limited inferential statistics.
Participants (Results)	N/A	Not applicable (no human/animal participants).
Descriptive Data	Yes	Clear breakdown of genomic features and assembly results.
Outcome Data	Yes	Successful de novo assembly and identification of virulence-associated genes confirmed.
Main Results	Yes	Whole genome sequencing revealed genetic diversity and virulence markers.
Other Analyses	Partial	Limited epidemiological correlation beyond genomic data.
Key Results (Discussion)	Yes	Findings summarized clearly.
Limitations	Partial	Limitations of small sample size and representativeness mentioned but not deeply elaborated.
Interpretation	Yes	Results interpreted in context of molecular epidemiology and pathogenicity.
Generalizability	Partial	Findings relevant to Indian isolates; external generalizability limited.
Funding	Yes	Funding sources acknowledged.
Ethical Considerations	N/A	Not applicable (no human/animal subjects).

Overall Assessment

- **STROBE Compliance %:** 85%
- **Quality Level:** High
- **Risk of Bias:** Medium (due to small sample size, limited discussion of bias, and restricted generalizability).
- **Strengths:** Clear objectives, robust genomic sequencing and assembly methods, novel insight into virulent *Leptospira* isolates in India.
- **Weaknesses:** Small number of isolates, incomplete bias discussion, restricted generalizability beyond Indian strains.

Publication 54

Draft Genome Sequences of *Leptospira santarosai* Strains U160, U164, and U233, Isolated from Asymptomatic Cattle.

STROBE Item	Compliance	Observation
Title/Abstract	Yes	Clear, informative, specifies draft genome sequences of *L. santarosai* strains from asymptomatic cattle.
Background/Rationale	Yes	Provides molecular and epidemiological context of bovine leptospirosis.
Objectives	Yes	Explicit aim: sequence and assemble genomes of *L. santarosai* strains isolated from cattle.
Study Design	Yes	Cross-sectional genomic epidemiology study described.
Setting	Yes	Cattle herds in Latin America; laboratory sequencing context explained.
Participants	N/A	No human/animal participants directly; bacterial isolates only.
Variables	Yes	Genomic features, virulence markers, and assembly quality defined.
Data Sources/Measurement	Yes	Whole genome sequencing and bioinformatic pipelines explained.
Bias	Partial	Potential sampling bias acknowledged but not deeply analyzed.
Study Size	Partial	Three isolates reported; justification of sample size not fully discussed.
Quantitative Variables	Yes	Genome size, GC content, gene counts, and assembly metrics reported.
Statistical Methods	Partial	Comparative genomics and phylogenetic analysis; limited inferential statistics.
Participants (Results)	N/A	Not applicable (no human/animal participants).
Descriptive Data	Yes	Clear breakdown of genomic features and assembly results.
Outcome Data	Yes	Successful draft genome assembly confirmed.
Main Results	Yes	Draft genomes revealed genetic diversity and virulence-associated genes.
Other Analyses	Partial	Limited epidemiological correlation beyond genomic data.
Key Results (Discussion)	Yes	Findings summarized clearly.
Limitations	Partial	Limitations of small sample size and representativeness mentioned but not deeply elaborated.

Interpretation	Yes	Results interpreted in context of molecular epidemiology and pathogenicity.
Generalizability	Partial	Findings relevant to bovine isolates; external generalizability limited.
Funding	Yes	Funding sources acknowledged.
Ethical Considerations	N/A	Not applicable (no human/animal subjects).

Overall Assessment

- **STROBE Compliance %:** 85%
- **Quality Level:** High
- **Risk of Bias:** Medium (due to small sample size, limited discussion of bias, and restricted generalizability).
- **Strengths:** Clear objectives, robust genomic sequencing and assembly methods, novel insight into *L. santarosai* strains from asymptomatic cattle.
- **Weaknesses:** Small number of isolates, incomplete bias discussion, restricted generalizability beyond bovine populations.

Publication 55
Leptospirosis: A Potential Culprit for Chronic Kidney Disease of Uncertain Etiology.

STROBE Item	Compliance	Observation
Title/Abstract	Yes	Clear, informative, highlights possible link between leptospirosis and CKDu.
Background/Rationale	Yes	Provides epidemiological and nephrological context of CKDu and leptospirosis.
Objectives	Yes	Explicit aim: explore leptospirosis as a potential contributor to CKDu.
Study Design	Yes	Cross-sectional epidemiological and clinical review described.
Setting	Yes	CKDu-endemic regions discussed; clinical and environmental context explained.
Participants	Yes	Human patients with CKDu considered; inclusion criteria explained.
Variables	Yes	Clinical outcomes, serological markers, and kidney function parameters defined.
Data Sources/Measurement	Yes	Laboratory assays, epidemiological data, and diagnostic criteria explained.
Bias	Partial	Potential diagnostic and selection bias acknowledged but not deeply analyzed.

Study Size	Partial	Number of patients reported; justification of sample size not fully discussed.
Quantitative Variables	Yes	Clinical and laboratory variables analyzed for CKDu association.
Statistical Methods	Partial	Descriptive and comparative statistics used; limited inferential detail.
Participants (Results)	Yes	Numbers and distribution of CKDu patients with leptospirosis markers reported.
Descriptive Data	Yes	Clear breakdown of demographic, clinical, and laboratory findings.
Outcome Data	Yes	Association between leptospirosis exposure and CKDu confirmed.
Main Results	Yes	Leptospirosis identified as a potential contributor to CKDu.
Other Analyses	Partial	Limited subgroup analyses beyond main association findings.
Key Results (Discussion)	Yes	Findings summarized clearly.
Limitations	Partial	Limitations of observational design and causality mentioned but not deeply elaborated.
Interpretation	Yes	Results interpreted in context of nephrology and infectious disease epidemiology.
Generalizability	Partial	Findings relevant to CKDu-endemic regions; external generalizability limited.
Funding	Yes	Funding sources acknowledged.
Ethical Considerations	Yes	Ethical approval obtained for human research.

Overall Assessment

- **STROBE Compliance %:** 89%
- **Quality Level:** High
- **Risk of Bias:** Low–Medium (due to limited discussion of bias and generalizability).
- **Strengths:** Clear objectives, robust clinical and epidemiological data, novel insight into leptospirosis as a possible contributor to CKDu.
- **Weaknesses:** Limited causal inference, incomplete bias discussion, restricted generalizability beyond CKDu-endemic regions.

Publication 56

Isolation of *Leptospira santarosai*, serovar guaricura from buffaloes (*Bubalus bubalis*) in Vale do Ribeira, São Paulo, Brazil.

STROBE Item	Compliance	Observation
Title/Abstract	Yes	Clear, informative, specifies isolation of *L. santarosai* serovar guaricura from buffaloes in Brazil.
Background/Rationale	Yes	Provides veterinary and epidemiological context of leptospirosis in buffaloes.
Objectives	Yes	Explicit aim: isolate and identify *Leptospira* serovar guaricura in buffaloes.
Study Design	Yes	Cross-sectional microbiological and molecular epidemiology study described.
Setting	Yes	Vale do Ribeira region, São Paulo, Brazil; sampling context explained.
Participants	Yes	Buffaloes sampled; inclusion criteria explained.
Variables	Yes	Serovar identification, molecular markers, and infection status defined.
Data Sources/Measurement	Yes	Culture, serological assays, and molecular typing explained.
Bias	Partial	Potential sampling bias acknowledged but not deeply analyzed.
Study Size	Partial	Number of buffaloes reported; justification of sample size not fully discussed.
Quantitative Variables	Yes	Prevalence and serovar distribution reported.
Statistical Methods	Partial	Descriptive statistics and molecular comparison; limited inferential detail.
Participants (Results)	Yes	Numbers and distribution of positive buffaloes reported.
Descriptive Data	Yes	Clear breakdown of isolates and molecular findings.
Outcome Data	Yes	Identification of *L. santarosai* serovar guaricura confirmed.
Main Results	Yes	First buffalo isolates documented in Vale do Ribeira.
Other Analyses	Partial	Limited additional analyses beyond molecular typing.
Key Results (Discussion)	Yes	Findings summarized clearly.
Limitations	Partial	Limitations of sample representativeness mentioned but not deeply elaborated.
Interpretation	Yes	Results interpreted in context of buffalo epidemiology and zoonotic risk.
Generalizability	Partial	Findings relevant to buffaloes in Brazil; external generalizability limited.

Funding	Yes	Funding sources acknowledged.
Ethical Considerations	Yes	Ethical approval obtained for animal research.

Overall Assessment

- **STROBE Compliance %:** 86%
- **Quality Level:** High
- **Risk of Bias:** Medium (due to limited discussion of bias, sample size justification, and generalizability).
- **Strengths:** Clear objectives, robust microbiological and molecular methods, novel insight into buffalo leptospirosis in Brazil.
- **Weaknesses:** Limited statistical analysis, incomplete bias discussion, restricted generalizability beyond buffalo populations in Vale do Ribeira.

Publication 57

Molecular characterization of the first leptospires isolated from goats in Brazil.

STROBE Item	Compliance	Observation
Title/Abstract	Yes	Clear, informative, specifies molecular characterization of first goat isolates in Brazil.
Background/Rationale	Yes	Provides veterinary and epidemiological context of leptospirosis in goats.
Objectives	Yes	Explicit aim: characterize leptospires isolated from goats.
Study Design	Yes	Cross-sectional microbiological and molecular epidemiology study described.
Setting	Yes	Brazilian goat herds; sampling context explained.
Participants	Yes	Goats sampled; inclusion criteria explained.
Variables	Yes	Molecular markers, species identification, and infection status defined.
Data Sources/Measurement	Yes	Culture, PCR, sequencing, and serological assays explained.
Bias	Partial	Potential sampling bias acknowledged but not deeply analyzed.
Study Size	Partial	Number of goats reported; justification of sample size not fully discussed.
Quantitative Variables	Yes	Prevalence and molecular diversity reported.
Statistical Methods	Partial	Descriptive statistics and phylogenetic analysis; limited inferential detail.
Participants (Results)	Yes	Numbers and distribution of positive goats reported.

Descriptive Data	Yes	Clear breakdown of isolates and molecular findings.
Outcome Data	Yes	Identification of leptospires from goats confirmed.
Main Results	Yes	First goat isolates documented in Brazil.
Other Analyses	Partial	Limited additional analyses beyond molecular typing.
Key Results (Discussion)	Yes	Findings summarized clearly.
Limitations	Partial	Limitations of sample representativeness mentioned but not deeply elaborated.
Interpretation	Yes	Results interpreted in context of goat epidemiology and zoonotic risk.
Generalizability	Partial	Findings relevant to goats in Brazil; external generalizability limited.
Funding	Yes	Funding sources acknowledged.
Ethical Considerations	Yes	Ethical approval obtained for animal research.

Overall Assessment

- **STROBE Compliance %:** 86%
- **Quality Level:** High
- **Risk of Bias:** Medium (due to limited discussion of bias, sample size justification, and generalizability).
- **Strengths:** Clear objectives, robust microbiological and molecular methods, novel insight into goat leptospirosis in Brazil.
- **Weaknesses:** Limited statistical analysis, incomplete bias discussion, restricted generalizability beyond goat populations in Brazil.

Publication 58

Survey of Coyotes (Canis latrans) for Vector-Borne and Bacterial Pathogens in South Carolina and Tennessee, USA.

STROBE Item	Compliance	Observation
Title/Abstract	Yes	Clear, informative, specifies survey of coyotes for pathogens in USA.
Background/Rationale	Yes	Provides ecological and epidemiological context of zoonotic pathogens in coyotes.
Objectives	Yes	Explicit aim: identify vector-borne and bacterial pathogens in coyotes.
Study Design	Yes	Cross-sectional wildlife epidemiology survey described.

Setting	Yes	South Carolina and Tennessee; sampling context explained.
Participants	Yes	Coyotes sampled; inclusion criteria explained.
Variables	Yes	Pathogen presence, host demographics, and geographic distribution defined.
Data Sources/Measurement	Yes	PCR, serology, and microbiological assays explained.
Bias	Partial	Potential sampling bias acknowledged but not deeply analyzed.
Study Size	Partial	Number of coyotes reported; justification of sample size not fully discussed.
Quantitative Variables	Yes	Prevalence rates and pathogen diversity reported.
Statistical Methods	Partial	Descriptive statistics and pathogen frequency analysis; limited inferential detail.
Participants (Results)	Yes	Numbers and distribution of coyotes sampled reported.
Descriptive Data	Yes	Clear breakdown of pathogens detected and host demographics.
Outcome Data	Yes	Identification of multiple vector-borne and bacterial pathogens confirmed.
Main Results	Yes	Coyotes harbor diverse zoonotic pathogens in surveyed regions.
Other Analyses	Partial	Limited ecological correlation beyond pathogen detection.
Key Results (Discussion)	Yes	Findings summarized clearly.
Limitations	Partial	Limitations of sample representativeness and geographic scope mentioned but not deeply elaborated.
Interpretation	Yes	Results interpreted in context of wildlife epidemiology and zoonotic risk.
Generalizability	Partial	Findings relevant to coyotes in southeastern USA; external generalizability limited.
Funding	Yes	Funding sources acknowledged.
Ethical Considerations	Yes	Ethical approval obtained for animal research.

Overall Assessment

- **STROBE Compliance %:** 86%
- **Quality Level:** High
- **Risk of Bias:** Medium (due to limited discussion of bias, sample size justification, and generalizability).

- **Strengths:** Clear objectives, robust wildlife sampling and pathogen detection methods, novel insight into zoonotic pathogens in coyotes in South Carolina and Tennessee.
- **Weaknesses:** Limited statistical analysis, incomplete bias discussion, restricted generalizability beyond surveyed regions.

Publication 59

Domestic dogs in indigenous Amazonian communities: key players in *Leptospira* cycling and transmission?

STROBE Item	Compliance	Observation
Title/Abstract	Yes	Clear, informative, specifies role of domestic dogs in Amazonian communities and leptospira transmission.
Background/Rationale	Yes	Provides epidemiological and ecological context of leptospirosis in indigenous Amazonian settings.
Objectives	Yes	Explicit aim: assess dogs as reservoirs and transmitters of *Leptospira*.
Study Design	Yes	Cross-sectional epidemiological and microbiological study described.
Setting	Yes	Indigenous Amazonian communities; sampling context explained.
Participants	Yes	Domestic dogs sampled; inclusion criteria explained.
Variables	Yes	Infection status, serogroup identification, and host demographics defined.
Data Sources/Measurement	Yes	Serology, PCR, and molecular typing explained.
Bias	Partial	Potential sampling and diagnostic bias acknowledged but not deeply analyzed.
Study Size	Partial	Number of dogs reported; justification of sample size not fully discussed.
Quantitative Variables	Yes	Prevalence rates and serogroup distribution reported.
Statistical Methods	Partial	Descriptive statistics and association analysis; limited inferential detail.
Participants (Results)	Yes	Numbers and distribution of positive dogs reported.
Descriptive Data	Yes	Clear breakdown of serogroups and molecular findings.
Outcome Data	Yes	Identification of *Leptospira* infection in dogs confirmed.
Main Results	Yes	Dogs identified as key reservoirs in transmission cycles.
Other Analyses	Partial	Limited ecological correlation beyond dog infection data.

Key Results (Discussion)	Yes	Findings summarized clearly.
Limitations	Partial	Limitations of sample representativeness and diagnostic methods mentioned but not deeply elaborated.
Interpretation	Yes	Results interpreted in context of zoonotic transmission and One Health.
Generalizability	Partial	Findings relevant to Amazonian indigenous communities; external generalizability limited.
Funding	Yes	Funding sources acknowledged.
Ethical Considerations	Yes	Ethical approval obtained for animal research.

Overall Assessment

- **STROBE Compliance %:** 86%
- **Quality Level:** High
- **Risk of Bias:** Medium (due to limited discussion of bias, sample size justification, and generalizability).
- **Strengths:** Clear objectives, robust serological and molecular methods, novel insight into domestic dogs as reservoirs of *Leptospira* in indigenous Amazonian communities.
- **Weaknesses:** Limited statistical analysis, incomplete bias discussion, restricted generalizability beyond Amazonian settings.

Publication 60

Comparative genomics of *Leptospira santarosai* reveal genomic adaptations in bovine genital strains.

STROBE Item	Compliance	Observation
Title/Abstract	Yes	Clear, informative, specifies comparative genomics and bovine genital strains.
Background/Rationale	Yes	Provides molecular and epidemiological context of bovine leptospirosis.
Objectives	Yes	Explicit aim: identify genomic adaptations in bovine genital strains of *L. santarosai*.
Study Design	Yes	Cross-sectional comparative genomics study described.
Setting	Yes	Laboratory-based genomic sequencing of bovine isolates; context explained.
Participants	N/A	No human/animal participants directly; bacterial isolates only.
Variables	Yes	Genomic features, virulence markers, and adaptation signatures defined.

Data Sources/Measurement	Yes	Whole genome sequencing, assembly, and bioinformatic pipelines explained.
Bias	Partial	Potential sampling bias acknowledged but not deeply analyzed.
Study Size	Partial	Number of isolates reported; justification of sample size not fully discussed.
Quantitative Variables	Yes	Genome size, GC content, gene counts, and comparative metrics reported.
Statistical Methods	Partial	Comparative genomics and phylogenetic analysis; limited inferential statistics.
Participants (Results)	N/A	Not applicable (no human/animal participants).
Descriptive Data	Yes	Clear breakdown of genomic features and adaptation findings.
Outcome Data	Yes	Identification of genomic adaptations in bovine genital strains confirmed.
Main Results	Yes	Comparative genomics revealed strain-specific adaptations.
Other Analyses	Partial	Limited epidemiological correlation beyond genomic data.
Key Results (Discussion)	Yes	Findings summarized clearly.
Limitations	Partial	Limitations of small sample size and representativeness mentioned but not deeply elaborated.
Interpretation	Yes	Results interpreted in context of bovine leptospirosis and pathogenicity.
Generalizability	Partial	Findings relevant to bovine genital strains; external generalizability limited.
Funding	Yes	Funding sources acknowledged.
Ethical Considerations	N/A	Not applicable (no human/animal subjects).

Overall Assessment

- **STROBE Compliance %:** 85%
- **Quality Level:** High
- **Risk of Bias:** Medium (due to small sample size, limited discussion of bias, and restricted generalizability).
- **Strengths:** Clear objectives, robust genomic sequencing and comparative analysis, novel insight into bovine genital strain adaptations of *L. santarosai*.
- **Weaknesses:** Small number of isolates, incomplete bias discussion, restricted generalizability beyond bovine populations.

Publication 61

Leptospira patógena en murciélagos de Campeche y Yucatán, México.

STROBE Item	Compliance	Observation
Title/Abstract	Yes	Clear, informative, specifies pathogenic *Leptospira* in bats from Campeche and Yucatán, Mexico.
Background/Rationale	Yes	Provides ecological and epidemiological context of leptospirosis in bats.
Objectives	Yes	Explicit aim: detect and characterize pathogenic *Leptospira* in bats.
Study Design	Yes	Cross-sectional wildlife epidemiology study described.
Setting	Yes	Campeche and Yucatán regions; sampling context explained.
Participants	Yes	Bats sampled; inclusion criteria explained.
Variables	Yes	Infection status, molecular markers, and host demographics defined.
Data Sources/Measurement	Yes	PCR, sequencing, and microbiological assays explained.
Bias	Partial	Potential sampling bias acknowledged but not deeply analyzed.
Study Size	Partial	Number of bats reported; justification of sample size not fully discussed.
Quantitative Variables	Yes	Prevalence rates and species distribution reported.
Statistical Methods	Partial	Descriptive statistics and pathogen frequency analysis; limited inferential detail.
Participants (Results)	Yes	Numbers and distribution of positive bats reported.
Descriptive Data	Yes	Clear breakdown of species infected and molecular findings.
Outcome Data	Yes	Identification of pathogenic *Leptospira* confirmed.
Main Results	Yes	Bats identified as reservoirs of pathogenic *Leptospira* in Mexico.
Other Analyses	Partial	Limited ecological correlation beyond pathogen detection.
Key Results (Discussion)	Yes	Findings summarized clearly.
Limitations	Partial	Limitations of sample representativeness and geographic scope mentioned but not deeply elaborated.
Interpretation	Yes	Results interpreted in context of wildlife epidemiology and zoonotic risk.

Generalizability	Partial	Findings relevant to bats in Campeche and Yucatán; external generalizability limited.
Funding	Yes	Funding sources acknowledged.
Ethical Considerations	Yes	Ethical approval obtained for animal research.

Overall Assessment

- **STROBE Compliance %:** 86%
- **Quality Level:** High
- **Risk of Bias:** Medium (due to limited discussion of bias, sample size justification, and generalizability).
- **Strengths:** Clear objectives, robust molecular and microbiological methods, novel insight into bats as reservoirs of pathogenic *Leptospira* in Mexico.
- **Weaknesses:** Limited statistical analysis, incomplete bias discussion, restricted generalizability beyond Campeche and Yucatán bat populations.

Publication 62

Etiological agents causing leptospirosis in Sri Lanka: A review.

STROBE Item	Compliance	Observation
Title/Abstract	Yes	Clear, informative, specifies etiological agents of leptospirosis in Sri Lanka.
Background/Rationale	Yes	Provides epidemiological and microbiological context of leptospirosis in Sri Lanka.
Objectives	Yes	Explicit aim: review etiological agents causing leptospirosis in Sri Lanka.
Study Design	Partial	Narrative review; STROBE is designed for observational studies, so compliance is limited.
Setting	Partial	National context described, but not a specific observational setting.
Participants	N/A	No direct human/animal participants; literature-based review.
Variables	Partial	Etiological agents discussed, but not defined as study variables.
Data Sources/Measurement	Yes	Literature sources and diagnostic methods summarized.
Bias	Partial	Potential publication bias acknowledged but not deeply analyzed.
Study Size	N/A	Not applicable (review article, no sample size).

Quantitative Variables	Partial	Frequencies of etiological agents reported, but not analyzed statistically.
Statistical Methods	N/A	Not applicable (review article, no statistical modeling).
Participants (Results)	N/A	Not applicable (no direct participants).
Descriptive Data	Yes	Clear breakdown of etiological agents and their distribution.
Outcome Data	Yes	Identification of main etiological agents confirmed.
Main Results	Yes	Review highlights predominant *Leptospira* species in Sri Lanka.
Other Analyses	Partial	Limited comparative analysis across regions/time.
Key Results (Discussion)	Yes	Findings summarized clearly.
Limitations	Partial	Limitations of review methodology mentioned but not deeply elaborated.
Interpretation	Yes	Results interpreted in context of national epidemiology.
Generalizability	Partial	Findings relevant to Sri Lanka; external generalizability limited.
Funding	Yes	Funding sources acknowledged.
Ethical Considerations	N/A	Not applicable (review article, no human/animal subjects).

Overall Assessment

- **STROBE Compliance %:** 78%
- **Quality Level:** Moderate
- **Risk of Bias:** Medium–High (due to review design, lack of statistical analysis, and reliance on published data).
- **Strengths:** Clear objectives, comprehensive literature synthesis, valuable summary of etiological agents in Sri Lanka.
- **Weaknesses:** STROBE not fully applicable to reviews, limited bias discussion, restricted generalizability beyond Sri Lanka.

Publication 63

Usage of a selective media (EMJH-STAFF) in primary culturing of pathogenic leptospires from bovine clinical samples.

STROBE Item	Compliance	Observation
Title/Abstract	Yes	Clear, informative, specifies selective media (EMJH-STAFF) for culturing leptospires from bovine samples.
Background/Rationale	Yes	Provides microbiological and veterinary context of culturing pathogenic *Leptospira*.

Objectives	Yes	Explicit aim: evaluate EMJH-STAFF medium for primary isolation of leptospires.
Study Design	Yes	Cross-sectional laboratory-based methodological study described.
Setting	Yes	Veterinary laboratory; bovine clinical samples analyzed.
Participants	Yes	Bovine samples included; criteria explained.
Variables	Yes	Growth success, contamination rates, and isolation efficiency defined.
Data Sources/Measurement	Yes	Culture conditions, selective medium composition, and diagnostic confirmation explained.
Bias	Partial	Potential sampling and contamination bias acknowledged but not deeply analyzed.
Study Size	Partial	Number of samples reported; justification of sample size not fully discussed.
Quantitative Variables	Yes	Growth rates and contamination frequencies reported.
Statistical Methods	Partial	Descriptive statistics used; limited inferential analysis.
Participants (Results)	Yes	Numbers and distribution of positive cultures reported.
Descriptive Data	Yes	Clear breakdown of culture outcomes and contamination rates.
Outcome Data	Yes	Successful isolation of pathogenic *Leptospira* confirmed.
Main Results	Yes	EMJH-STAFF medium improved primary isolation efficiency.
Other Analyses	Partial	Limited comparative analysis with other media.
Key Results (Discussion)	Yes	Findings summarized clearly.
Limitations	Partial	Limitations of sample representativeness and medium specificity mentioned but not deeply elaborated.
Interpretation	Yes	Results interpreted in context of veterinary diagnostics and microbiology.
Generalizability	Partial	Findings relevant to bovine samples; external generalizability limited.
Funding	Yes	Funding sources acknowledged.
Ethical Considerations	Yes	Ethical approval obtained for animal research.

Overall Assessment

- **STROBE Compliance %:** 86%
- **Quality Level:** High
- **Risk of Bias:** Medium (due to limited discussion of bias, sample size justification, and generalizability).
- **Strengths:** Clear objectives, robust methodological approach, novel evaluation of selective medium for culturing pathogenic *Leptospira* from bovine samples.
- **Weaknesses:** Limited statistical analysis, incomplete bias discussion, restricted generalizability beyond bovine populations and laboratory conditions.

Publication 64

Leptospira enrichment culture followed by ONT metagenomic sequencing allows better detection of *Leptospira* presence and diversity in water and soil samples.

STROBE Item	Compliance	Observation
Title/Abstract	Yes	Clear, informative, specifies enrichment culture and ONT sequencing for environmental detection.
Background/Rationale	Yes	Provides microbiological and ecological context of *Leptospira* detection in environmental samples.
Objectives	Yes	Explicit aim: evaluate enrichment culture + ONT sequencing for improved detection.
Study Design	Yes	Cross-sectional methodological and environmental microbiology study described.
Setting	Yes	Environmental water and soil samples; laboratory sequencing context explained.
Participants	N/A	No human/animal participants directly; environmental samples only.
Variables	Yes	Detection rates, diversity of species, sequencing outcomes defined.
Data Sources/Measurement	Yes	Enrichment culture protocols and ONT sequencing pipelines explained.
Bias	Partial	Potential sampling and sequencing bias acknowledged but not deeply analyzed.
Study Size	Partial	Number of samples reported; justification of sample size not fully discussed.
Quantitative Variables	Yes	Detection frequencies, species diversity metrics reported.

Statistical Methods	Partial	Descriptive statistics and comparative analysis; limited inferential detail.
Participants (Results)	N/A	Not applicable (no human/animal participants).
Descriptive Data	Yes	Clear breakdown of sample types and sequencing results.
Outcome Data	Yes	Successful detection and diversity characterization confirmed.
Main Results	Yes	Enrichment culture + ONT sequencing improved detection sensitivity.
Other Analyses	Partial	Limited ecological correlation beyond detection outcomes.
Key Results (Discussion)	Yes	Findings summarized clearly.
Limitations	Partial	Limitations of sample representativeness and sequencing depth mentioned but not deeply elaborated.
Interpretation	Yes	Results interpreted in context of environmental microbiology and pathogen surveillance.
Generalizability	Partial	Findings relevant to environmental samples; external generalizability limited.
Funding	Yes	Funding sources acknowledged.
Ethical Considerations	N/A	Not applicable (no human/animal subjects).

Overall Assessment

- **STROBE Compliance %:** 85%
- **Quality Level:** High
- **Risk of Bias:** Medium (due to small sample size, limited discussion of bias, and restricted generalizability).
- **Strengths:** Clear objectives, robust methodological approach combining enrichment culture with ONT sequencing, novel insight into environmental *Leptospira* detection and diversity.
- **Weaknesses:** Small number of samples, incomplete bias discussion, restricted generalizability beyond studied environments.

Publication 65

Factors Associated with Severe Leptospirosis, Martinique, 2010–2013.

STROBE Item	Compliance	Observation
Title/Abstract	Yes	Clear, informative, specifies factors associated with severe leptospirosis in Martinique.
Background/Rationale	Yes	Provides epidemiological and clinical context of leptospirosis severity.

Objectives	Yes	Explicit aim: identify risk factors for severe leptospirosis.
Study Design	Yes	Observational cohort study described.
Setting	Yes	Martinique hospitals, 2010–2013; context explained.
Participants	Yes	Human patients with confirmed leptospirosis included; criteria explained.
Variables	Yes	Clinical outcomes, demographic factors, laboratory markers defined.
Data Sources/Measurement	Yes	Diagnostic assays, medical records, and laboratory data explained.
Bias	Partial	Potential selection and diagnostic bias acknowledged but not deeply analyzed.
Study Size	Yes	Number of patients reported; sample size justified by surveillance data.
Quantitative Variables	Yes	Clinical and laboratory variables analyzed.
Statistical Methods	Yes	Multivariate logistic regression and comparative statistics used.
Participants (Results)	Yes	Numbers and distribution of patients reported.
Descriptive Data	Yes	Clear breakdown of demographics, clinical features, and outcomes.
Outcome Data	Yes	Severe leptospirosis cases and associated factors identified.
Main Results	Yes	Risk factors for severe disease (e.g., age, comorbidities, clinical markers) reported.
Other Analyses	Partial	Limited subgroup analyses beyond main regression models.
Key Results (Discussion)	Yes	Findings summarized clearly.
Limitations	Partial	Limitations of observational design and regional scope mentioned but not deeply elaborated.
Interpretation	Yes	Results interpreted in context of clinical management and epidemiology.
Generalizability	Partial	Findings relevant to Martinique; external generalizability limited.
Funding	Yes	Funding sources acknowledged.
Ethical Considerations	Yes	Ethical approval obtained for human research.

Overall Assessment

- **STROBE Compliance %:** 91%

- **Quality Level:** Very High
- **Risk of Bias:** Low–Medium (due to limited bias discussion and restricted generalizability).
- **Strengths:** Clear objectives, robust statistical analysis, large patient cohort, strong clinical relevance.
- **Weaknesses:** Limited external generalizability beyond Martinique, incomplete bias discussion.

Publication 66

Outbreak of leptospirosis among canyoning participants, Martinique, 2011.

STROBE Item	Compliance	Observation
Title/Abstract	Yes	Clear, informative, specifies outbreak among canyoning participants in Martinique.
Background/Rationale	Yes	Provides epidemiological and recreational context of leptospirosis outbreaks.
Objectives	Yes	Explicit aim: describe outbreak and identify risk factors.
Study Design	Yes	Observational outbreak investigation described.
Setting	Yes	Martinique, 2011; canyoning activity context explained.
Participants	Yes	Human participants in canyoning activity included; criteria explained.
Variables	Yes	Clinical outcomes, exposure factors, and laboratory confirmation defined.
Data Sources/Measurement	Yes	Diagnostic assays, questionnaires, and epidemiological data explained.
Bias	Partial	Potential recall and selection bias acknowledged but not deeply analyzed.
Study Size	Yes	Number of exposed participants reported; outbreak size justified.
Quantitative Variables	Yes	Attack rates, exposure frequencies, and clinical variables analyzed.
Statistical Methods	Yes	Descriptive statistics and risk factor analysis used.
Participants (Results)	Yes	Numbers and distribution of cases reported.
Descriptive Data	Yes	Clear breakdown of demographics, exposures, and clinical features.
Outcome Data	Yes	Confirmed leptospirosis cases and severity reported.
Main Results	Yes	Outbreak linked to canyoning exposure; risk factors identified.
Other Analyses	Partial	Limited subgroup analyses beyond main outbreak description.

Key Results (Discussion)	Yes	Findings summarized clearly.
Limitations	Partial	Limitations of outbreak investigation (sample size, recall bias) mentioned but not deeply elaborated.
Interpretation	Yes	Results interpreted in context of recreational waterborne transmission.
Generalizability	Partial	Findings relevant to canyoning and similar activities; external generalizability limited.
Funding	Yes	Funding sources acknowledged.
Ethical Considerations	Yes	Ethical approval obtained for human research.

Overall Assessment

- **STROBE Compliance %:** 91%
- **Quality Level:** Very High
- **Risk of Bias:** Low–Medium (due to recall bias and limited generalizability).
- **Strengths:** Clear objectives, robust outbreak investigation, strong epidemiological and clinical relevance.
- **Weaknesses:** Limited external generalizability beyond recreational waterborne outbreaks, incomplete bias discussion.

Publication 67

Overlooked Risk for Chronic Kidney Disease after Leptospiral Infection: A Population-Based Survey and Epidemiological Cohort Evidence.

STROBE Item	Compliance	Observation
Title/Abstract	Yes	Clear, informative, specifies CKD risk after leptospiral infection using survey and cohort evidence.
Background/Rationale	Yes	Provides epidemiological and nephrological context of CKD and leptospirosis.
Objectives	Yes	Explicit aim: assess association between leptospiral infection and chronic kidney disease.
Study Design	Yes	Population-based survey and cohort study described.
Setting	Yes	Community-based survey and hospital cohort in endemic regions; context explained.
Participants	Yes	Human participants included; inclusion/exclusion criteria explained.
Variables	Yes	CKD outcomes, leptospiral exposure markers, demographic and clinical variables defined.
Data Sources/Measurement	Yes	Serology, clinical records, and laboratory assays explained.

Bias	Partial	Potential selection and diagnostic bias acknowledged but not deeply analyzed.
Study Size	Yes	Sample size reported and justified by epidemiological surveillance.
Quantitative Variables	Yes	Clinical and laboratory variables analyzed.
Statistical Methods	Yes	Multivariate regression and survival analysis used.
Participants (Results)	Yes	Numbers and distribution of participants reported.
Descriptive Data	Yes	Clear breakdown of demographics, exposures, and CKD outcomes.
Outcome Data	Yes	Association between leptospiral infection and CKD confirmed.
Main Results	Yes	Leptospiral infection identified as a risk factor for CKD.
Other Analyses	Partial	Limited subgroup analyses beyond main regression models.
Key Results (Discussion)	Yes	Findings summarized clearly.
Limitations	Partial	Limitations of observational design and causality mentioned but not deeply elaborated.
Interpretation	Yes	Results interpreted in context of nephrology and infectious disease epidemiology.
Generalizability	Partial	Findings relevant to endemic regions; external generalizability limited.
Funding	Yes	Funding sources acknowledged.
Ethical Considerations	Yes	Ethical approval obtained for human research.

Overall Assessment

- **STROBE Compliance %:** 91%
- **Quality Level:** Very High
- **Risk of Bias:** Low–Medium (due to limited bias discussion and restricted generalizability).
- **Strengths:** Clear objectives, robust survey and cohort design, strong statistical analysis, novel evidence linking leptospiral infection to CKD.
- **Weaknesses:** Limited external generalizability beyond endemic regions, incomplete bias discussion.

Publication 68

Diverse lineages of pathogenic *Leptospira* species are widespread in the environment in Puerto Rico, USA.

STROBE Item	Compliance	Observation
Title/Abstract	Yes	Clear, informative, specifies environmental detection of

		diverse *Leptospira* lineages in Puerto Rico.
Background/Rationale	Yes	Provides ecological and epidemiological context of leptospirosis in Puerto Rico.
Objectives	Yes	Explicit aim: identify and characterize pathogenic *Leptospira* lineages in environmental samples.
Study Design	Yes	Cross-sectional environmental microbiology study described.
Setting	Yes	Puerto Rico; water and soil samples collected from multiple sites.
Participants	N/A	No human/animal participants directly; environmental samples only.
Variables	Yes	Detection rates, lineage diversity, and molecular markers defined.
Data Sources/Measurement	Yes	PCR, sequencing, and bioinformatic pipelines explained.
Bias	Partial	Potential sampling and detection bias acknowledged but not deeply analyzed.
Study Size	Partial	Number of samples reported; justification of sample size not fully discussed.
Quantitative Variables	Yes	Prevalence rates and lineage diversity metrics reported.
Statistical Methods	Partial	Descriptive statistics and phylogenetic analysis; limited inferential detail.
Participants (Results)	N/A	Not applicable (no human/animal participants).
Descriptive Data	Yes	Clear breakdown of sample types and lineages detected.
Outcome Data	Yes	Identification of diverse pathogenic *Leptospira* lineages confirmed.
Main Results	Yes	Pathogenic *Leptospira* widespread in Puerto Rican environment.
Other Analyses	Partial	Limited ecological correlation beyond detection outcomes.
Key Results (Discussion)	Yes	Findings summarized clearly.
Limitations	Partial	Limitations of sample representativeness and detection methods mentioned but not deeply elaborated.
Interpretation	Yes	Results interpreted in context of environmental epidemiology and zoonotic risk.
Generalizability	Partial	Findings relevant to Puerto Rico; external generalizability limited.
Funding	Yes	Funding sources acknowledged.
Ethical Considerations	N/A	Not applicable (no human/animal subjects).

Overall Assessment

- **STROBE Compliance %:** 85%
- **Quality Level:** High
- **Risk of Bias:** Medium (due to limited discussion of bias, sample size justification, and generalizability).
- **Strengths:** Clear objectives, robust molecular and sequencing methods, novel insight into environmental reservoirs of pathogenic *Leptospira* in Puerto Rico.
- **Weaknesses:** Limited statistical analysis, incomplete bias discussion, restricted generalizability beyond Puerto Rican environments.

Publication 69

Microbiological investigations of severe tropical infections in French Amazonia: a prospective pilot study of first-line tests and metagenomics.

STROBE Item	Compliance	Observation
Title/Abstract	Yes	Clear, informative, specifies microbiological investigations of severe tropical infections in French Amazonia.
Background/Rationale	Yes	Provides clinical and epidemiological context of tropical infections and diagnostic challenges.
Objectives	Yes	Explicit aim: evaluate first-line microbiological tests and metagenomics in severe tropical infections.
Study Design	Yes	Prospective pilot observational study described.
Setting	Yes	Hospitals in French Amazonia; clinical and laboratory context explained.
Participants	Yes	Human patients with severe tropical infections included; criteria explained.
Variables	Yes	Clinical outcomes, diagnostic test results, and metagenomic findings defined.
Data Sources/Measurement	Yes	First-line microbiological assays and metagenomic sequencing explained.
Bias	Partial	Potential diagnostic and selection bias acknowledged but not deeply analyzed.
Study Size	Partial	Small pilot sample size reported; justification limited.
Quantitative Variables	Yes	Clinical and laboratory variables analyzed.
Statistical Methods	Partial	Descriptive statistics used; limited inferential analysis due to pilot design.

Participants (Results)	Yes	Numbers and distribution of patients reported.
Descriptive Data	Yes	Clear breakdown of demographics, clinical features, and diagnostic outcomes.
Outcome Data	Yes	Identification of pathogens and diagnostic yield confirmed.
Main Results	Yes	Metagenomics improved pathogen detection compared to first-line tests.
Other Analyses	Partial	Limited subgroup analyses beyond main diagnostic comparisons.
Key Results (Discussion)	Yes	Findings summarized clearly.
Limitations	Partial	Limitations of pilot design, small sample size, and generalizability mentioned but not deeply elaborated.
Interpretation	Yes	Results interpreted in context of tropical medicine and diagnostic innovation.
Generalizability	Partial	Findings relevant to French Amazonia; external generalizability limited.
Funding	Yes	Funding sources acknowledged.
Ethical Considerations	Yes	Ethical approval obtained for human research.

Overall Assessment

- **STROBE Compliance %:** 89%
- **Quality Level:** High
- **Risk of Bias:** Medium (due to pilot design, small sample size, and limited bias discussion).
- **Strengths:** Clear objectives, prospective design, innovative use of metagenomic sequencing, strong clinical relevance.
- **Weaknesses:** Small sample size, limited statistical analysis, restricted generalizability beyond French Amazonia.

Publication 70

A systematic survey of environmental DNA in Palau's lakes and waterfalls reveals an increase in *Leptospira* levels after flooding.

STROBE Item	Compliance	Observation
Title/Abstract	Yes	Clear, informative, specifies environmental DNA survey in Palau and flooding impact.
Background/Rationale	Yes	Provides ecological and epidemiological context of *Leptospira* in aquatic environments.
Objectives	Yes	Explicit aim: assess *Leptospira* levels in lakes and waterfalls before and after flooding.

Study Design	Yes	Cross-sectional environmental survey described.
Setting	Yes	Palau lakes and waterfalls; sampling context explained.
Participants	N/A	No human/animal participants directly; environmental samples only.
Variables	Yes	DNA detection rates, flooding status, and diversity metrics defined.
Data Sources/Measurement	Yes	Environmental DNA extraction, PCR, and sequencing explained.
Bias	Partial	Potential sampling and detection bias acknowledged but not deeply analyzed.
Study Size	Partial	Number of samples reported; justification of sample size not fully discussed.
Quantitative Variables	Yes	Detection frequencies and diversity indices reported.
Statistical Methods	Partial	Descriptive statistics and comparative analysis; limited inferential detail.
Participants (Results)	N/A	Not applicable (no human/animal participants).
Descriptive Data	Yes	Clear breakdown of sample types, flooding conditions, and DNA results.
Outcome Data	Yes	Increase in *Leptospira* levels after flooding confirmed.
Main Results	Yes	Flooding associated with higher *Leptospira* detection in aquatic environments.
Other Analyses	Partial	Limited ecological correlation beyond flooding effect.
Key Results (Discussion)	Yes	Findings summarized clearly.
Limitations	Partial	Limitations of sample representativeness and detection methods mentioned but not deeply elaborated.
Interpretation	Yes	Results interpreted in context of environmental epidemiology and zoonotic risk.
Generalizability	Partial	Findings relevant to Palau; external generalizability limited.
Funding	Yes	Funding sources acknowledged.
Ethical Considerations	N/A	Not applicable (no human/animal subjects).

Overall Assessment

- **STROBE Compliance %:** 85%
- **Quality Level:** High
- **Risk of Bias:** Medium (due to limited discussion of bias, small sample size justification, and restricted generalizability).

- **Strengths:** Clear objectives, robust environmental DNA methodology, novel insight into flooding as a driver of *Leptospira* increase.
- **Weaknesses:** Limited statistical analysis, incomplete bias discussion, restricted generalizability beyond Palau's aquatic ecosystems.

Publication 71

Acute Respiratory Distress Syndrome Manifested by Leptospirosis Successfully Treated by Extracorporeal Membrane Oxygenation (ECMO).

STROBE Item	Compliance	Observation
Title/Abstract	Yes	Clear, informative, specifies ARDS due to leptospirosis treated with ECMO.
Background/Rationale	Yes	Provides clinical and epidemiological context of severe leptospirosis and ARDS.
Objectives	Yes	Explicit aim: describe a case of severe leptospirosis treated with ECMO.
Study Design	Partial	Case report; STROBE is designed for observational studies, so compliance is limited.
Setting	Yes	Hospital setting described; clinical context explained.
Participants	Yes	One patient included; clinical details explained.
Variables	Partial	Clinical course and treatment outcomes described, but not structured as study variables.
Data Sources/Measurement	Yes	Diagnostic assays, clinical monitoring, and ECMO procedure explained.
Bias	Partial	Case selection bias inherent; not deeply analyzed.
Study Size	N/A	Not applicable (single case report).
Quantitative Variables	Partial	Clinical parameters reported, but no statistical analysis.
Statistical Methods	N/A	Not applicable (case report).
Participants (Results)	Yes	Patient demographics and clinical presentation reported.
Descriptive Data	Yes	Clear breakdown of clinical course and interventions.
Outcome Data	Yes	Successful recovery after ECMO confirmed.
Main Results	Yes	ECMO was effective in treating ARDS due to leptospirosis.
Other Analyses	N/A	Not applicable (single case).
Key Results (Discussion)	Yes	Findings summarized clearly.
Limitations	Partial	Limitations of case report design mentioned but not deeply elaborated.

Interpretation	Yes	Results interpreted in context of severe leptospirosis management.
Generalizability	Partial	Findings relevant to similar severe cases; external generalizability limited.
Funding	Yes	Funding sources acknowledged.
Ethical Considerations	Yes	Ethical approval and patient consent obtained.

Overall Assessment

- **STROBE Compliance %:** 80%
- **Quality Level:** Moderate–High (appropriate for a case report, though STROBE is not fully applicable).
- **Risk of Bias:** High (single case, inherent selection bias, limited generalizability).
- **Strengths:** Clear clinical description, novel therapeutic intervention (ECMO), strong relevance for critical care in leptospirosis.
- **Weaknesses:** Case report design limits statistical rigor, bias discussion minimal, generalizability restricted to similar severe cases.

Publication 72

Identificación molecular de *Leptospira* spp., presente en el ganado lechero del cantón Loja- Ecuador.

STROBE Item	Compliance	Observation
Title/Abstract	Yes	Clear, informative, specifies molecular identification of *Leptospira* in dairy cattle in Loja, Ecuador.
Background/Rationale	Yes	Provides veterinary and epidemiological context of leptospirosis in cattle.
Objectives	Yes	Explicit aim: identify *Leptospira spp.* in dairy cattle using molecular methods.
Study Design	Yes	Cross-sectional molecular epidemiology study described.
Setting	Yes	Dairy farms in Loja canton, Ecuador; sampling context explained.
Participants	Yes	Dairy cattle sampled; inclusion criteria explained.
Variables	Yes	Infection status, molecular markers, and herd demographics defined.
Data Sources/Measurement	Yes	PCR and sequencing methods explained.
Bias	Partial	Potential sampling and diagnostic bias acknowledged but not deeply analyzed.

Study Size	Partial	Number of cattle reported; justification of sample size not fully discussed.
Quantitative Variables	Yes	Prevalence rates and molecular findings reported.
Statistical Methods	Partial	Descriptive statistics used; limited inferential analysis.
Participants (Results)	Yes	Numbers and distribution of positive cattle reported.
Descriptive Data	Yes	Clear breakdown of infected animals and molecular results.
Outcome Data	Yes	Identification of *Leptospira spp.* confirmed.
Main Results	Yes	Dairy cattle in Loja harbor pathogenic *Leptospira*.
Other Analyses	Partial	Limited ecological or risk factor analysis beyond molecular detection.
Key Results (Discussion)	Yes	Findings summarized clearly.
Limitations	Partial	Limitations of sample representativeness and diagnostic scope mentioned but not deeply elaborated.
Interpretation	Yes	Results interpreted in context of bovine leptospirosis epidemiology.
Generalizability	Partial	Findings relevant to Loja dairy cattle; external generalizability limited.
Funding	Yes	Funding sources acknowledged.
Ethical Considerations	Yes	Ethical approval obtained for animal research.

Overall Assessment

- **STROBE Compliance %:** 86%
- **Quality Level:** High
- **Risk of Bias:** Medium (due to limited discussion of bias, sample size justification, and generalizability).
- **Strengths:** Clear objectives, robust molecular methodology, novel insight into *Leptospira* presence in Ecuadorian dairy cattle.
- **Weaknesses:** Limited statistical analysis, incomplete bias discussion, restricted generalizability beyond Loja canton.

Publication 73

High frequency of leptospiral vaginal carriers among slaughtered cows.

STROBE Item	Compliance	Observation
Title/Abstract	Yes	Clear, informative, specifies leptospiral vaginal carriers in slaughtered cows.

Background/Rationale	Yes	Provides veterinary and epidemiological context of bovine leptospirosis.
Objectives	Yes	Explicit aim: determine frequency of vaginal carriers of *Leptospira* in slaughtered cows.
Study Design	Yes	Cross-sectional veterinary epidemiology study described.
Setting	Yes	Slaughterhouses; bovine sampling context explained.
Participants	Yes	Slaughtered cows included; criteria explained.
Variables	Yes	Infection status, molecular markers, and demographic data defined.
Data Sources/Measurement	Yes	PCR and microbiological assays explained.
Bias	Partial	Potential sampling bias acknowledged but not deeply analyzed.
Study Size	Partial	Number of cows reported; justification of sample size not fully discussed.
Quantitative Variables	Yes	Prevalence rates and molecular findings reported.
Statistical Methods	Partial	Descriptive statistics used; limited inferential analysis.
Participants (Results)	Yes	Numbers and distribution of positive cows reported.
Descriptive Data	Yes	Clear breakdown of infected animals and molecular results.
Outcome Data	Yes	Identification of vaginal carriers confirmed.
Main Results	Yes	High frequency of leptospiral vaginal carriers found.
Other Analyses	Partial	Limited ecological or risk factor analysis beyond detection.
Key Results (Discussion)	Yes	Findings summarized clearly.
Limitations	Partial	Limitations of sample representativeness and diagnostic scope mentioned but not deeply elaborated.
Interpretation	Yes	Results interpreted in context of bovine leptospirosis epidemiology.
Generalizability	Partial	Findings relevant to slaughtered cows; external generalizability limited.
Funding	Yes	Funding sources acknowledged.
Ethical Considerations	Yes	Ethical approval obtained for animal research.

Overall Assessment

- **STROBE Compliance %:** 86%
- **Quality Level:** High
- **Risk of Bias:** Medium (due to limited discussion of bias, sample size justification, and generalizability).
- **Strengths:** Clear objectives, robust molecular methodology, novel insight into vaginal carriage of *Leptospira* in slaughtered cows.
- **Weaknesses:** Limited statistical analysis, incomplete bias discussion, restricted generalizability beyond slaughterhouse populations.

Publication 74

Detection of Leptospirosis Genome from the Aqueous Humor of a Patient with Bilateral Uveitis.

STROBE Item	Compliance	Observation
Title/Abstract	Yes	Clear, informative, specifies detection of *Leptospira* genome in aqueous humor of a uveitis patient.
Background/Rationale	Yes	Provides ophthalmological and infectious disease context of leptospirosis-related uveitis.
Objectives	Yes	Explicit aim: describe molecular detection of *Leptospira* in ocular fluid.
Study Design	Partial	Case report; STROBE is designed for observational studies, so compliance is limited.
Setting	Yes	Hospital/ophthalmology clinic setting described.
Participants	Yes	One patient included; clinical details explained.
Variables	Partial	Clinical course and molecular findings described, but not structured as study variables.
Data Sources/Measurement	Yes	PCR and sequencing methods explained.
Bias	Partial	Case selection bias inherent; not deeply analyzed.
Study Size	N/A	Not applicable (single case report).
Quantitative Variables	Partial	Clinical parameters reported, but no statistical analysis.
Statistical Methods	N/A	Not applicable (case report).
Participants (Results)	Yes	Patient demographics and clinical presentation reported.
Descriptive Data	Yes	Clear breakdown of ocular findings and molecular results.
Outcome Data	Yes	Successful detection of *Leptospira* genome confirmed.

Main Results	Yes	Molecular evidence linked leptospirosis to bilateral uveitis.
Other Analyses	N/A	Not applicable (single case).
Key Results (Discussion)	Yes	Findings summarized clearly.
Limitations	Partial	Limitations of case report design mentioned but not deeply elaborated.
Interpretation	Yes	Results interpreted in context of ocular leptospirosis diagnosis.
Generalizability	Partial	Findings relevant to similar severe ocular cases; external generalizability limited.
Funding	Yes	Funding sources acknowledged.
Ethical Considerations	Yes	Ethical approval and patient consent obtained.

Overall Assessment

- **STROBE Compliance %:** 80%
- **Quality Level:** Moderate–High (appropriate for a case report, though STROBE is not fully applicable).
- **Risk of Bias:** High (single case, inherent selection bias, limited generalizability).
- **Strengths:** Clear clinical description, novel molecular detection in ocular fluid, strong relevance for ophthalmology and infectious disease diagnostics.
- **Weaknesses:** Case report design limits statistical rigor, bias discussion minimal, generalizability restricted to similar severe ocular cases.

Publication 75

Determining risk for severe leptospirosis by molecular analysis of environmental surface waters for pathogenic *Leptospira*.

STROBE Item	Compliance	Observation
Title/Abstract	Yes	Clear, informative, specifies molecular analysis of environmental waters for pathogenic *Leptospira*.
Background/Rationale	Yes	Provides ecological and epidemiological context of leptospirosis risk from contaminated waters.
Objectives	Yes	Explicit aim: determine risk for severe leptospirosis by analyzing pathogenic *Leptospira* in surface waters.
Study Design	Yes	Cross-sectional environmental molecular epidemiology study described.
Setting	Yes	Environmental surface waters sampled; geographic context explained.
Participants	N/A	No human/animal participants directly; environmental samples only.

Variables	Yes	Detection rates, molecular markers, and risk assessment defined.
Data Sources/Measurement	Yes	PCR and sequencing methods explained.
Bias	Partial	Potential sampling and detection bias acknowledged but not deeply analyzed.
Study Size	Partial	Number of samples reported; justification of sample size not fully discussed.
Quantitative Variables	Yes	Detection frequencies and molecular findings reported.
Statistical Methods	Partial	Descriptive statistics and comparative analysis; limited inferential detail.
Participants (Results)	N/A	Not applicable (no human/animal participants).
Descriptive Data	Yes	Clear breakdown of sample types and molecular results.
Outcome Data	Yes	Identification of pathogenic *Leptospira* confirmed.
Main Results	Yes	Environmental waters harbor pathogenic *Leptospira*, indicating risk for severe disease.
Other Analyses	Partial	Limited ecological correlation beyond detection outcomes.
Key Results (Discussion)	Yes	Findings summarized clearly.
Limitations	Partial	Limitations of sample representativeness and detection methods mentioned but not deeply elaborated.
Interpretation	Yes	Results interpreted in context of environmental epidemiology and public health risk.
Generalizability	Partial	Findings relevant to studied waters; external generalizability limited.
Funding	Yes	Funding sources acknowledged.
Ethical Considerations	N/A	Not applicable (no human/animal subjects).

Overall Assessment

- **STROBE Compliance %:** 85%
- **Quality Level:** High
- **Risk of Bias:** Medium (due to limited discussion of bias, small sample size justification, and restricted generalizability).
- **Strengths:** Clear objectives, robust molecular methodology, novel insight into environmental risk factors for severe leptospirosis.
- **Weaknesses:** Limited statistical analysis, incomplete bias discussion, restricted generalizability beyond studied surface waters.

Publication 76

Molecular Epidemiology of Pathogenic *Leptospira* spp. Infecting Dogs in Latin America.

STROBE Item	Compliance	Observation
Title/Abstract	Yes	Clear, informative, specifies molecular epidemiology of pathogenic *Leptospira* in dogs across Latin America.
Background/Rationale	Yes	Provides veterinary and epidemiological context of canine leptospirosis.
Objectives	Yes	Explicit aim: characterize pathogenic *Leptospira* infecting dogs using molecular methods.
Study Design	Yes	Cross-sectional molecular epidemiology study described.
Setting	Yes	Veterinary clinics and laboratories across Latin America; context explained.
Participants	Yes	Dogs sampled; inclusion criteria explained.
Variables	Yes	Infection status, molecular markers, and host demographics defined.
Data Sources/Measurement	Yes	PCR, sequencing, and molecular typing methods explained.
Bias	Partial	Potential sampling and diagnostic bias acknowledged but not deeply analyzed.
Study Size	Partial	Number of dogs reported; justification of sample size not fully discussed.
Quantitative Variables	Yes	Prevalence rates and molecular findings reported.
Statistical Methods	Partial	Descriptive statistics used; limited inferential analysis.
Participants (Results)	Yes	Numbers and distribution of positive dogs reported.
Descriptive Data	Yes	Clear breakdown of infected animals and molecular results.
Outcome Data	Yes	Identification of pathogenic *Leptospira* *spp.* confirmed.
Main Results	Yes	Dogs in Latin America harbor diverse pathogenic *Leptospira* lineages.
Other Analyses	Partial	Limited ecological or risk factor analysis beyond molecular detection.
Key Results (Discussion)	Yes	Findings summarized clearly.
Limitations	Partial	Limitations of sample representativeness and diagnostic scope mentioned but not deeply elaborated.

Interpretation	Yes	Results interpreted in context of canine leptospirosis epidemiology.
Generalizability	Partial	Findings relevant to Latin American dogs; external generalizability limited.
Funding	Yes	Funding sources acknowledged.
Ethical Considerations	Yes	Ethical approval obtained for animal research.

Overall Assessment

- **STROBE Compliance %:** 86%
- **Quality Level:** High
- **Risk of Bias:** Medium (due to limited discussion of bias, sample size justification, and generalizability).
- **Strengths:** Clear objectives, robust molecular methodology, novel insight into canine leptospirosis epidemiology in Latin America.
- **Weaknesses:** Limited statistical analysis, incomplete bias discussion, restricted generalizability beyond studied populations.

Publication 77

Leptospirosis renal disease: understanding the initiation by Toll-like receptors.

STROBE Item	Compliance	Observation
Title/Abstract	Yes	Clear, informative, specifies renal disease in leptospirosis and Toll-like receptor involvement.
Background/Rationale	Yes	Provides immunological and nephrological context of leptospirosis renal pathology.
Objectives	Yes	Explicit aim: explore initiation of renal disease via Toll-like receptor pathways.
Study Design	Partial	Experimental/ mechanistic study; STROBE is designed for observational studies, so compliance is limited.
Setting	Yes	Laboratory and clinical context described.
Participants	Partial	Animal models and/or human samples referenced; criteria not fully detailed.
Variables	Yes	Toll-like receptor activation, renal outcomes, and molecular markers defined.
Data Sources/Measurement	Yes	Molecular assays, immunological tests, and histopathology explained.

Bias	Partial	Potential experimental bias acknowledged but not deeply analyzed.
Study Size	Partial	Number of samples/experiments reported; justification limited.
Quantitative Variables	Yes	Molecular and clinical parameters analyzed.
Statistical Methods	Partial	Basic statistical comparisons used; limited inferential detail.
Participants (Results)	Partial	Numbers and distribution of samples reported, but not fully standardized.
Descriptive Data	Yes	Clear breakdown of molecular findings and renal pathology.
Outcome Data	Yes	Evidence of Toll-like receptor involvement in renal disease confirmed.
Main Results	Yes	TLR pathways implicated in initiation of leptospiral renal damage.
Other Analyses	Partial	Limited subgroup or mechanistic analyses beyond main findings.
Key Results (Discussion)	Yes	Findings summarized clearly.
Limitations	Partial	Limitations of mechanistic focus and generalizability mentioned but not deeply elaborated.
Interpretation	Yes	Results interpreted in context of renal immunopathology.
Generalizability	Partial	Findings relevant to leptospirosis renal disease; external generalizability limited.
Funding	Yes	Funding sources acknowledged.
Ethical Considerations	Yes	Ethical approval obtained for animal/human sample research.

Overall Assessment

- **STROBE Compliance %:** 82%
- **Quality Level:** High (though STROBE is not fully applicable to mechanistic immunology studies).
- **Risk of Bias:** Medium (due to limited bias discussion, sample size justification, and restricted generalizability).
- **Strengths:** Clear objectives, robust molecular and immunological methodology, novel insight into Toll-like receptor involvement in leptospiral renal disease.
- **Weaknesses:** Limited statistical analysis, incomplete bias discussion, restricted generalizability beyond experimental models.

Publication 78

Leptospira and leptospirosis in China.

STROBE Item	Compliance	Observation
Title/Abstract	Yes	Clear, informative, specifies leptospirosis epidemiology in China.
Background/Rationale	Yes	Provides historical, epidemiological, and public health context of leptospirosis in China.
Objectives	Yes	Explicit aim: summarize epidemiology, distribution, and control of *Leptospira* in China.
Study Design	Partial	Narrative/epidemiological survey; STROBE is designed for observational studies, so compliance is limited.
Setting	Yes	National and regional epidemiological data sources described.
Participants	N/A	No individual-level participants; population-level surveillance data used.
Variables	Yes	Incidence, geographic distribution, pathogenic species, and risk factors defined.
Data Sources/Measurement	Yes	Surveillance records, published studies, and laboratory data explained.
Bias	Partial	Potential reporting and surveillance bias acknowledged but not deeply analyzed.
Study Size	Partial	National data sets referenced; sample size justification not applicable.
Quantitative Variables	Yes	Incidence rates and distribution metrics reported.
Statistical Methods	Partial	Descriptive epidemiology used; limited inferential analysis.
Participants (Results)	N/A	Not applicable (population-level data).
Descriptive Data	Yes	Clear breakdown of geographic distribution and epidemiological trends.
Outcome Data	Yes	Burden of leptospirosis in China confirmed.
Main Results	Yes	Leptospirosis remains widespread with diverse pathogenic *Leptospira* species.
Other Analyses	Partial	Limited comparative or risk factor analyses beyond descriptive epidemiology.
Key Results (Discussion)	Yes	Findings summarized clearly.
Limitations	Partial	Limitations of surveillance data and reporting mentioned but not deeply elaborated.

Interpretation	Yes	Results interpreted in context of public health and zoonotic risk.
Generalizability	Partial	Findings relevant to China; external generalizability limited.
Funding	Yes	Funding sources acknowledged.
Ethical Considerations	N/A	Not applicable (no human/animal subjects directly studied).

Overall Assessment

- **STROBE Compliance %:** 83%
- **Quality Level:** High (though STROBE is not fully applicable to national-level epidemiological reviews).
- **Risk of Bias:** Medium (due to reliance on surveillance data, limited bias discussion, and restricted generalizability).
- **Strengths:** Clear objectives, comprehensive epidemiological overview, strong public health relevance.
- **Weaknesses:** Limited statistical analysis, incomplete bias discussion, restricted generalizability beyond China.

Publication 79

Epidemiology of Leptospirosis in Africa: A Systematic Review of a Neglected Zoonosis and a Paradigm for 'One Health' in Africa.

STROBE Item	Compliance	Observation
Title/Abstract	Yes	Clear, informative, specifies epidemiology of leptospirosis in Africa and One Health paradigm.
Background/Rationale	Yes	Provides epidemiological and public health context of leptospirosis in Africa.
Objectives	Yes	Explicit aim: systematically review epidemiology of leptospirosis in Africa.
Study Design	Partial	Systematic review; STROBE is designed for observational studies, so compliance is limited.
Setting	Yes	African countries and regional data sources described.
Participants	N/A	No individual-level participants; population-level studies reviewed.
Variables	Yes	Incidence, prevalence, geographic distribution, and risk factors defined.
Data Sources/Measurement	Yes	Literature search strategy, inclusion/exclusion criteria, and data extraction explained.

Bias	Partial	Potential publication and reporting bias acknowledged but not deeply analyzed.
Study Size	Partial	Number of studies included reported; justification of scope limited.
Quantitative Variables	Yes	Epidemiological metrics summarized.
Statistical Methods	Partial	Descriptive synthesis used; limited meta-analysis or inferential statistics.
Participants (Results)	N/A	Not applicable (review of published studies).
Descriptive Data	Yes	Clear breakdown of geographic distribution and epidemiological findings.
Outcome Data	Yes	Burden of leptospirosis in Africa confirmed.
Main Results	Yes	Leptospirosis widespread but underreported; One Health approach emphasized.
Other Analyses	Partial	Limited comparative or subgroup analyses beyond descriptive synthesis.
Key Results (Discussion)	Yes	Findings summarized clearly.
Limitations	Partial	Limitations of review design and data availability mentioned but not deeply elaborated.
Interpretation	Yes	Results interpreted in context of zoonotic disease control and One Health.
Generalizability	Partial	Findings relevant to Africa; external generalizability limited.
Funding	Yes	Funding sources acknowledged.
Ethical Considerations	N/A	Not applicable (review of published studies).

Overall Assessment

- **STROBE Compliance %:** 83%
- **Quality Level:** High (though STROBE is not fully applicable to systematic reviews).
- **Risk of Bias:** Medium (due to reliance on published studies, limited bias discussion, and restricted generalizability).
- **Strengths:** Clear objectives, comprehensive review of African epidemiology, strong emphasis on One Health paradigm.
- **Weaknesses:** Limited statistical synthesis, incomplete bias discussion, restricted generalizability beyond Africa.

Publication 80

Clinical leptospirosis in Kenya (2): A field study in Nyanza Province.

STROBE Item	Compliance	Observation
Title/Abstract	Yes	Clear, informative, specifies clinical leptospirosis field study in Nyanza Province, Kenya.
Background/Rationale	Yes	Provides epidemiological and clinical context of leptospirosis in East Africa.
Objectives	Yes	Explicit aim: investigate clinical leptospirosis in Nyanza Province.
Study Design	Yes	Field-based observational study described.
Setting	Yes	Rural Nyanza Province, Kenya; context explained.
Participants	Yes	Human patients included; inclusion/exclusion criteria explained.
Variables	Yes	Clinical symptoms, laboratory confirmation, demographic factors defined.
Data Sources/Measurement	Yes	Serological assays, clinical records, and field data explained.
Bias	Partial	Potential diagnostic and selection bias acknowledged but not deeply analyzed.
Study Size	Partial	Number of patients reported; justification of sample size limited.
Quantitative Variables	Yes	Clinical and laboratory variables analyzed.
Statistical Methods	Partial	Descriptive statistics used; limited inferential analysis.
Participants (Results)	Yes	Numbers and distribution of patients reported.
Descriptive Data	Yes	Clear breakdown of demographics, clinical features, and laboratory results.
Outcome Data	Yes	Clinical leptospirosis cases confirmed.
Main Results	Yes	Leptospirosis identified as a significant clinical problem in Nyanza Province.
Other Analyses	Partial	Limited subgroup analyses beyond descriptive epidemiology.
Key Results (Discussion)	Yes	Findings summarized clearly.
Limitations	Partial	Limitations of diagnostic methods and sample representativeness mentioned but not deeply elaborated.
Interpretation	Yes	Results interpreted in context of regional public health.
Generalizability	Partial	Findings relevant to Nyanza Province; external generalizability limited.

Funding	Yes	Funding sources acknowledged.
Ethical Considerations	Yes	Ethical approval obtained for human research.

Overall Assessment

- **STROBE Compliance %:** 86%
- **Quality Level:** High
- **Risk of Bias:** Medium (due to limited bias discussion, sample size justification, and restricted generalizability).
- **Strengths:** Clear objectives, field-based design, strong clinical relevance for East Africa.
- **Weaknesses:** Limited statistical analysis, incomplete bias discussion, restricted generalizability beyond Nyanza Province.

Publication 81

Clinical leptospirosis in Kenya (1): a clinical study in Kwale District, Coast Province.

STROBE Item	Compliance	Observation
Title/Abstract	Yes	Clear, informative, specifies clinical leptospirosis study in Kwale District, Kenya.
Background/Rationale	Yes	Provides epidemiological and clinical context of leptospirosis in coastal Kenya.
Objectives	Yes	Explicit aim: investigate clinical leptospirosis in Kwale District.
Study Design	Yes	Field-based observational study described.
Setting	Yes	Kwale District, Coast Province, Kenya; rural clinical context explained.
Participants	Yes	Human patients included; inclusion/exclusion criteria explained.
Variables	Yes	Clinical symptoms, laboratory confirmation, demographic factors defined.
Data Sources/Measurement	Yes	Serological assays, clinical records, and field data explained.
Bias	Partial	Potential diagnostic and selection bias acknowledged but not deeply analyzed.
Study Size	Partial	Number of patients reported; justification of sample size limited.
Quantitative Variables	Yes	Clinical and laboratory variables analyzed.
Statistical Methods	Partial	Descriptive statistics used; limited inferential analysis.
Participants (Results)	Yes	Numbers and distribution of patients reported.

Descriptive Data	Yes	Clear breakdown of demographics, clinical features, and laboratory results.
Outcome Data	Yes	Clinical leptospirosis cases confirmed.
Main Results	Yes	Leptospirosis identified as a significant clinical problem in Kwale District.
Other Analyses	Partial	Limited subgroup analyses beyond descriptive epidemiology.
Key Results (Discussion)	Yes	Findings summarized clearly.
Limitations	Partial	Limitations of diagnostic methods and sample representativeness mentioned but not deeply elaborated.
Interpretation	Yes	Results interpreted in context of regional public health.
Generalizability	Partial	Findings relevant to Kwale District; external generalizability limited.
Funding	Yes	Funding sources acknowledged.
Ethical Considerations	Yes	Ethical approval obtained for human research.

Overall Assessment

- **STROBE Compliance %:** 86%
- **Quality Level:** High
- **Risk of Bias:** Medium (due to limited bias discussion, sample size justification, and restricted generalizability).
- **Strengths:** Clear objectives, field-based design, strong clinical relevance for coastal Kenya.
- **Weaknesses:** Limited statistical analysis, incomplete bias discussion, restricted generalizability beyond Kwale District.

Publication 82

Leptospirosis in Ecuador: Current Status and Future Prospects.

STROBE Item	Compliance	Observation
Title/Abstract	Yes	Clear, informative, specifies leptospirosis epidemiology in Ecuador and future prospects.
Background/Rationale	Yes	Provides epidemiological and public health context of leptospirosis in Ecuador.
Objectives	Yes	Explicit aim: summarize current status and future directions for leptospirosis in Ecuador.
Study Design	Partial	Narrative/epidemiological review; STROBE is designed for observational studies, so compliance is limited.

Setting	Yes	National and regional epidemiological data sources described.
Participants	N/A	No individual-level participants; population-level surveillance data used.
Variables	Yes	Incidence, prevalence, geographic distribution, and risk factors defined.
Data Sources/Measurement	Yes	Surveillance records, published studies, and laboratory data explained.
Bias	Partial	Potential reporting and surveillance bias acknowledged but not deeply analyzed.
Study Size	Partial	National data sets referenced; sample size justification not applicable.
Quantitative Variables	Yes	Epidemiological metrics summarized.
Statistical Methods	Partial	Descriptive epidemiology used; limited inferential statistics.
Participants (Results)	N/A	Not applicable (population-level data).
Descriptive Data	Yes	Clear breakdown of geographic distribution and epidemiological findings.
Outcome Data	Yes	Burden of leptospirosis in Ecuador confirmed.
Main Results	Yes	Leptospirosis remains widespread; future prospects for surveillance and control discussed.
Other Analyses	Partial	Limited comparative or subgroup analyses beyond descriptive epidemiology.
Key Results (Discussion)	Yes	Findings summarized clearly.
Limitations	Partial	Limitations of surveillance data and reporting mentioned but not deeply elaborated.
Interpretation	Yes	Results interpreted in context of public health and zoonotic risk.
Generalizability	Partial	Findings relevant to Ecuador; external generalizability limited.
Funding	Yes	Funding sources acknowledged.
Ethical Considerations	N/A	Not applicable (review of published studies).

Overall Assessment

- **STROBE Compliance %:** 83%
- **Quality Level:** High (though STROBE is not fully applicable to national-level epidemiological reviews).
- **Risk of Bias:** Medium (due to reliance on surveillance data, limited bias discussion, and restricted generalizability).

- **Strengths:** Clear objectives, comprehensive epidemiological overview, strong public health relevance for Ecuador.
- **Weaknesses:** Limited statistical synthesis, incomplete bias discussion, restricted generalizability beyond Ecuador.

Publication 83

Draft Genome Sequence of the First Pathogenic *Leptospira* Isolates from Ecuador.

STROBE Item	Compliance	Observation
Title/Abstract	Yes	Clear, informative, specifies draft genome sequencing of pathogenic *Leptospira*isolates from Ecuador.
Background/Rationale	Yes	Provides microbiological and epidemiological context of leptospirosis in Ecuador.
Objectives	Yes	Explicit aim: sequence and describe the first pathogenic *Leptospira* isolates from Ecuador.
Study Design	Partial	Genomic sequencing report; STROBE is designed for observational studies, so compliance is limited.
Setting	Yes	Laboratory and national context explained.
Participants	N/A	No human/animal participants; bacterial isolates studied.
Variables	Yes	Genomic features, pathogenic markers, and sequencing data defined.
Data Sources/Measurement	Yes	Whole genome sequencing and bioinformatics pipelines explained.
Bias	Partial	Potential sequencing and assembly bias acknowledged but not deeply analyzed.
Study Size	Partial	Number of isolates reported; justification of sample size limited.
Quantitative Variables	Yes	Genomic metrics and sequence characteristics reported.
Statistical Methods	Partial	Descriptive genomic analysis; limited inferential statistics.
Participants (Results)	N/A	Not applicable (microbial isolates only).
Descriptive Data	Yes	Clear breakdown of genomic features and pathogenic markers.
Outcome Data	Yes	Draft genome sequences successfully obtained.

Main Results	Yes	First pathogenic *Leptospira* genomes from Ecuador reported.
Other Analyses	Partial	Limited comparative genomic analyses beyond descriptive sequencing.
Key Results (Discussion)	Yes	Findings summarized clearly.
Limitations	Partial	Limitations of draft genome quality and small isolate number mentioned but not deeply elaborated.
Interpretation	Yes	Results interpreted in context of molecular epidemiology and public health.
Generalizability	Partial	Findings relevant to Ecuador; external generalizability limited.
Funding	Yes	Funding sources acknowledged.
Ethical Considerations	N/A	Not applicable (microbial isolates, no human/animal subjects).

Overall Assessment

- **STROBE Compliance %:** 83%
- **Quality Level:** High (though STROBE is not fully applicable to genomic sequencing reports).
- **Risk of Bias:** Medium (due to limited bias discussion, small isolate number, and restricted generalizability).
- **Strengths:** Clear objectives, robust genomic sequencing methodology, novel contribution to Ecuadorian leptospirosis research.
- **Weaknesses:** Limited statistical analysis, incomplete bias discussion, restricted generalizability beyond Ecuadorian isolates.

Publication 84

Atypical leptospirosis: an overlooked cause of aseptic meningitis.

STROBE Item	Compliance	Observation
Title/Abstract	Yes	Clear, informative, specifies atypical leptospirosis presenting as aseptic meningitis.
Background/Rationale	Yes	Provides clinical and epidemiological context of leptospirosis and its atypical neurological manifestations.
Objectives	Yes	Explicit aim: describe a case of aseptic meningitis caused by leptospirosis.
Study Design	Partial	Case report; STROBE is designed for observational studies, so compliance is limited.
Setting	Yes	Hospital/clinical setting described.

Participants	Yes	One patient included; clinical details explained.
Variables	Partial	Clinical course and diagnostic findings described, but not structured as study variables.
Data Sources/Measurement	Yes	Laboratory assays, cerebrospinal fluid analysis, and diagnostic confirmation explained.
Bias	Partial	Case selection bias inherent; not deeply analyzed.
Study Size	N/A	Not applicable (single case report).
Quantitative Variables	Partial	Clinical parameters reported, but no statistical analysis.
Statistical Methods	N/A	Not applicable (case report).
Participants (Results)	Yes	Patient demographics and clinical presentation reported.
Descriptive Data	Yes	Clear breakdown of clinical course and laboratory findings.
Outcome Data	Yes	Diagnosis of leptospirosis-related aseptic meningitis confirmed.
Main Results	Yes	Leptospirosis can present atypically as aseptic meningitis.
Other Analyses	N/A	Not applicable (single case).
Key Results (Discussion)	Yes	Findings summarized clearly.
Limitations	Partial	Limitations of case report design mentioned but not deeply elaborated.
Interpretation	Yes	Results interpreted in context of atypical leptospirosis diagnosis.
Generalizability	Partial	Findings relevant to similar atypical cases; external generalizability limited.
Funding	Yes	Funding sources acknowledged.
Ethical Considerations	Yes	Ethical approval and patient consent obtained.

Overall Assessment

- **STROBE Compliance %:** 80%
- **Quality Level:** Moderate–High (appropriate for a case report, though STROBE is not fully applicable).
- **Risk of Bias:** High (single case, inherent selection bias, limited generalizability).
- **Strengths:** Clear clinical description, novel presentation of leptospirosis as aseptic meningitis, strong relevance for differential diagnosis in infectious neurology.
- **Weaknesses:** Case report design limits statistical rigor, bias discussion minimal, generalizability restricted to similar atypical cases.

Supplementary Table S3. Summary of the 84 scientific articles included in the systematic review and the extracted information on serological diversity, geographical distribution, reservoirs, natural sources of infection, and reported cases of human leptospirosis caused by *Leptospira santarosai*.

Article number	First Autor / Title	Publication year	Serological identification	Geographical distribution	Reservoirs	Natural sources of infection	Human leptospirosis
**1**	**Peláez et al.** [22]. Genetic diversity of *Leptospira* in northwestern Colombia: first report of *Leptospira santarosai* as a recognized leptospirosis agent.	2016	Yes	Yes	Yes	No data	Yes
**2**	**Restrepo-Lopez et al.** [28]. Malaria, Dengue Fever, and Leptospirosis in the Urabá Antioqueño Region, Colombia: Etiological and Molecular Characterization among Patients with Acute Undifferentiated Febrile Illness.	2025	No data	Yes	Yes	No data	No data
**3**	**Silva-Ramos et al.** [29]. Molecular Characterization of *Leptospira* Species among Patients with Acute Undifferentiated Febrile Illness from the Municipality of Villeta, Colombia.	2024	No data	Yes	Yes	No data	No data
**4**	**Perez-Garcia et al.** [30]. Canine Leptospirosis in a Northwestern Region of Colombia: Serological,	2022	No data	Yes	Yes	No data	No data

	Molecular and Epidemiological Factors.						
**5**	**Silva-Ramos et al.** [31]. Molecular Evidence of *Leptospira* spp. Infection Among Household Dogs From 15 Municipalities of the Department of Caldas, Colombia.	2025	No data	Yes	Yes	No data	No data
**6**	**Agudelo-Florez et al.** [32]. Genotipificación y evaluación de la dinámica de infección de un aislamiento colombiano de *Leptospira santarosai* en el modelo experimental en hámster.	2014	No data	Yes	Yes	No data	No data
**7**	**Uribe-Restrepo et al.** [33]. Clinical presentation of human leptospirosis in febrile patients: Urabá, Colombia.	2024	No data	Yes	Yes	No data	No data
**8**	**Hamond et al.** [34]. *Leptospira borgpetersenii* serovar Hardjo and *Leptospira santarosai* serogroup Pyrogenes isolated from bovine dairy herds in Puerto Rico.	2022	No data	Yes	Yes	No data	No data
**9**	**Miotto et al.** [16]. Molecular and serological characterization of the first *Leptospira santarosai* strain isolated from a dog.	2016	No data	Yes	Yes	No data	No data
**10**	**Diaz et al.** [35]. First detection of *Leptospira santarosai* in the	2022	No data	Yes	Yes	No data	No data

	reproductive track of a boar: A potential threat to swine production and public health.						
**11**	**Li-Fang et al.** [36]. Sequence of *Leptospira santarosai* serovar Shermani genome and prediction of virulence-associated genes.	2012	Yes	Yes	Yes	No data	No data
**12**	**Nogueira et al.** [37]. Draft-genome sequences of *Leptospira santarosai* strains isolated from urogenital tract of cows.	2023	Yes	Yes	Yes	No data	No data
**13**	**Moreno et al.** [38]. Characterization of *Leptospira santarosai* Serogroup Grippotyphosa Serovar Bananal Isolated from Capybara (Hydrochaeris hydrochaeris) in Brazil.	2016	Yes	Yes	Yes	No data	No data
**14**	**Hamond et al.** [39]. A multilocus variable number tandem repeat analysis assay provides high discrimination for genotyping *Leptospira santarosai* strains.	2015	Yes	Yes	Yes	No data	No data
**15**	**Li-Fang et al.** [40]. Potential impact on kidney infection: a whole-genome analysis of *Leptospira santarosai* serovar Shermani.	2014	Yes	Yes	Yes	No data	No data

**16**	**Hai Nguyen-Tran et al.** [21]. Use of Advanced Diagnostics for Timely Identification of Travel-associated *Leptospira santarosai* Infection in Four Adolescents Through Plasma Microbial Cell-free DNA Sequencing With the Karius Test.	2024	No data	Yes	Yes	No data	Yes
**17**	**Delgado et al.** [41]. New Genetic Variants of *Leptospira* spp Characterized by MLST from Peruvian Isolates.	2022	No data	Yes	Yes	No data	No data
**18**	**Pinto et al.** [42]. Plurality of *Leptospira* strains on slaughtered animals suggest a broader concept of adaptability of leptospires to cattle.	2017	No data	Yes	Yes	No data	No data
**19**	**Loureiro et al.** [43]. Molecular analysis of leptospires from serogroup Sejroe obtained from asymptomatic cattle in Rio de Janeiro Brazil reveals genetic proximity to serovar Guaricura.	2016	Yes	Yes	Yes	No data	No data
**20**	**Valverde et al.** [44]. New serovars of *Leptospira* isolated from patients in Costa Rica: implications for public health	2013	Yes	Yes	Yes	No data	No data

**21**	**Carmon-Gasca et al.** [18]. Detection of *Leptospira santarosai* and *L. kirschneri* in cattle: new isolates with potential impact in bovine production and public health.	2011	Yes	Yes	Yes	No data	No data
**22**	**Rivera et al.** [45]. Diversidad genética de aislamientos peruanos de *Leptospira* spp. mediante electroforesis en gel de campo pulsado.	2012	No data	Yes	Yes	Yes	No data
**23**	**Hamond et al.** [46]. Genotyping of *Leptospira* directly in urine samples of cattle demonstrates a diversity of species and strains in Brazil.	2016	No data	Yes	Yes	No data	No data
**24**	**Pascal Bourhy et al.** [47]. Serovar Diversity of Pathogenic *Leptospira* Circulating in the French West Indies.	2013	Yes	Yes	Yes	No data	No data
**25**	**Barbosa-Guedes et al.** [48]. Circulating *Leptospira* species identified in cattle of the Brazilian Amazon.	2019	No data	Yes	Yes	No data	No data
**26**	**Vieira et al.** [49]. Pathogenic *Leptospira* species are widely disseminated among small	2019	No data	Yes	Yes	No data	No data

	mammals in Atlantic Forest biome.						
**27**	**Miotto et al.** [50]. Prospective study of canine leptospirosis in shelter and stray dog populations: Identification of chronic carriers and different *Leptospira* species infecting dogs.	2018	No data	Yes	Yes	No data	No data
**28**	**Fornazari et al.** [17]. *Leptospira* reservoirs among wildlife in Brazil: Beyond rodents.	2018	No data	Yes	Yes	No data	No data
**29**	**Weiss et al.** [51]. An Extended Multilocus Sequence Typing (MLST) Scheme for Rapid Direct Typing of *Leptospira* from Clinical Samples.	2016	No data	Yes	Yes	No data	No data
**30**	**Barragan et al.** [52]. High *Leptospira* Diversity in Animals and Humans Complicates the Search for Common Reservoirs of Human Disease in Rural Ecuador.	2016	No data	Yes	Yes	No data	No data
**31**	**Jaeger et al.** [53]. Novel MLST sequence types of pathogenic *Leptospira* spp.: Opening the black box of animal leptospirosis in Brazil.	2019	No data	Yes	Yes	No data	No data
**32**	**Somjit C. et al.** [54]. Potentially Pathogenic *Leptospira* in the	2020	No data	Yes	Yes	Yes	No data

	Environment of an Elephant Camp in Thailand.						
**33**	**Dos santos Madeiros et al.** [55]. Small Mammals as Carriers/Hosts of *Leptospira* spp. in the Western Amazon Forest.	2020	No data	Yes	Yes	No data	No data
**34**	**Barbosa Guedes et al.** [56]. *Leptospira* strains isolated from cattle in the Amazon region, Brazil, evidence of a variety of species and serogroups with a high frequency of the Sejroe serogroup.	2021	No data	Yes	Yes	No data	No data
**35**	**Nogueira di Azevedo et al.** [57]. Characterization of leptospiral DNA in the follicular fluid of non-pregnant cows.	2020	No data	Yes	Yes	No data	No data
**36**	**Araujo Santos et al.** [58]. New insights on *Leptospira* sp. infection in ewes maintained in field semiarid conditions.	2022	No data	Yes	Yes	No data	No data
**37**	**Luiza Aymée et al.** [59]. *Leptospira* spp. strains associated with Bovine Genital Leptospirosis (BGL).	2022	No data	Yes	Yes	No data	No data
**38**	**Ruzie-Sabljie et al.** [60]. First Report on *Leptospira* Species Isolated from Patients in Slovenia.	2023	No data	Yes	Yes	No data	No data

**39**	**Aymée et al.** [61]. The role of *Leptospira santarosai* serovar Guaricura as agent of Bovine Genital Leptospirosis.	2022	Yes	Yes	Yes	No data	No data
**40**	**Chinchilla et al.** [11]. Phylogenomic of *Leptospira santarosai*, a prevalent pathogenic species in the Americas.	2023	Yes	Yes	Yes	No data	No data
**41**	**De Araujo Santos et al.** [62]. Follow-up investigation revealed that sheep may play an important role in the transmission of *Leptospira* spp. infection in Caatinga biome field conditions.	2024	No data	Yes	Yes	No data	No data
**42**	**Mosquera et al.** [63]. Mixed *Leptospira* infections in domestic animals from a rural community with high leptospirosis endemicity.	2024	No data	Yes	Yes	No data	No data
**43**	**Nogueira di Azevedo et al.** [64]. Genetic Diversity and Clonal Expansion of Pathogenic *Leptospira* in Brazil: A Multi-Host and Multi-Regional Panorama	2025	No data	Yes	Yes	No data	No data
**44**	**Nogueira di Azevedo et al.** [65]. Ecological range and host–biome associations of pathogenic	2025	No data	Yes	Yes	No data	No data

	Leptospira in Brazil: A One Health perspective from a tropical area.						
**45**	**Chih-Wei et al.** [12]. *Leptospirosis* in Taiwan – an underestimated infectious disease.	2006	Yes	Yes	No data	No data	Yes
**46**	**Jaeger et al.** [66]. VNTR analysis demonstrates new patterns and high genetic diversity of *Leptospira* sp. of animal origin in Brazil.	2018	No data	Yes	Yes	No data	No data
**47**	**Chinchilla et al.** [67]. In-house isolation protocol from human serum samples demonstrates the circulating of a broad diversity of *Leptospira* serogroups in Costa Rica.	2025	No data	Yes	Yes	No data	No data
**48**	**Valverde et al.** [68]. Arenal, a new *Leptospira* serovar of serogroup Javanica, isolated from a patient in Costa Rica.	2008	Yes	Yes	Yes	No data	No data
**49**	**Hua-Kung *Wang* et al.** [24]. Factor associated with severity and mortality in patients with confirmed leptospirosis at a regional hospital in northern Taiwan.	2020	Yes	Yes	Yes	No data	Yes
**50**	**Michael R. Wilson et al.** [26]. Actionable Diagnosis of	2014	No data	Yes	Yes	No data	Yes

	Neuroleptospirosis by Next-Generation Sequencing.						
**51**	**Hatem Kallel et al.** [69]. First report of human *Leptospira santarosai* infection in French Guiana.	2020	Yes	Yes	Yes	No data	Yes
**52**	**Ramos-Vasquez et al.** [19]. Isolation and molecular identification of *Leptospira santarosai* and *Leptospira interrogans* in equines from eastern Mexico.	2024	No data	Yes	Yes	No data	No data
**53**	**Kumari Snehkant Lata et al.** [70]. Whole genome sequencing and de novo assembly of three virulent Indian isolated of *Leptospira*.	2020	Yes	Yes	Yes	No data	No data
**54**	**Federico S. Kremer et al.** [71]. Draft Genome Sequences of *Leptospira santarosai* Strains U160, U164, and U233, Isolated from Asymptomatic Cattle.	2015	No data	Yes	Yes	No data	No data
**55**	**Shakila Sudarsshani et al.** [72]. Leptospirosis: A Potential Culprit for Chronic Kidney Disease of Uncertain Etiology.	2023	No data	Yes	Yes	No data	No data
**56**	**Vasconcellos et al.** [73]. Isolation of *Leptospira santarosai*, serovar	2001	Yes	Yes	Yes	No data	No data

	guaricura from buffaloes (Bubalus bubalis) in Vale do Ribeira, São Paulo, Brazil.						
**57**	**Lilenbaum et al.** [74]. Molecular characterization of the first leptospires isolated from goats in Brazil.	2015	Yes	Yes	Yes	No data	No data
**58**	**Baker et al.** [75]. Survey of Coyotes (Canis latrans) for Vector-Borne and Bacterial Pathogens in South Carolina and Tennessee, USA.	2025	No data	Yes	Yes	No data	No data
**59**	**Guzman et al.** [76]. Domestic dogs in indigenous Amazonian communities: key players in *Leptospira* cycling and transmission?	2024	No data	Yes	Yes	No data	No data
**60**	**Nogueira Di Azevedo et al.** [77]. Comparative genomics of *Leptospira santarosai* reveals genomic adaptations in bovine genital strains.	2025	No data	Yes	Yes	No data	No data
**61**	**Torres-Castro et al.** [78]. *Leptospira* patógena en murciélagos de Campeche y Yucatán, México.	2020	No data	Yes	Yes	No data	No data
**62**	**Chamidri Naotunna et al.** [79]. Etiological agents causing	2016	Yes	Yes	Yes	No data	No data

	leptospirosis in Sri Lanka: A review.						
**63**	**Loureiro et al.** [80]. Usage of a selective media (EMJH-STAFF) in primary culturing of pathogenic leptospires from bovine clinical samples.	2015	No data	Yes	Yes	No data	No data
**64**	**Gorman et al.** [20]. *Leptospira* enrichment culture followed by ONT metagenomic sequencing allows better detection of *Leptospira* presence and diversity in water and soil samples.	2022	No data	Yes	Yes	Yes	No data
**65**	**Patrick Hochedez et al.** [81]. Factors Associated with Severe Leptospirosis, Martinique, 2010–2013.	2015	No data	Yes	Yes	No data	No data
**66**	**Hochedez et al.** [82]. Outbreak of leptospirosis among canyoning participants, Martinique, 2011.	2013	No data	Yes	Yes	No data	No data
**67**	**Huang-Yu Yang et al.** [83]. Overlooked Risk for Chronic Kidney Disease after Leptospiral Infection: A Population-Based Survey and Epidemiological Cohort Evidence.	2015	Yes	Yes	Yes	No data	No data
**68**	**Ston et al.** [84]. Diverse lineages of pathogenic *Leptospira* species	2022	Yes	Yes	No data	Yes	No data

	are widespread in the environment in Puerto Rico, USA.						
**69**	**Severine Matheus et al.** [85]. Microbiological investigations of severe tropical infections in French Amazonia: a prospective pilot study of first-line tests and metagenomics.	2024	No data	Yes	Yes	No data	Yes
**70**	**Sato et al.** [13]. A systematic survey of environmental DNA in Palau's lakes and waterfalls reveals an increase in *Leptospira* levels after flooding.	2024	No data	Yes	No data	Yes	No data
**71**	**Chen-Yi Liao et al.** [23]. Acute Respiratory Distress Syndrome Manifested by Leptospirosis Successfully Teated by Extracorporeal Membrane Oxygenation (ECMO).	2015	No data	Yes	Yes	No data	Yes
**72**	**Roman-Cardenas et al.** [86]. Identificación molecular de *Leptospira* spp., presente en el ganado lechero del cantón Loja-Ecuador.	2016	No data	Yes	Yes	No data	No data
**73**	**Loureiro et al.** [87]. High frequency of leptospiral vaginal carriers among slaughtered cows.	2017	No data	Yes	Yes	No data	No data

**74**	**Gonzales et al.** [25]. Detection of Leptospirosis Genome from the Aqueous Humor of a Patient with Bilateral Uveitis.	2023	No data	Yes	Yes	No data	Yes
**75**	**Ganosa et al.** [88]. Determining risk for severe leptospirosis by molecular analysis of environmental surface waters for pathogenic *Leptospira*.	2006	No data	Yes	No data	Yes	No data
**76**	**Nogueira di azevedo et al.** [89]. Molecular Epidemiology of Pathogenic *Leptospira* spp. Infecting Dogs in Latin America.	2023	No data	Yes	Yes	No data	No data
**77**	**Yang et al.** [90]. Leptospirosis renal disease: understanding the initiation by Toll-like receptors.	2007	No data	Yes	Yes	No data	No data
**78**	**Weilin *Hu* et al.** [91]. *Leptospira* and leptospirosis in China.	2014	No data	Yes	Yes	No data	No data
**79**	**Allan et al.** [15]. Epidemiology of Leptospirosis in Africa: A Systematic Review of a Neglected Zoonosis and a Paradigm for 'One Health' in Africa.	2015	No data	Yes	Yes	No data	No data
**80**	**De Geus et al.** [92]. Clinical leptospirosis in Kenya (2): A field study in Nyanza Province.	1977	No data	Yes	Yes	No data	No data
**81**	**De Geus et al.** [93]. Clinical leptospirosis in Kenya (1): a	1977	No data	Yes	Yes	No data	No data

	clinical study in Kwale District, Coast Province.						
**82**	**Calvopiña et al.** [94]. Leptospirosis in Ecuador: Current Status and Future Prospects.	2023	No data	Yes	Yes	Yes	No data
**83**	**Barragan et al.** [95]. Draft Genome Sequence of the First Pathogenic Leptospira Isolates from Ecuador.	2016	No data	Yes	Yes	No data	No data
**84**	**Ning Wang et al.** [96]. Atypical leptospirosis: an overlooked cause of aseptic meningitis.	2016	No data	Yes	Yes	No data	Yes

Supplementary Table S4. Summary of geographic distributions of serovars associated with *Leptospira santarosai*. The table lists the countries where the species has been reported, the corresponding serovars, and the total number of serovars identified per country.

Countries	Serovars	Serovars number
Panamá	Balboa, Kobbe, Canalzonae, Abrahamson, Figeiro, Maru, May, Beye, Tropica, Pyrogenes, Weaveri, Gorgas, Shermani, Bravo, Chagres, Darien, Gatuni, Bataviae	18
Perú	Perú, Rioja, Naparuca, Tingomaria, Sanmartini, Bargonicas, Raparupae, Bagua, Cenepa, Machihuenga, Babudieri, Luis, Aguatia	13
Brazil	Guaricura, Carioca, Goiano, Brasiliensis, Bananal, Fluminensis, Rio	7
Trinidad and Tobago	Tabaquite, Caribe, Trinidad, Navet, Princestown	5
Colombia	Alice, Canalzonae, Beye, Babudieri	4
USA	Georgia, Atlantae, Baqueri, Afchafalaya, Princestown	4
Costa Rica	Arenal, Costa Rica, Corredores	3
Puerto Rico	Borincana, Alexi	2
Nicaragua	Varela, Rama	2
Indonesia (Asia)	Vataviae, Pyrogenes	2
Australia (Oceania)	Cremastos, Szwajizak	2
Taiwan (East Asia)	Sulzerae, Shermani	1
Sri Lanka (South Asia)	Alice	1
Denmark (Europe)	Dania	1
French West Indies	Tabaquite	1
México	Mini, Tarassovi	2
India (South Asia)	Huanuco	1
Ecuador	*Leptospira santarosai* (unidentified serovar)	-
Belize	*Leptospira santarosai* (unidentified serovar)	-
French Guiana	*Leptospira santarosai* (unidentified serovar)	-
Martinique	*Leptospira santarosai* (unidentified serovar)	-
Palau (Oceania)	*Leptospira santarosai* (unidentified serovar)	-
Thailand (Asia)	*Leptospira santarosai* (unidentified serovar)	-
Slovenia (Europe)	*Leptospira santarosai* (unidentified serovar)	-
United Kingdom	*Leptospira santarosai* (probable imported case, recent trips to America)	-
(Europe)	*Leptospira santarosai*	-

Supplementary Table S5. Summary of the representative host taxa, environmental sources, and accidental hosts (humans) reported to be infected with serovars of *Leptospira santarosai*.

Serovars	Sources	Serovar number
Atlantae, Darien, Gatuni, Rama, Bakeri, Atchafalaya, Luis, Machiguenga, Varela, Perú, Brasilienses, Rioja, Tingomaria, Ruparupae, Cenepa, Bagua.	Opossum (Didelphimorphia)	16
Fluminense, Bananal, Vargonicas, Shermani, Gorgas, Beye, Tropica, Chagres, Balboa, Kobbe, Canalzonae, Rio, Caribe.	Rodents (Rodentia)	13
Dania, Sanmartini, Goiano, Guaricura, Aguatia, Mini, Tarassovi.	Bovine (*Bos taurus*)	7
Sanmartini, Babudieri.	Pig (*Sus scrofa domesticus*)	2
Georgia	Raccoon (*Procyon lotor*)	1
Bananal	Capybara (*Hydrochoerus hydrochaeris*)	1
Guaricura	Buffalo (*Bubalus bubalis*)	1
Beye, Guaricura, Babudieri	Canine (*Canis lupus familiaris*)	1
Carioca	Goat (*Capra aegagrus hircus*)	1
Leptospira santarosai Serovar Naparuca	Galictis cuja	1
Leptospira santarosai (unidentified serovar)	Coatis (*Nasua*)	1
Leptospira santarosai (unidentified serovar)	Coyotes (*Canis latrans*)	1
Leptospira santarosai (unidentified serovar)	Equines (Equidae)	1
Leptospira santarosai (unidentified serovar)	Sheep (*Ovis aries*)	1
Leptospira santarosai (unidentified serovar)	*Marmosops ocellatus*	1
Leptospira santarosai (unidentified serovar)	*Marmosops paulencis*	1
Leptospira santarosai (unidentified serovar)	*Metachirus myosuros*	1
Leptospira santarosai (unidentified serovar)	*Metachirus nudicaudatus*	1
Leptospira santarosai (unidentified serovar)	*Monodelphis glirina*	1
Leptospira santarosai (unidentified serovar)	*Monodelphis peruviana*	1
Leptospira santarosai (unidentified serovar)	Nine-banded armadillo (*Dasypus novemcinctus*)	1
Leptospira santarosai (unidentified serovar)	*Nectomys squamipes*	1
Leptospira santarosai (unidentified serovar)	Wild Boar (*Sus scrofa*)	1
Leptospira santarosai (unidentified serovar)	Bats	1
**Serovars**	**Environmental sources**	
Maru<br>*Leptospira santarosai (unidentified serovar)*<br>*Leptospira santarosai (unidentified serovar)*	Water<br>Lakes and Waterfalls<br>Water and Soil	1
**Serovars**	**Accidental host**	
Kremastos, Abrahamson, Borincana, Figeiro, Arenal, May, Szwajizak, Tabaquite, Alexi, Alice, Costa Rica, Princestown, Pyrogenes, Trinidad, Navet, Sulzerae, Bataviae, Weaveri, Corredores, Bravo, Canalzonae, Shermani, Beye, Babudieri.	Human (*Homo sapiens*)	24

Supplementary Table S6. Summary of clinical presentations and complications described in patients infected with *Leptospira santarosai*. The table summarizes the country, the infecting species or serovar, the reported clinical presentations of the disease, including benign anicteric leptospirosis, Weil's syndrome, and severe pulmonary hemorrhagic syndrome (SPHS), as well as complications that may occur after infection such as myocarditis, uveitis, and neuroleptospirosis. It also lists the bibliographic sources from which the data were extracted.

**Location**	**Agent**	**Clinical presentations**			**Complications**			**Bibliographic source**
**Country**	**Species/Serovar**	**Benign anicteric form**	**Weil's syndrome**	**SPHS*³**	**Myocarditis**	**Uveitis**	**Neuro leptospirosis**	
Colombia	-*Leptospira santarosai* Serovar Canalzonae<br>-*Leptospira santarosai* Serovar Alice	Yes<br>Yes	Yes					Peláez et al. [22].
Taiwan	*Leptospira santarosai* Serovar Shermani	Yes	Yes	Yes				Yang Chih-Wei et al. [12].
Taiwan	*Leptospira santarosai* Serovar Shermani	Yes	Yes	Yes	Yes		Yes	Hua-Kung Wang *et al. [24].*
Taiwan	*Leptospira santarosai*	Yes					Yes	Ning Wang et al. [96].
Taiwan	*Leptospira santarosai* Serovar Shermani			Yes				Chen-Yi Liao et al. [23].

USA*[1]	*Leptospira santarosai*	Yes						Hai Nguyen-Tran et al. [21].
USA*[2]	*Leptospira santarosai*					Yes	Yes	Michael R. Wilson et al. [26].
USA	*Leptospira santarosai*	Yes				Yes		Gonzales *et* al. [25].
French Guiana	*Leptospira santarosai* Serogroup Sejroe	Yes						Hatem Kallel *et* al. [69].

*[1] imported case from Costa Rica and Belize.

*[2] imported cases from Puerto Rico.

*[3] Severe Pulmonary Hemorrhagic Syndrome.

Supplementary Table S7. Summary of the clinical manifestations and laboratory findings (blood cell count, kidney function, liver function, urine chemistry, lung function, inflammatory markers, and antibiotic response) reported in patients with leptospirosis caused by *Leptospira santarosai*. The table also lists the bibliographic sources from which the information was extracted.

**Symptoms/Sign**	**Laboratory findings**							**Bibliographic source**
	**Blood cell count**	**Kidney function**	**Liver function**	**Urine chemistry**	**Lung function**	**Inflammation markers**	**Antibiotic /Response**	
Fever, Chills, headache, Frequent cough, Nausea Inappetence, Generalized erythema, Tachycardia,	Leukocytosis<br><br>Hypocalcemia		GPT (Alanine Aminotransferase) augmented	Proteinuria<br><br>Hematuria		C-reactive protein (CRP), augmented		Peláez et al. [22].

Malaise, Ocular itching, Jaundice, Myalgia, Arthralgia, Vomit, Diarrhea, Abdominal pain, Nasal congestion, Red eyes, Gingival bleeding, Hepatomegaly, Retro-orbital pain, and Back pain								
Fever, malaise, polyarthralgia, myalgias, conjunctivitis, gastrointestinal symptoms, chest pain, headache, photophobia, eye burning sensation, rash, chills, nausea, vomiting,			ALT (U/L) / AST (U/L), increased	Oliguria Dark urine		Erythrocyte Sedimentation Rate (ESR) (mm/H)/CPR (mg/dL), augmented<br><br>Procalcitonin, augmented	Azithromycin/full recovery<br><br>Doxycycline/full recovery<br><br>Ciprofloxacin/full recovery	Hai Nguyen-Tran et al. [21].

diarrhea, emesis, night sweats, presyncope, and eye pain.								
Fever, jaundice, abdominal pain, myalgia, splenomegaly, hepatomegaly, acute respiratory failure, disturbed consciousness, hemorrhagic syndrome, acute pancreatitis, severe dyspnea, chills, migrating headache, flank pain, sinus bradycardia, hemorrhagic diathesis, bilateral enlarged	Hypocalcemia Leukocytosis Leukopenia Thrombocytopenia	Acute renal failure<br><br>Acute tubulo-interstitial nephritis<br><br>Interstitial edema	ALT (U/L) / AST (U/L), increased	Oliguria	Pulmonary hemorrhage<br><br>Bilateral diffuse pulmonary infiltrates		Penicillin/ full recovery	Chih-Wei et al. [12].

kidneys, sterile pyuria								
Cough, headache, tachycardia, pulmonary involvement, fever, dyspnea, myalgia, shock, jaundice, acute kidney injury, hemorrhage, meningitis, pulmonary lesions, shock, abdominal pain, vomiting, diarrhea, splenomegaly, arrhythmia, meningitis, and mortality.	White blood cell, augmented	Acute kidney injury	ALT (U/L) / AST (U/L), increased<br><br>Bilirubin, augmented	Proteinuria<br><br>Oliguria<br><br>Hematuria		C-reactive protein (CRP), augmented		Hua-Kung Wang et al. [24].
Severe combined immunodeficiency (SCID) headache, fever,	Thrombocytopenia, immune thrombocyt						Penicillin G/ full recovery	Michael R. Wilson et al. [26].

hydrocephalus, status epilepticus, medically induced coma, bilateral conjunctivitis, photophobia, pain with movement of his left eye, uveitis, daily frontotemporal headaches, fatigue, abdominal pain, weight loss, diffuse weakness, myalgias, nausea, vomiting, leptomeningitis with a granulomatous infiltrate.	openic purpura							
Dry cough, fever, myalgia,	Leukocytosis,	renal failure,	slightly elevated bilirubin levels,					Hatem Kallel et al. [69].

diffuse headache, hiccough, confusion, stiff neck, Glasgow coma score scale of 11/15, neurological failure, respiratory failure, and comatose state.	hyperlacticaemia, hemodynamic failure, decreased hematocrit, high lymphocytes, decreased platelets, decreased serum protein, decreased sodium, decreased potassium, decreased chlorides, decreased bicarbonates, decreased calcium, high glycemia,	high urea, high creatine,	and high gamma-glutamyl transferase (GGT).					

	and high lactate.							
Fever, cough, arthralgia, myalgia, pain in the neck and behind his eyes, rapid heart rate, peripheral vascular redness of both sclera, hypotension, hemoptysis, respiratory distress, intubated.	Thrombocytopenia, leukocytosis, marked elevation of neutrophils, high creatinine,		High aspartate aminotransferase (AST)	Mild proteinuria, leukocyturia, erythrocytosis	Acute respiratory failure, intrapulmonary seeding, progressive alveolar inflammation and refractory hypercapnia, significant pulmonary hemorrhage, The patient received ECMO treatment.	high C-reactive protein,	Penicillin G / full recovery<br><br>Levofloxacin / full recovery	Chen-Yi Liao et al. [23].

Bilateral chronic anterior and intermediate uveitis, multiple sclerosis, cloudy vision, iris heterochromia, fine keratic precipitates, 4+ anterior chamber cell, eye pigment, 2+ anterior vitreous cell with snowballs and a blunted foveal reflex, a quiet AC with extensive posterior synechiae and +0.5 anterior vitreous cells, cystoid macular edema.							Oral Doxycycline/ no response<br><br>Intravitreal dexamethasone/ resolution of uveitis	Gonzales et al. [25].
Aseptic meningitis, fever, nausea,	Leukocytosis, high					high C-reactive protein	Minocycline and ampicillin / full recovery	Ning Wang et al. [96].

vomiting, diffuse headache, disturbed sleep, neck stiffness, distressed facial appearance, increased intracranial pressure.	glucose (117 mg/dL)<br><br>Cerebrospinal fluid (CSF) pleocytosis							

* Severe Pulmonary Hemorrhage Syndrome (SPHS)
